# Long-Term Effectiveness of Unguided Internet-Based Cognitive Behavioral Therapy on Major Depressive Disorder in Chinese Adults: Randomized Controlled Trial With a 12-Month Follow-Up

**DOI:** 10.2196/68394

**Published:** 2026-06-24

**Authors:** Wenjing Zhou, Huimin Zhang, Yunbin Jiang, Yanzhi Li, Guangduoji Shi, Hao Zhao, Wanxin Wang, Yuhua Liao, Yifeng Liu, Jiejing Hao, Roger S McIntyre, Beifang Fan, Ciyong Lu

**Affiliations:** 1 Department of Medical Statistics and Epidemiology, School of Public Health, Sun Yat-sen University, 74 Zhongshan 2nd, Guangzhou, 510080, China, 86 13610355985; 2 Guangdong Provincial Key Laboratory of Food, Nutrition and Health, School of Public Health, Sun Yat-sen University, Guangzhou, China; 3 Department of Psychiatry, Shenzhen Nanshan Center for Chronic Disease Control, Shenzhen, China; 4 Department of Psychiatry, University of Toronto, Toronto, ON, Canada

**Keywords:** internet-based cognitive behavioral therapy, major depressive disorder, depression, China, randomized controlled trial, long-term effectiveness

## Abstract

**Background:**

Unguided internet-based cognitive behavioral therapy (ICBT) is a low-cost and scalable treatment for major depressive disorder (MDD), but its long-term effects in Chinese populations remain unclear.

**Objective:**

This study aimed (1) to explore the short- and long-term effectiveness of unguided ICBT in treating adults with MDD; (2) to investigate the short- and long-term effects on disease-related symptoms, individual and social functioning, and quality of life; and (3) to assess the acceptability and satisfaction with the ICBT.

**Methods:**

An 8-week randomized controlled trial (ChiCTR2100046425) was conducted between August 2021 and June 2023 in Shenzhen, China, with 159 participants in the immediate ICBT group (7-module ICBT course plus usual care) and 158 in the waitlist control (WLC) group (usual care). The WLC group later completed the same ICBT course and follow-up assessments. Outcome measures (depressive and anxiety symptoms, psychological distress, social functioning, self-efficacy, quality of life, and stigma) were assessed before and after treatment and at 3-, 6-, and 12-month follow-ups for ICBT participants. Remission and response, adherence, and satisfaction were evaluated by predefined standards.

**Results:**

Among 300 participants analyzed (mean age 28.49, SD 7.0 years; female: n=225, 75%), dropout rates were 22.4% (34/152) in the immediate ICBT group versus 6.3% (10/158) in the WLC group. At posttreatment, the immediate ICBT group showed greater reduction in depressive symptoms versus WLC (mean difference −3.65, SE 0.60; *P*<.001; *d*=0.50), with higher remission (80/121, 66.1% vs 58/148, 39.2%; *P*<.001) and response rates (50/121, 41.3% vs 27/148, 18.2%; *P*<.001). At 12-month follow-up, the depressive symptoms were improved compared with that at pretreatment (mean difference −3.90, SE 0.32; *P*<.001; *d*=0.70), and no significant change was observed in comparison with the outcomes at posttreatment (mean difference −0.81, SE 0.33; *P*=.33; *d*=−0.15). ICBT treatment also exhibited similar short- and long-term effects on secondary outcomes, with significant improvement of disease-related symptoms, individual and social functioning, and quality of life. Moreover, the majority of the participants treated with ICBT reported high acceptability of and satisfaction with the ICBT course.

**Conclusions:**

Unguided ICBT effectively reduces depressive symptoms and enhances functioning in Chinese patients with MDD, with sustained benefits over 12 months. Its scalability and low-cost nature make it a promising option for resource-limited settings.

## Introduction

### Background

Major depressive disorder (MDD) affects approximately 332 million people globally in 2021 [1]. As the second leading cause of nonfatal disability [1], MDD is associated with severe emotional symptoms, low self-efficacy, impaired social functioning, and diminished quality of life [2]. Additionally, MDD has imposed a significant economic burden on individuals and societies, which is predominantly from productivity loss [3,4]. Although there are efficacious treatments for MDD with robust clinical evidence (ie, psychotherapies and pharmacotherapies) [5], the treatment gap is still substantial. In low- and middle-income countries, more than 70% of patients remain untreated [6,7]. Social discrimination and personal stigma, high treatment expenditure, side effects of antidepressants, and the inconvenience of traditional in-person psychotherapies have prevented people from accessing these conventional forms of treatment [8].

Internet-based cognitive behavioral therapy (ICBT), a scalable, confidential, low-cost, and flexible modality of cognitive behavioral therapy (CBT) delivered through the internet with guided and unguided forms, has been introduced to the treatment of MDD to overcome the treatment barriers mentioned earlier [9]. Although CBT has been recommended as a first-line treatment for MDD by many treatment guidelines [10-12], the levels of evidence and applicable scenarios of ICBT vary across these guidelines. In China, mental health resources are severely limited, with per capita mental health investment standing at US $1.07 and only 2.19 certified psychiatrists per 100,000 people, far below the high-income country averages of US $35.06 and 13.06 psychiatrists [13]. The structural imbalance in the distribution further compounds the shortage, with hospitals and professionals disproportionately clustered in provincial capitals and the more developed eastern regions [14]. Moreover, despite the high prevalence of MDD in China (6.8%), the service coverage rate remains strikingly low at only 9.5% [15]. The unguided form, defined as self-help ICBT with different levels of technical support, offers greater potential for scalability compared to guided ICBT. Consequently, the earlier-mentioned mental health realities in China provided the impetus for our team to develop an unguided ICBT course for MDD called Morning Mood on WeChat Mini Program.

There is a growing body of evidence that unguided ICBT showed superior outcomes in alleviating depressive symptoms compared to control treatments (ie, treatment-as-usual and waitlist control [WLC]) in short-term clinical end points (ie, posttreatment) [16-31]. However, a large variance is observed in reported short-term effect sizes, ranging from no effect [23] to large effect sizes [22,28,29]. Moreover, the long-term efficacy of unguided ICBT remains understudied, with existing studies yielding inconsistent conclusions regarding whether sustained improvements occur beyond the intervention period [23,26,32-35]. Newby et al [26] found that the depressive outcomes at the posttreatment stage were favored over the 3-month follow-up stage, with a within-group effect size of −0.21, while Gilbody et al [23] reported that the within-group effect size between the posttreatment and 12-month follow-up stage was 0.35. Regarding binary outcomes, both response rates (defined as ≥50% reduction in symptom severity) and remission rates (defined as symptom levels below clinical thresholds) exhibited considerable variability across studies. Posttreatment analyses revealed that unguided ICBT achieved response rates of 37%‐58.6% and remission rates of 30.5%‐64%, representing more improvements over control conditions [18,20,25,27,32,34,36,37]. Only 1 study reported a response rate at 12-month follow-up of 51.4% [32]. In addition, existing studies focusing on unguided ICBT for MDD are largely represented by Western studies, thereby limiting the ability to generalize the results to patients from different races, ethnicities, and cultural backgrounds [38]. In China, only 1 randomized controlled trial (RCT) has verified the short-term effectiveness of clinician-supported unguided ICBT (the Chinese-translated version of MoodGYM) in patients with MDD [31]. However, previous research has explored the effect of ICBT for depressive symptoms or subthreshold depression. During the COVID-19 pandemic, unguided ICBT has been proven effective for patients with depressive symptoms and COVID-19 [39]. Another study demonstrated that unguided ICBT significantly reduced depressive severity among undergraduate students with mild to severe symptoms of depression [40]. Beyond unguided form, guided ICBT has also shown a moderate antidepressant effect among individuals with subthreshold depression, regardless of age [41,42]. Furthermore, a trial in Hong Kong confirmed that both web- and app-based guided ICBT led to significant improvements in depressive symptoms [43]. Furthermore, the aforementioned studies have primarily focused on depressive symptomatology outcomes, limiting possible insights into other relevant depression domains (eg, anxiety symptoms, self-efficacy, quality of life, and functional outcomes) [44]. Given the earlier-mentioned limitations, there is a need to evaluate the short- and long-term effectiveness of unguided ICBT for MDD, especially in China.

### Objectives

Herein, this study aimed (1) to evaluate the short- and long-term effectiveness of unguided ICBT in treating adults with MDD, (2) to further evaluate the short- and long-term effects on specific depressive symptoms as well as individual and social functioning, as well as quality of life, and (3) to assess the acceptability and satisfaction with the ICBT.

## Methods

### Study Design

This study was an 8-week nonblinded pragmatic RCT with 12-month follow-up, investigating whether an unguided ICBT course as an adjunct to usual care could reduce depression symptoms in adult patients with MDD and its long-term effectiveness. Eligible participants were randomized to either (1) the immediate ICBT group (ICBT group I), receiving the ICBT intervention with nonspecialists’ support plus usual care, or (2) the WLC group, receiving usual care only. After the 8-week control period, WLC participants crossed over to receive the ICBT intervention (ICBT group II). Both ICBT groups (I and II) were assessed at baseline, posttreatment, and at 3, 6, and 12 months after the intervention to examine immediate and long-term effects.

### Ethical Considerations

This trial was registered with the Chinese Clinical Trial Registry (ChiCTR2100046425). The study protocol, patient information sheets, and informed consent forms were approved by the Medical Ethics Committee of Shenzhen Nanshan Center for Chronic Disease Control, China (ethics approval ll20210012). The CONSORT (Consolidated Standards of Reporting Trials) with the eHealth extension was adopted to guide and report the trial (Checklist 1) [45]. All participants provided informed consent. Deidentified data were used for the whole analysis. Financial incentives (US $6.82 electronic cash) were distributed to those who completed the trial.

### Participants and Procedure

All participants were recruited from the Department of Depressive Disorder at Shenzhen Kangning Hospital and the Department of Psychiatry at Shenzhen Nanshan Center for Chronic Disease Control. MDD diagnosis was determined by using the Mini-International Neuropsychiatric Interview (M.I.N.I.), which was administered according to the *Diagnostic and Statistical Manual of Mental Disorders-Fourth Edition* (DSM-4) criteria [46,47]. The enrollment period spanned from August 2021 to June 2023. The key inclusion criteria were (1) Chinese residents, (2) aged between 18 and 60 years, (3) met criteria for MDD in DSM-4, (4) had access to the internet, (5) a score of Patient Health Questionnaire-9 (PHQ-9) more than 5, (6) no severe suicidal risk according to section C of the M.I.N.I., and (7) in a stable condition (if taking antidepressants, dosage had to be stable for at least 30 days before enrollment). The key exclusion criteria were (1) met DSM-4 criteria for bipolar disorder or other psychotic disorders, (2) had an ongoing alcohol or substance abuse disorder, (3) was pregnant or lactating, (4) underwent physiotherapy or psychotherapy treatments at the time of enrollment, and (5) any other condition deemed by the psychiatrists to interfere with ICBT (eg, cognitive impairment).

A 2-stage procedure was adopted for the selection of eligible participants. Outpatients who visited the department of depressive disorder and psychiatry were first screened by psychiatrists to identify potential study participants. Trained researchers (YJ and H Zhao) then conducted structured diagnostic interviews (ie, M.I.N.I.) to make the MDD diagnosis. Both researchers hold master's degrees and have medical backgrounds. They underwent standardized M.I.N.I. interview training organized by the National Center for Mental Disorders, which comprised theoretical instruction, simulated interviews, and a reliability test. Furthermore, both researchers completed a 1-year clinical practicum at the Department of Psychiatry, Nanshan Center for Chronic Disease Control (ie, Nanshan Mental Health Center). Throughout the data collection period, they received regular supervision from senior psychiatrists to ensure diagnostic consistency. Randomization was performed using a computer-generated sequence with randomly permuted block sizes (4 and 6). This sequence was managed centrally and applied sequentially to participants in biweekly batches throughout the recruitment period to ensure ongoing group balance. Due to the nature of the interventions, the blind method was hard to carry out.

Eligible participants were then instructed to complete baseline assessments through online questionnaires. Sociodemographic information (eg, age, sex, and ethnicity), lifestyle features (eg, exercise, drinking, and smoking), clinical characteristics (eg, antidepressant use, first of onset, and age of MDD onset), and baseline symptoms (eg, somatic pain, resilience, and insomnia severity) were collected.

### Interventions

The ICBT intervention in this trial was tailored for the Chinese population, aiming at teaching practical CBT skills for depression. A total of four phases were carried out for intervention development and cultural adaptation: (1) literature review on the ICBT intervention and cultural adaptation framework, (2) preliminary content setting, (3) face-to-face group discussion and content adaptation, and (4) expert consultations and finalized adaptation.

Guided by the ecological validity model, cultural adaptations were made across 8 dimensions (ie, language, persons, metaphors, content, concepts, goals, methods, and contexts) to ensure relevance, acceptability, and comprehensibility [48]. In terms of language, the intervention was delivered in Mandarin, using idioms and communication styles that align with local habits to prevent misinterpretation. For persons, all illustrative characters designed with names, appearances, and attire were in line with Chinese people’s characteristics, and video cases featured individuals from diverse age groups to enhance relatability. Metaphors were replaced with localized, everyday analogies to improve understanding. The content, including scenarios and activities, was tailored to align with common daily practices, social customs, and economic realities. Concepts of stressors and depressive symptoms were refined to resonate with the population’s lived experiences. To mitigate mental health stigma, the intervention’s goals were framed around “mood management” rather than depression treatment. The core therapeutic methods, such as behavioral activation, were preserved, but the suggested activities were customized according to culturally relevant options. Finally, the context of delivery was adapted by designing the intervention as a WeChat Mini Program, leveraging a ubiquitous platform in China to maximize accessibility and minimize barriers to engagement.

The ICBT course consists of 7 lessons, covering psychoeducation, behavioral activation, cognitive restructuring, affect regulation, and relapse prevention. The detailed content of each lesson and the corresponding screenshots are displayed in Multimedia Appendix 1. Participants in the ICBT group were required to complete 1 lesson per week; one week after completing the prior lesson, the subsequent lesson would be open automatically. This setting was used to prevent participants from completing multiple lessons in a short period, which might potentially impact the effectiveness. General support involving technical support and daily reminders provided by nonspecialists (eg, nurses, lay health workers, and social workers) via telephone or WeChat was applied to prompt engagement. The active treatment phase comprised 7 weeks, while the total intervention period, which included a final week for outcome consolidation, was defined as 8 weeks.

Participants in the WLC group were treated with usual care, and no additional care was offered during the 8-week study period. After completing the posttreatment assessment, participants in the WLC group were asked whether they were willing to receive ICBT intervention at no cost. The login accounts and passwords for Morning Mood were then provided to the intended individuals. All participants with antidepressant prescriptions were instructed to be on a stable dose for the past 4 weeks before enrollment and maintain the antidepressant regimen (unchanged drug type and dosage) during the intervention phase. Only necessary adjustments due to severe adverse reactions are permitted.

### Outcome Measures

The primary outcome was depressive symptoms measured by the Chinese version of PHQ-9, which was with good reliability and validity [49]. Its Cronbach α was 0.91 in this study. Remission was defined as a PHQ-9 score less than 10, where the threshold was set and adopted by the Improving Access to Psychological Therapies of the United Kingdom to distinguish clinical cases and noncases [50,51]. Response was defined as symptom improvement (ie, the reduction of PHQ-9 score of 50% or more) [34].

The secondary outcome measures included anxiety symptoms, nonspecific psychological distress, social function impairment, general self-efficacy, quality of life, and depression stigma, which were assessed by the Chinese version of the Generalized Anxiety Disorder-7 (GAD-7), the Kessler 10-Item Psychological Distress Scale (K-10), the Sheehan Disability Scale (SDS), the General Self-Efficacy Scale (GSES), the Short Form Six-Dimension (SF-6D), and Depression Stigma Scale (DSS), respectively. These measurement tools have good reliability and validity [52-57]. In this study, the corresponding Cronbach α was 0.94, 0.96, 0.94, 0.71, 0.95, and 0.84, respectively. All outcomes were evaluated at pre- and posttreatment, with further follow-up at 3, 6, and 12 months after ICBT intervention.

Participants’ satisfaction and acceptability for ICBT treatment were investigated after the treatment by the following three questions: (1) “Do you think the courses in this project have been helpful for you?” (2) “Are you satisfied with the courses in this project?” and (3) “Would you be willing to recommend this project to people in need?”

### Statistical Analysis

The sample size was determined by a priori power analysis. A total of 86 participants per group (n=172) was thought to be sufficient to detect a moderate between-group effect size (Cohen *d=*0.50, defined as the standardized difference between groups obtained by calculating the mean difference and dividing by the pooled SD) on repeated measures for PHQ-9, with a power of 90% and 2-tailed α of .05. The expected sample size was calculated based on a previous meta-analysis of individual patient data that investigated the effectiveness of unguided ICBT versus inactive controls for depressive symptoms [34]. Taking the attrition into consideration, the target sample size should be 228 patients when the anticipated dropout rate is set at 25% [39].

Participants who did not start lesson 1 in the immediate ICBT group and who could not be contacted in the WLC group after randomization were excluded from the main analysis, which followed the modified intention-to-treat (mITT) principle [58-60]. Sensitivity analyses were conducted in the intention-to-treat (ITT) sample (ie, all randomized participants) and the per-protocol set (PPS; ie, participants who completed all modules in the immediate ICBT group and who completed the posttreatment assessment in the WLC group). According to the statistical principles for clinical trials, the primary analysis in a clinical trial should adhere to the ITT principle by including all randomized participants. In practice, the mITT approach is often used as a refinement for ITT. This approach justifiably excludes a limited number of participants, such as those who violated major eligibility criteria, never received any dose of the study treatment, or provided no data after randomization [61]. Excluding patients who received no treatment is methodologically sound, as such analyses can serve as an unbiased estimator for the principal stratum effect (ie, the effect in the population of patients who would initiate treatment) [62]. Furthermore, the mITT analysis complements the ITT findings by providing a more precise estimate of the treatment effect among patients who actually started the intervention [58]. Missing data of posttreatment assessment in the ITT sample were addressed via multiple imputation using chained equations (*MICE* package in R [53]), with pooled estimates derived from Rubin’s rules [63,64]. Baseline characteristics and outcome measures were described as mean (SD) for continuous variables and frequencies (percentages) for categorical variables. The differences between the 2 groups were compared using 2-tailed *t* tests, chi-square tests, and Mann-Whitney tests.

The 8-week model (ie, random intercept linear mixed models [LMMs]) was applied to evaluate the effectiveness of the ICBT by estimating the mean between-group differences (the ICBT group I: n=152 and the WLC group: n=148) in 9 outcomes at the pre- and posttreatment time points (8 weeks). In this model, treatment groups, the time point of measurement, and the treatment by time point interaction were treated as fixed effects, and the individual patient effect was set as a random effect with a normal distribution and expected value 0. Parameters were estimated using maximum likelihood. In addition, the follow-up model (ie, marginal LMM with random intercept) was used to estimate the outcome changes in the ICBT group (ICBT group I: n=152 and ICBT group II: n=108). The fixed effect of time was used to estimate the marginal means of each outcome at 8 weeks, 3, 6, and 12-month follow-ups. Post-hoc pairwise comparisons of these marginal means across time points were conducted with Bonferroni correction. There were 18 models, one up to 8 weeks across arms (“8-week model”) and one including combined intervention group data (“follow-up model”) per outcome variable (depressive symptoms, anxiety symptoms, psychological distress, social function impairment, health-related quality of life, general self-efficacy, depression stigma, personal depression stigma, and perceived depression stigma). Model fit was evaluated using Schwarz’s Bayesian criterion. Within-group and between-group effect sizes (Cohen *d*) were calculated based on the method suggested for LMM analysis [65,66]. It was reported that the effect be categorized as small (>0.20), moderate (>0.50), and large (>0.80) according to Cohen *d* [67]. Sensitivity analyses for long-term effectiveness evaluation were conducted, respectively, among participants treated with ICBT in the ICBT group and the WLC group.

The remission and response rates in the ICBT and WLC groups were also estimated by treatment arms (ICBT vs WLC) and time points (posttreatment, 3-, 6-, and 12-month follow-ups). Chi-square tests were performed to assess the frequency differences in remission and response between the 2 groups. All analyses were performed using R language (version 4.2.2; R Foundation for Statistical Computing), and a *P* value less than .05 was considered statistically significant.

## Results

### Overview

**Figure 1.**
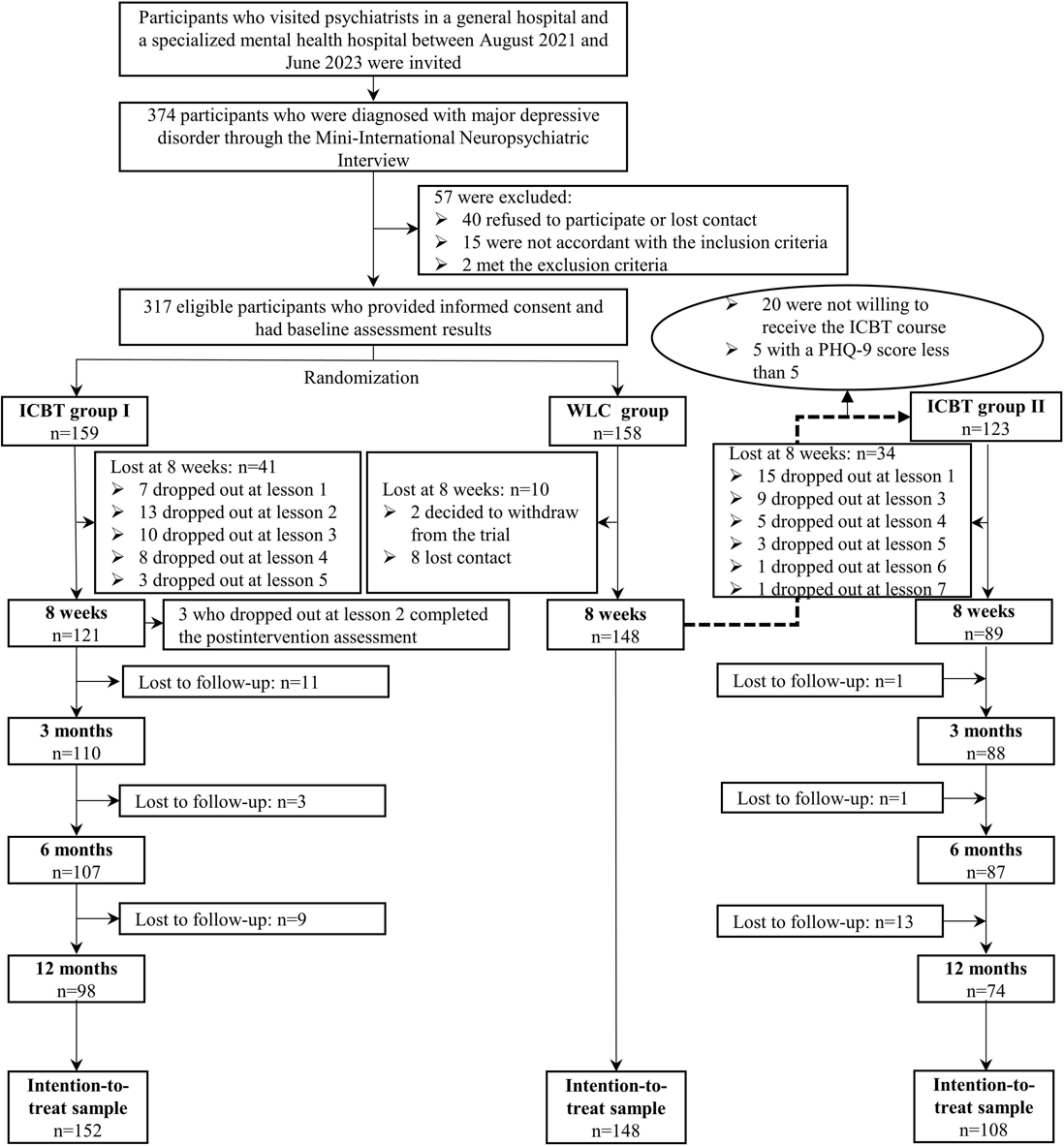
Study flowchart. ICBT: internet-based cognitive behavioral therapy; PHQ-9: Patient Health Questionnaire-9; WLC: waitlist control.

A total of 374 participants with MDD were recruited for this trial between August 2021 and June 2023, of whom 317 cases entered the trial and were randomized (ICBT group I: n=159; WLC group: n=158). In the ICBT group I, 7 participants did not start lesson 1 and thus were excluded. According to the criteria for completion and attrition defined in Multimedia Appendix 2, the treatment completion rate was 77.6% (118/152), and the attrition rate at 12-month follow-up was 35.5% (54/152). The average number of ICBT lessons completed was 5.89 (SD 2.13). In the WLC group, the dropout rate was 6.3% (10/158). After the 8-week waitlist phase, participants in the WLC group were provided access to the ICBT intervention (ICBT group II). In total, there were 108 (73%) who initiated the intervention. The remaining participants were excluded for the following reasons: 20 (13.5%) declined to receive treatment, 5 (3.4%) no longer met the inclusion criteria (PHQ-9 score<5), and 15 (10.1%) only registered at Morning Mood and did not start lesson 1. In this group, the treatment completion rate was 82.4% (89/108), and the attrition rate at 12-month follow-up was 31.5% (34/108). The detailed completion and dropout information are presented in Figure 1. The mITT sample used for the short-term effectiveness analyses consisted of 152 patients in the ICBT group I and 148 patients in the WLC group. For the long-term effectiveness evaluation, a total of 260 participants receiving ICBT intervention in the 2 ICBT groups were included, with 152 from group I and 108 from group II.

### Baseline Characteristics

Table 1 outlines the baseline characteristics of the mITT sample. The mean age was 28.49 (SD 7.00) years, and the female-to-male ratio was 3:1. Most participants were Han ethnic (283/298, 95%), highly educated (247/299, 82.6%), employed (192/297, 64.6%), and nonmarried (201/285, 70.5%). Participants whose monthly household income was below 10,000 Chinese Yuan (1 Chinese Yuan=US $0.1419 in 2023) accounted for the largest part (96/281, 34.2%). In modifiable lifestyle behaviors, the majority of the participants did not smoke cigarettes (186/299, 62.2%), lived with other people (210/283, 74.2%), drank alcohol (249/299, 83.3%), and had no exercise habits (218/299, 72.9%). In terms of clinical features, more than half of the participants were in their first episodes of MDD (175/292, 59.9%), did not take antidepressants (163/300, 54.3%), or did not have comorbidities at baseline (190/298, 63.8%). The mean (SD) of baseline outcome measures including PHQ-9, GAD-7, K-10, SDS, SF-6D, GSES, and DSS were 13.40 (4.83), 10.25 (4.60), 29.73 (7.48), 13.81 (6.81), 0.56 (0.21), 19.61 (5.71), and 52.08 (9.14), respectively. Baseline characteristics were comparable between the ICBT and WLC groups, suggesting that the randomization was successful. Table S1 in Multimedia Appendix 3 and Table S1 in Multimedia Appendix 4 display the baseline characteristics of the ITT sample and the PPS.

**Table 1.** Baseline characteristics of the participants included in the modified intention-to-treat sample.

Variable	Overall (N=300)	ICBT^a^ (n=152)	WLC^b^ (n=148)	*P* value^c^
Age (years), mean (SD)	28.49 (7.00)	29.05 (6.56)	27.92 (7.41)	.16
Sex, n (%)				.69
Male	75 (25)	40 (26.3)	35 (23.6)	
Female	225 (75)	112 (73.7)	113 (76.4)	
Nationality, n (%)				.98
Han	283 (95)	143 (95.3)	140 (94.6)	
Others	15 (5)	7 (4.7)	8 (5.4)	
Not available	2 (—^d^)	2 (—)	0 (—)	
Educational levels, n (%)				.63
High school or below	52 (17.4)	28 (18.4)	24 (16.3)	
Undergraduate	206 (68.9)	101 (66.4)	105 (71.4)	
Master degree or above	41 (13.7)	23 (15.1)	18 (12.2)	
Not available	1 (—)	0 (—)	1 (—)	
Employment status, n (%)				.06
Employed	192 (64.6)	106 (70.2)	86 (58.9)	
Unemployed	105 (35.4)	45 (29.8)	60 (41.1)	
Not available	3 (—)	1 (—)	2 (—)	
Marital status, n (%)				.43
Married	84 (29.5)	46 (31.9)	38 (27)	
Unmarried or divorced or widowed	201 (70.5)	98 (68.1)	103 (73)	
Not available	15 (—)	8 (—)	7 (—)	
Monthly household income, n (%)				.10
No fixed income	35 (12.5)	12 (8.3)	23 (16.8)	
Below 10,000 CNY^e^	96 (34.2)	52 (36.1)	44 (32.1)	
10,000‐20,000 CNY	77 (27.4)	45 (31.2)	32 (23.4)	
Above 20,000 CNY	73 (26)	35 (24.3)	38 (27.7)	
Not available	19 (—)	8 (—)	11 (—)	
Exercise, n (%)				.88
Yes	81 (27.1)	42 (27.8)	39 (26.4)	
No	218 (72.9)	109 (72.2)	109 (73.6)	
Not available	1 (—)	1 (—)	0 (—)	
Current drinking status, n (%)				.37
Yes	249 (83.3)	130 (85.5)	119 (81)	
No	50 (16.7)	22 (14.5)	28 (19)	
Not available	1 (—)	0 (—)	1 (—)	
Current smoking status, n (%)				.35
Yes	113 (37.8)	53 (34.9)	60 (40.8)	
No	186 (62.2)	99 (65.1)	87 (59.2)	
Not available	1 (—)	0 (—)	1 (—)	
Living alone, n (%)				.24
Yes	73 (25.8)	42 (29.2)	31 (22.3)	
No	210 (74.2)	102 (70.8)	108 (77.7)	
Not available	17 (—)	8 (—)	9 (—)	
Antidepressant use, n (%)				.36
Yes	137 (45.7)	65 (42.8)	72 (48.6)	
No	163 (54.3)	87 (57.2)	76 (51.4)	
First episode, n (%)				.92
Yes	175 (59.9)	89 (60.5)	86 (59.3)	
No	117 (40.1)	58 (39.5)	59 (40.7)	
Not available	8 (—)	5 (—)	3 (—)	
Age of onset (years), mean (SD)	24.51 (7.33)	25.05 (7.11)	23.94 (7.53)	.20
Comorbidity, n (%)				.53
Yes	108 (36.2)	52 (34.2)	56 (38.4)	
No	190 (63.8)	100 (65.8)	90 (61.6)	
Not available	2 (—)	0 (—)	2 (—)	
Number of SLE^f^, mean (SD)	1.85 (1.87)	1.91 (1.90)	1.79 (1.85)	.58
CTQ^g^, mean (SD)	49.00 (13.61)	49.99 (13.65)	47.98 (13.55)	.23
Emotional abuse scores, mean (SD)	10.60 (4.93)	10.56 (5.06)	10.64 (4.82)	.90
Physical abuse scores, mean (SD)	6.93 (3.15)	6.94 (3.24)	6.93 (3.06)	.97
Sexual abuse scores, mean (SD)	5.80 (1.94)	5.88 (2.12)	5.72 (1.75)	.48
Emotional neglect scores, mean (SD)	15.87 (5.22)	16.30 (5.04)	15.45 (5.38)	.18
Physical neglect scores, mean (SD)	9.94 (3.82)	10.30 (4.21)	9.58 (3.38)	.12
SSI^h^ scores, mean (SD)	63.29 (22.01)	63.34 (20.39)	63.24 (23.62)	.97
CD-RISC^i^ scores, mean (SD)	36.50 (14.87)	36.38 (14.60)	36.62 (15.19)	.90
RRS^j^ scores, mean (SD)	53.70 (10.17)	53.58 (10.35)	53.83 (10.03)	.83
ISI^k^ scores, mean (SD)	15.30 (6.61)	15.05 (6.30)	15.57 (6.93)	.50
PHQ-9^l^ scores, mean (SD)	13.40 (4.83)	13.91 (5.12)	12.87 (4.47)	.06
GAD-7^m^ scores, mean (SD)	10.25 (4.60)	10.63 (4.75)	9.86 (4.42)	.15
K-10^n^ scores, mean (SD)	29.73 (7.48)	30.28 (7.36)	29.18 (7.59)	.20
SDS^o^ scores, mean (SD)	13.81 (6.81)	15.19 (7.08)	12.40 (6.23)	<.001
GSES^p^ scores, mean (SD)	19.61 (5.71)	19.53 (5.34)	19.70 (6.08)	.80
SF-6D^q^ scores, mean (SD)	0.56 (0.21)	0.55 (0.21)	0.57 (0.20)	.26
DSS^r^ scores, mean (SD)	52.08 (9.14)	52.70 (8.98)	51.46 (9.28)	.25
Personal DSS scores, mean (SD)	22.65 (5.25)	22.97 (5.21)	22.33 (5.28)	.29
Perceived DSS scores, mean (SD)	29.43 (6.72)	29.73 (6.47)	29.14 (6.97)	.45

a ICBT: internet-based cognitive behavioral therapy.

b WLC: waitlist control.

c Baseline characteristics were compared between the 2 groups using 2 independent-sample 2-tailed * t* tests for continuous variables and chi-square tests or Fisher exact probabilities for categorical variables.

d Not applicable.

e CNY: Chinese Yuan (1 CNY=US $0.1419 in 2023).

f SLE: stressful life event.

g CTQ: Childhood Trauma Questionnaire.

h SSI: Somatic Symptom Inventory.

i CD-RISC: Connor-Davidson Resilience Scale.

j RRS: Ruminative Responses Scale.

k ISI: Insomnia Severe Index.

l PHQ-9: Patient Health Questionnaire-9.

m GAD-7: General Anxiety Disorder-7.

n K-10: Kessler 10-Item Psychological Distress Scale.

o SDS: Sheehan Disability Scale.

p GSES: General Self-Efficacy Scale.

q SF-6D: Short Form Six-Dimension.

r DSS: Depression Stigma Scale.

### Outcome Measures at Posttreatment

Table 2 depicts observed and estimated means of outcomes by treatment arms and time points, and the mean differences between groups calculated by 8-week models. The 8-week model suggested significant interaction effects of time-by-intervention-arm for PHQ-9 (*β*=−3.65; *P*<.001), GAD-7 (*β*=−2.85; *P*<.001), K-10 (*β*=−4.87; *P*<.001), SDS (*β*=−5.57; *P*<.001), GSES (*β*=1.42; *P*=.01), DSS (*β*=−2.93; *P*=.03), and personal DSS (*β*=−1.80; *P*=.008), except for SF-6D (*β*=.01; *P*=.50) and perceived DSS (*β*=−1.21; *P*=.21; Table S1 in Multimedia Appendix 5).

**Figure 2.**
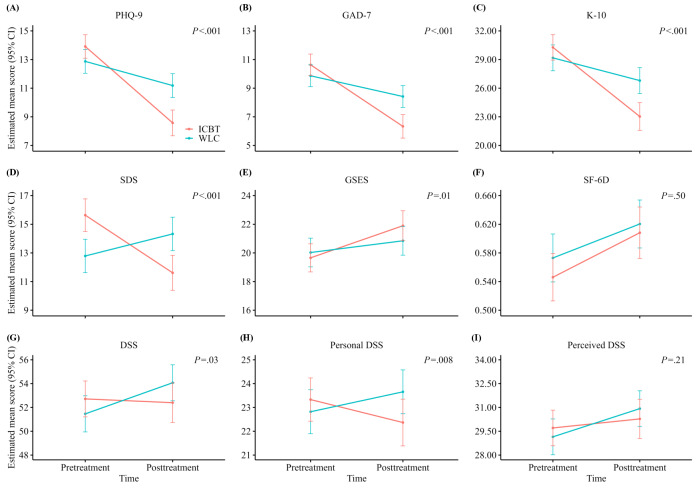
Estimated means and 95% CIs for time by treatment group interaction effects at 8 weeks. Linear mixed models with random intercept, including treatment groups (ie, internet-based cognitive behavioral therapy and waitlist control), time points of measurement (ie, pre- and posttreatment), and the treatment by time point interaction as fixed effects, are applied to calculate estimated means, mean differences in pre- and posttreatment changes between the 2 groups, and their *P* values. Patient-specific effects enter the model as a random effect with a normal distribution and an expected value of 0. DSS: Depression Stigma Scale; GAD-7: General Anxiety Disorder-7; GSES: General Self-Efficacy Scale; K-10: Kessler 10-Item Psychological Distress Scale; PHQ-9: Patient Health Questionnaire-9; SDS: Sheehan Disability Scale; SF-6D: Short Form Six-Dimension.

Figure 2 illustrates the change in estimated outcome measures across time by treatment arms. In both treatments, the scores of PHQ-9, GAD-7, and K-10 decreased over time, while the scores of SF-6D, GSES, and perceived DSS increased after treatment. Compared with the WLC group, larger improvements were obtained in these outcomes for those who received ICBT treatment. The changes in SDS, DSS, and personal DSS of the 2 groups moved in opposite directions, with a reduction in social function impairment, depression stigma, and personal stigma showing in the ICBT group.

**Table 2.** Outcomes for the modified intention-to-treat sample at pre- and posttreatment time points by treatment groups.

Outcome	Pretreatment						Posttreatment						Mean difference (SE)^c^	*P* value
	ICBT^a^			WLC^b^			ICBT			WLC				
	Values, n	Observed mean (SD)	Estimated mean (SE)^d^	Values, n	Observed mean (SD)	Estimated mean (SE)^d^	Values, n	Observed mean (SD)	Estimated mean (SE)^d^	Values, n	Observed mean (SD)	Estimated mean (SE)^d^		
PHQ-9^e^ scores	152	13.91 (5.12)	13.91 (0.42)	148	12.87 (4.47)	12.87 (0.42)	121	8.42 (5.70)	8.58 (0.46)	148	11.18 (5.37)	11.18 (0.42)	−3.65 (0.63)	<.001
GAD-7^f^ scores	152	10.63 (4.75)	10.63 (0.38)	148	9.86 (4.42)	9.86 (0.39)	121	6.36 (4.92)	6.33 (0.42)	148	8.42 (4.78)	8.42 (0.39)	−2.85 (0.56)	<.001
K-10^g^ scores	152	30.28 (7.36)	30.28 (0.68)	148	29.18 (7.59)	29.18 (0.69)	121	22.93 (9.72)	23.03 (0.74)	148	26.80 (8.99)	26.80 (0.69)	−4.87 (0.96)	<.001
SDS^h^ scores	152	15.19 (7.08)	15.19 (0.55)	148	12.40 (6.23)	12.40 (0.56)	119	11.03 (7.28)	11.18 (0.60)	148	13.94 (6.87)	13.94 (0.56)	−5.57 (0.72)	<.001
GSES^i^ scores	152	19.53 (5.34)	19.66 (0.50)	148	19.70 (6.08)	20.03 (0.51)	116	21.69 (5.84)	21.90 (0.53)	148	20.52 (6.22)	20.85 (0.51)	1.42 (0.56)	.01
SF-6D^j^ scores	152	0.55 (0.21)	0.55 (0.02)	148	0.57 (0.20)	0.57 (0.02)	116	0.61 (0.23)	0.61 (0.02)	148	0.62 (0.20)	0.62 (0.02)	0.01 (0.02)	.50
DSS^k^ scores	148	52.70 (8.98)	52.72 (0.77)	148	51.46 (9.28)	51.46 (0.77)	120	52.57 (10.45)	52.40 (0.85)	148	54.07 (8.89)	54.07 (0.77)	−2.93 (1.33)	.03
Personal DSS scores	148	22.97 (5.21)	23.02 (0.45)	148	22.33 (5.28)	22.31 (0.45)	120	22.18 (5.67)	22.07 (0.48)	148	23.15 (5.61)	23.15 (0.45)	−1.80 (0.68)	.008
Perceived DSS scores	148	29.73 (6.47)	29.71 (0.57)	148	29.14 (6.97)	29.15 (0.57)	120	30.39 (7.32)	30.28 (0.63)	148	30.93 (7.18)	30.93 (0.57)	−1.21 (0.95)	.21

a ICBT: internet-based cognitive behavioral therapy.

b WLC: waitlist control.

c The differences posttreatment to pretreatment outcomes between the 2 treatment groups are based on the difference of least square means of linear mixed models for repeated measures.

d Estimated means are based on linear mixed models.

e PHQ-9: Patient Health Questionnaire-9.

f GAD-7: General Anxiety Disorder-7.

g K-10: Kessler 10-Item Psychological Distress Scale.

h SDS: Sheehan Disability Scale.

i GSES: General Self-Efficacy Scale.

j SF-6D: Short Form Six-Dimension.

k DSS: Depression Stigma Scale.

The within- and between-group effect sizes measured by Cohen *d* were calculated based on the estimated means (Table S2 in Multimedia Appendix 5). For depressive symptoms, the between-group effect size was moderate (*d*=0.50; 95% CI 0.26‐0.75). The within-group effect size was large for the ICBT group (*d*=1.02; 95% CI 0.76‐1.27), whereas in the WLC group, the effect size was small (*d*=0.34; 95% CI 0.11‐0.57). For secondary outcomes, the between-group effect sizes were small (*d* range=0.07‐0.43). The within-group effect sizes in the ICBT group ranged from small to large, with GAD-7 (*d*=0.89; 95% CI 0.63-1.14) and K-10 (*d*=0.87; 95% CI 0.61-1.12) exhibiting large sizes, SDS (*d*=0.58; 95% CI 0.33-0.83) showing moderate size, and the left displaying small sizes (*d* range=0.01‐0.39). The within-group effect sizes in the WLC group were small, with a *d* range of 0.10-0.31.

Sensitivity analyses of short-term effectiveness outcomes using the ITT sample (Tables S2-S4 and Figure S1 in Multimedia Appendix 3) and the PPS population (Tables S2-S4 and Figure S1 in Multimedia Appendix 4) yielded results concordant with those of the primary analysis.

### Outcome Measures at Follow-Up

**Figure 3.**
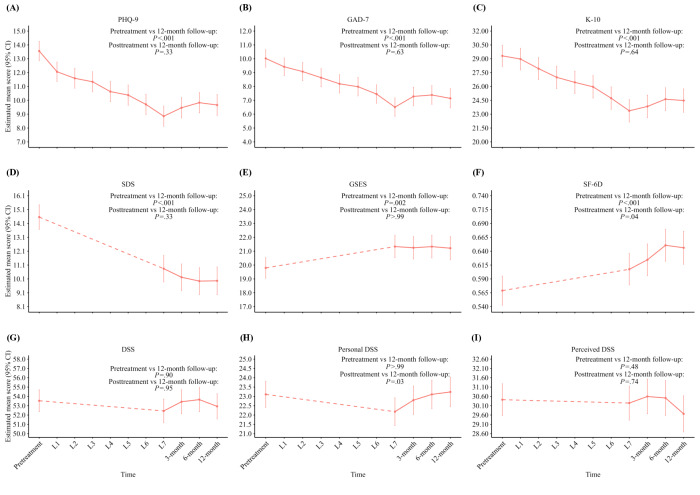
Estimated marginal means and 95% CIs across all time points for participants receiving ICBT intervention in both ICBT and waitlist control groups. Marginal linear models are applied to calculate estimated marginal means, mean differences, and their *P* values. Red dashed lines mean that no available data can be used for the intervention period. DSS: Depression Stigma Scale; GAD-7: General Anxiety Disorder-7; GSES: General Self-Efficacy Scale; ICBT: internet-based cognitive behavioral therapy; K-10: Kessler 10-Item Psychological Distress Scale; PHQ-9: Patient Health Questionnaire-9; SDS: Sheehan Disability Scale; SF-6D: Short Form Six-Dimension.

Table 3 presents the observed and estimated means of outcomes among all participants receiving ICBT treatment in the ICBT I and II groups by time points. Figure 3 demonstrates the change course of all outcome measures during the intervention and follow-up period. Follow-up models demonstrated that improvements in outcomes were maintained across 12-month follow-ups except for DSS and its subscales. The results of paired comparisons are displayed in Table S3 in Multimedia Appendix 5. Paired comparisons confirmed no significant change from posttreatment to 12-month follow-up on PHQ-9 (Bonferroni adjusted estimated mean difference −0.81; SE=0.33; *P*=.33), GAD-7 (Bonferroni adjusted estimated mean difference −0.63; SE=0.31; *P*=.63), K-10 (Bonferroni adjusted estimated mean difference −1.11; SE=0.55; *P*=.64), SDS (Bonferroni adjusted estimated mean difference 0.88; SE=0.47; *P*=.33), GSES (Bonferroni adjusted estimated mean difference 0.12; SE=0.38; *P*>.99), DSS (Bonferroni adjusted estimated mean difference −0.49; SE=0.69; *P*=.95), and perceived DSS (Bonferroni adjusted estimated mean difference 0.58; SE=0.47; *P*=.74), while significant increases in SF-6D (Bonferroni adjusted estimated mean difference −0.04; SE=0.01; *P*=.04) and personal DSS (Bonferroni adjusted estimated mean difference −1.06; SE=0.36; *P*=.03; Table 3).

**Table 3.** Observed and estimated means across time points in participants receiving ICBT treatment in the immediate ICBT^a^ and waitlist control group.

Outcomes	Pretreatment, T0^b^	Posttreatment, T1^c^	3 months, T2^d^	6 months, T3^e^	12 months, T4^f^	*F* test	*P* value^g^	*P* value (T0 vs T1)^h^	*P* value (T1 vs T4)^h^
Observed, mean (SD)						—^i^	—	—	—
PHQ-9^j^ scores	13.56 (4.96)	8.63 (5.56)	9.26 (5.56)	9.64 (5.95)	9.47 (5.90)				
GAD-7^k^ scores	10.02 (4.74)	6.46 (4.89)	7.24 (5.39)	7.30 (5.39)	7.08 (5.50)				
K-10^l^ scores	29.32 (7.85)	23.24 (9.26)	23.78 (9.64)	24.59 (10.05)	24.52 (9.94)				
SDS^m^ scores	14.56 (7.06)	10.65 (6.91)	9.96 (7.75)	9.72 (7.52)	9.58 (7.54)				
GSES^n^ scores	19.80 (5.54)	21.32 (5.74)	21.07 (6.24)	21.26 (6.64)	20.98 (6.92)				
SF-6D^o^ scores	0.57 (0.20)	0.61 (0.23)	0.62 (0.23)	0.65 (0.22)	0.64 (0.23)				
DSS^p^ scores	53.53 (8.92)	52.63 (10.16)	53.81 (9.50)	54.06 (9.92)	53.31 (10.13)				
Personal DSS scores	23.08 (5.29)	22.21 (5.63)	22.93 (5.73)	23.24 (6.14)	23.28 (6.41)				
Perceived DSS scores	30.45 (6.52)	30.42 (7.32)	30.87 (7.05)	30.82 (7.48)	30.03 (6.99)				
Estimated, mean (SE)^q^									
PHQ-9 scores	13.56 (0.35)	8.85 (0.37)	9.46 (0.37)	9.83 (0.38)	9.66 (0.39)	24.8(1181)	<.001	<.001	.33
GAD-7 scores	10.02 (0.32)	6.51 (0.34)	7.28 (0.34)	7.39 (0.34)	7.14 (0.35)	21.2 (1180)	<.001	<.001	.63
K-10 scores	29.32 (0.59)	23.37 (0.62)	23.84 (0.62)	24.63 (0.63)	24.47 (0.64)	21.8 (1179)	<.001	<.001	.64
SDS scores	14.56 (0.45)	10.84 (0.49)	10.22 (0.49)	9.94 (0.49)	9.96 (0.51)	22.2 (448)	<.001	<.001	.33
GSES scores	19.80 (0.38)	21.34 (0.41)	21.25 (0.41)	21.33 (0.42)	21.22 (0.43)	7.39 (449)	<.001	<.001	>.99
SF-6D scores	0.57 (0.01)	0.61 (0.01)	0.62 (0.01)	0.65 (0.01)	0.65 (0.02)	9.11 (450)	<.001	.02	.04
DSS scores	53.53 (0.60)	52.44 (0.64)	53.43 (0.66)	53.66 (0.66)	52.93 (0.69)	.35 (457)	.84	.42	.95
Personal DSS scores	23.11 (0.36)	22.18 (0.38)	22.80 (0.39)	23.11 (0.39)	23.24 (0.40)	2.11 (446)	.08	.046	.03
Perceived DSS scores	30.42 (0.43)	30.24 (0.47)	30.60 (0.47)	30.52 (0.48)	29.66 (0.49)	.99 (456)	.41	.99	.74

a ICBT: internet-based cognitive behavioral therapy.

b T0: pretreatment.

c T1: posttreatment.

d T2: 3-month follow-up.

e T3: 6-month follow-up.

f T4: 12-month follow-up.

g *P* values were associated with the test (*F* test, fixed effects) of the overall time period difference.

h *P* values adjusted by the Bonferroni method are shown.

i Not applicable.

j PHQ-9: Patient Health Questionnaire-9.

k GAD-7: General Anxiety Disorder-7.

l K-10: Kessler 10-Item Psychological Distress Scale.

m SDS: Sheehan Disability Scale.

n GSES: General Self-Efficacy Scale.

o SF-6D: Short Form Six-Dimension.

p DSS: Depression Stigma Scale.

q Estimated means are based on the follow-up models.

The within-group effect sizes for different outcome measures across time points are presented in Table S4 in Multimedia Appendix 5. During the follow-up period, the effect sizes for PHQ-9 were maintained at moderate levels (*d*≥0.50) compared with that of the pretreatment stage. For GAD-7, K-10, and SDS, moderate effect sizes were also observed over 12 months (*d* range=0.52‐0.73). The remaining secondary outcomes (eg, SF-6D and GSES) had shown small effect sizes both at posttreatment and follow-ups. Compared with the posttreatment stage, the effect sizes for all outcomes at follow-up were small, ranging from 0.01 to 0.18.

The results of the 2 sensitivity analyses for the long-term effectiveness evaluation were almost consistent with those of the main analysis (Tables S5-S8 and Figures S1 and S2 in Multimedia Appendix 5). For personal DSS, both the main analysis and sensitivity analyses showed down first and then up, with only the main analysis reporting statistical significance.

### Remission and Response

Table S9 in Multimedia Appendix 5 displays the numbers and rates of participants reaching remission from depression and responding to treatment by trial arms and across time points. At posttreatment, a total of 80 complete remissions and 50 responders were observed in 152 patients who received ICBT treatment. The remission and response rates reached 66.1% (80/121) and 41.3% (50/121), respectively. The clinical symptomatic remissions in the WLC group were significantly lower than those of the ICBT group, with complete remissions in 58 (39.2%) cases (*P*<.001) and response in 27 of the 148 (18.2%) patients (*P*<.001). During the follow-up period, the remission and response rates in the ICBT group remained high, with rates of 58.2% and 32.7%.

### Treatment Acceptability and Satisfaction

A total of 119 participants completed the course evaluation regarding perceived helpfulness, satisfaction, and recommended intention. In total, 98 (82.4%) reported that the course was helpful, 117 (98.3%) reported feeling satisfied with the course, and 111 (93.2%) participants were willing to recommend the course to others around them.

## Discussion

### Principal Findings

This study assessed the short- and long-term effectiveness of an unguided ICBT course in Chinese patients with MDD and evaluated participants’ acceptability and satisfaction with ICBT. Compared to WLC, depressive symptoms, disease-related symptoms, individual and social functioning, and quality of life in the ICBT group were significantly improved at the posttreatment stage, with the between-group effect size of PHQ-9 reaching 0.50. Remission and response rates of the ICBT group were statistically higher than those of the WLC group (80/121, 66.1% vs 58/148, 39.2%; *P*<.001; 50/121, 41.3% vs 27/148, 18.2%; *P*<.001). Moreover, these positive results in the ICBT group were maintained after the intervention. At 12-month follow-up, the depressive symptoms were improved compared with that at pretreatment (mean difference 3.90, SE 0.32; *P*<.001; *d*=0.70), and no significant change was observed in comparison with the outcomes at posttreatment (mean difference −0.81, SE 0.33; *P*=.33; *d*=−0.15). Patients treated with ICBT reported high acceptability with an engagement rate of 77.6%. Above 90% of participants in the ICBT group reported high treatment satisfaction. To our knowledge, this was the first study that investigated both the short- and long-term effectiveness of unguided ICBT supported by nonspecialists for MDD in China.

### Comparisons With Previous Work

The result that unguided ICBT as an additional treatment to usual care was more effective than usual care alone at posttreatment was well supported by the findings of most previous meta-analyses and RCTs on unguided ICBT [16,17,30,31,34]. The demonstrated effectiveness of the Morning Mood for Chinese clients with MDD can be attributed to its strict adherence to CBT principles and thoughtful cultural adaptation. First, the program successfully delivers core CBT components in a structured, sequential manner. This allows users to systematically learn and practice skills to break the cycle of negative thoughts and behaviors that maintain depression, even without a therapist guiding each step. Second, the program was delivered in the participants’ native language, ensuring clarity and facilitating a deeper understanding of complex psychoeducational content. Third, culturally resonant materials were used, such as incorporating familiar idioms and real-life scenarios that reflect the typical stressors and automatic thoughts experienced by the mainland Chinese population. This likely fostered a stronger connection with the content and improved the self-application of CBT skills. Fourth, the program’s design aligns with modern digital consumption habits, prioritizing video-based demonstrations and interactive exercises over text-heavy narratives, which suits contemporary learning preferences. Finally, the integration of mindfulness and relaxation exercises resonated with the traditional Chinese philosophy of mind-body harmony, thereby complementing the Western CBT framework and making the therapeutic concepts more intuitively understandable and personally meaningful to the target audience.

The ICBT group exhibited a statistically significant improvement in depressive symptoms than the WLC group, with a medium between-group size of 0.50. However, the effect size calculated in this study was lower than that of a meta-analysis (0.65) [17]. The relatively poor performance could be attributed to the supporting provider and implementing environment, which led to different treatment effects [16]. Previous investigations usually provided support by clinicians and were implemented in clinical settings [68], while this study differed by providing support through nonspecialists and being conducted in a more practical setting. It is acknowledged that unguided ICBT with clinician support leads to better treatment compliance due to the authority of doctors [23,42]. Moreover, the clinical setting has contextual factors, favoring the deployment and uptake of the intervention [51]. Therefore, higher effect sizes were reported in previous research. However, given the shortage of mental health specialists in China, unguided ICBT supported by nonspecialists might be more pragmatic and workable, with the potential of increasing accessibility, availability, and affordability of MDD treatment. More measures should be taken to enhance treatment outcomes when putting Morning Mood into large-scale use.

This study has also proven the sustained long-term effectiveness within the ICBT treatment conditions, with no significant difference in depressive symptoms between the posttreatment and follow-up stages. High remission and response rates were also observed both at posttreatment and follow-up under the ICBT intervention. This finding was consistent with the existing research [18-20,22,25,26,28,29]. However, 1 meta-analysis [35] and 2 large RCTs [21,23] reported a significantly large magnitude of effect at follow-up compared to the posttreatment outcome despite the varied within-group effect size. This could be partially explained by the study characteristics. In our study, patients were diagnosed with MDD, and more than half of them had antidepressant treatments, indicating a more severe disease condition. It is mentioned that the short- and long-term treatment effects of unguided ICBT are better in individuals with mild to moderate depressive symptoms [34]. The long-term effect of ICBT on depressive symptoms and absolute treatment outcomes suggested that intensified measures might be needed to consolidate the desired therapeutic effects for the long term in our future application.

Our results showed that at posttreatment, participants in the ICBT group experienced a significantly larger reduction of anxiety symptoms, nonspecific psychological distress, and depression stigma than individuals in the WLC group, while a greater improvement of self-efficacy and social functioning. Existing research on ICBT for MDD reported similarly favorable effects except for depression stigma [69-73]. These results provided new supportive evidence for the addition of unguided ICBT to usual care in developing countries.

We also found that ICBT produced long-term positive effects on anxiety symptoms, nonspecific distress, social functioning, general self-efficacy, and quality of life. However, few studies have comprehensively investigated these outcomes in the long run. As comorbidity depression with anxiety and other psychiatric disorders are common [74,75], ICBT interventions for depression have been proposed to be potentially effective in improving general mental health, including anxiety symptoms and nonspecific distress, which aligns with the findings in previous research [20,30,32]. General self-efficacy, which essentially reflects the subjective thoughts of participants, was considered to be an indicator of cognitive and behavioral changes in patients with MDD. It was found that ICBT intervention has significantly increased the individuals’ self-efficacy even after the acute treatment phase. The ultimate aim of the ICBT intervention is to develop the psychological and emotional skills to restore social functioning and improve quality of life. This self-developed ICBT course has finally reached its goals, which agrees with the results of prior research [23,32,35]. Together, these results indicate that ICBT for MDD has widespread favorable outcomes.

The majority of ICBT completers were satisfied with the course, and the engagement rate of the ICBT intervention in this study was high, with 77.6% (118/152) of individuals completing the course. A previous study reported that the average completion rate of a self-guided intervention with general support was 65% [16]. Several factors could account for the notably higher rate in our study. Compared with clinicians, nonspecialists have more time and energy to dedicate to the provision of general support to prompt engagement. Furthermore, the ICBT course in our study was designed for Chinese people with good cultural adaptability and was embedded on the WeChat app with good user convenience. Taken together, these findings showed that unguided ICBT supported by nonspecialists could enhance the treatment effect in multiple dimensions.

### Implications

This trial provides evidence on the short- and long-term effectiveness of the unguided ICBT intervention (ie, Morning Mood) for MDD in China across multiple dimensions: depressive symptoms, disease-related symptoms, psychosocial functioning, and quality of life. The high adherence rate (77.6%) in our study suggests that the program’s delivery format and support intensity align well with the needs of individuals with MDD in China. The unguided ICBT supported by nonspecialists shows a positive effect for MDD in conditions that require fewer resources, with the cost-effectiveness being proven in a previous study [76]. Therefore, it is indicated that integrating the unguided ICBT into the primary community health centers or other care pathways is a good choice in China, where trained therapists are limited, social discrimination is serious, and the accessibility of mental health care services is poor [77]. Second, the small to moderate between-group effect sizes in short-term outcomes emphasize the necessity of optimizing the efficacy by recognizing those who respond well to the intervention and those who show no response to treatment, which could further increase the cost-effectiveness [78]. Third, the suboptimal enduring remission rate (58.2%) underscores proactive monitoring in real-world applications to take timely intervention measures and prevent further deterioration.

### Strengths and Limitations

This study has several strengths. First, the ICBT program in this study is developed based on Chinese culture and habits of Chinese expressions, with examples demonstrated in the course being selected from real life; thus, the effect of cultural and linguistic incompatibility was excluded. Moreover, the ICBT intervention is delivered through the WeChat Mini Program, which makes the implementation more convenient than web- or app-based forms. Participants do not need to download an additional app or search on the Internet. To some extent, this study has provided more robust evidence of ICBT for depression in China. Second, in our study, the unguided ICBT intervention is supported by nonspecialist health workers (ie, any type of health worker, like a nurse, lay health worker, or medical social worker, who is not a specialist in mental health). This form of ICBT has no requirement for qualified mental health specialists and thus is easier to implement. The results of our study provide a reference for solving the problem of the treatment gap in resource-limited countries like China. Third, measurement tools with good reliability and validity have been used to evaluate the short- and long-term effectiveness of ICBT from multiple dimensions, including depressive symptoms, disease-related symptoms, individual and social functioning, and quality of life. Few previous studies have comprehensively explored these perspectives.

There are also some limitations in this study. First, due to ethical considerations, participants in the WLC group are arranged to receive ICBT treatment after an 8-week intervention period. That is, no control exists during the follow-up phase. As a result, it is difficult to distinguish the effect of ICBT from usual care and the natural course. A further RCT with long-term follow-ups in all treatment arms should be conducted to solve this problem. Second, the participants in our study were recruited from mental health clinics in Shenzhen, China, and were diagnosed with MDD. The majority of them were female and received high levels of education. Therefore, the generalization of the present findings may be limited. Third, due to data qualification of the sample, we could only compare the proportions of participants taking antidepressants in the ICBT and WLC groups. It is thought that drug types, dosage, and duration influence the treatment outcomes.

### Conclusions

This study has demonstrated the short- and long-term effectiveness of unguided ICBT with general support delivered by nonspecialists for patients with MDD from multiple dimensions, including depressive symptoms, anxiety symptoms, nonspecific psychological distress, social functioning, quality of life, and general self-efficacy. Participants report high acceptability of and satisfaction with the ICBT course. Taken together, these results add to existing literature consolidating unguided ICBT supported by nonspecialists as an effective treatment to solve the substantial treatment discrepancies in low-resource settings. Additional studies with larger samples are needed to examine potential factors that could enhance the long-term effectiveness of ICBT.

## Supplementary material

10.2196/68394 Multimedia Appendix 1 Outline of the treatment content, homework, and corresponding screenshots of the internet-based cognitive behavioral therapy program.

10.2196/68394 Multimedia Appendix 2 Criteria for defining treatment completion and participant attrition.

10.2196/68394 Multimedia Appendix 3 Sensitivity analysis in the intention-to-treat sample.

10.2196/68394 Multimedia Appendix 4 Sensitivity analysis in the per-protocol set.

10.2196/68394 Multimedia Appendix 5 Supplementary results of the main analysis.

10.2196/68394 Checklist 1 CONSORT-eHEALTH checklist (V 1.6.1).

## Abbreviations

CBT: cognitive behavioral therapy
CONSORT: Consolidated standards of reporting trials
DSM-4: Diagnostic and Statistical Manual of Mental Disorders, Fourth Edition
DSS: Depression Stigma Scale
GAD-7: Generalized Anxiety Disorder-7
GSES: General Self-Efficacy Scale
ICBT: internet-based cognitive behavioral therapy
ITT: intention-to-treat
K-10: Kessler 10-Item Psychological Distress Scale
LMM: linear mixed model
M.I.N.I.: Mini-International Neuropsychiatric Interview
MDD: major depressive disorder
mITT: modified intention-to-treat
PHQ-9: Patient Health Questionnaire-9
PPS: per-protocol set
RCT: randomized controlled trial
SDS: Sheehan Disability Scale
SF-6D: Short Form Six-Dimension
WLC: waitlist control
