# Supplementary material for: Long-Term Effectiveness of Unguided Internet-Based Cognitive Behavioral Therapy on Major Depressive Disorder in Chinese Adults: Randomized Controlled Trial With a 12-Month Follow-Up
Source: JMIR Mhealth Uhealth. 2026 Jun 24;14:e68394. doi: 10.2196/68394 (PMC13293601; doi:10.2196/68394)

| **Module** | **Treatment Theme** | **Content** | **Duration** | **Homework** |
| --- | --- | --- | --- | --- |
| 1 | Psychoeducation | Psychoeducation about depression, CBT^a^, the thoughts-feelings-actions cycle and events record | 36 mins | Pleasant events record |
| 2 | Recording activities and identifying your depression | Strengthening the concept of CBT triad; learning how to set goals, record activities and automatic thought; identifying specific maladaptive thoughts and behavior patterns | 24 mins | Goals setting |
|  |  |  |  | Activities record |
|  |  |  |  | Automatic thoughts record |
| 3 | Behavioral activation and identifying your thinking traps | Learning how to schedule activities and increase level of activity reasonably; introduction of common thinking traps of depression | 33 mins | Activities treasure chest |
|  |  |  |  | Activities scheduling |
|  |  |  |  | Activities record |
|  |  |  |  | Automatic thoughts record |
| 4 | Procrastination and thought challenging | Task decomposition to change avoidance behavior and procrastination; adjusting and challenging negative automatic thoughts with "thought-monitor table" | 25 mins | Task decomposition |
|  |  |  |  | Activities scheduling |
|  |  |  |  | Activities record |
|  |  |  |  | Automatic thoughts record |
| 5 | Attribution training and problem solving | Strategies about rational attribution and problem solving | 26 mins | Attribution training |
|  |  |  |  | Activities scheduling |
|  |  |  |  | Activities record |
|  |  |  |  | Problem solving |
| 6 | Realistic thinking | Identifying and evaluating your inner conviction; making more adaptive response to yourself, others and the world | 23 mins | Activities scheduling |
|  |  |  |  | Activities record |
|  |  |  |  | Identifying inner conviction |
|  |  |  |  | Evaluating inner conviction |
| 7 | Maintaining the effect and relapse prevention | Summaries and reviews about challenging depression; strategies about relapse prevention | 20 mins | New goals |

^a^CBT: cognitive behavioral therapy.


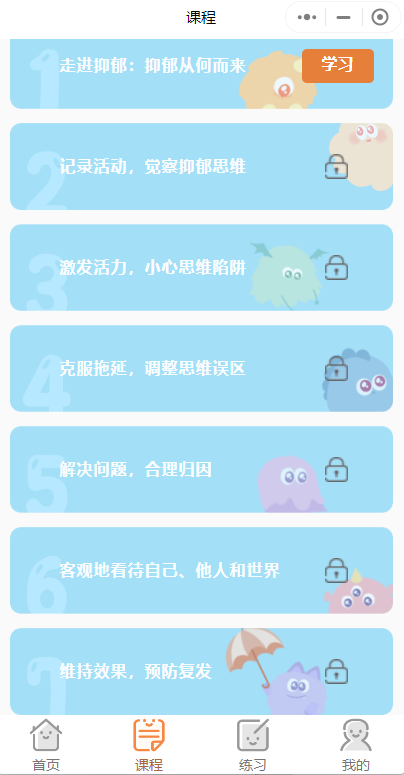


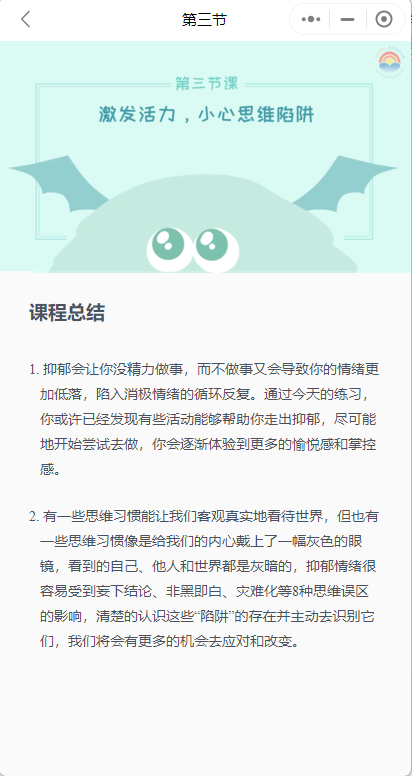

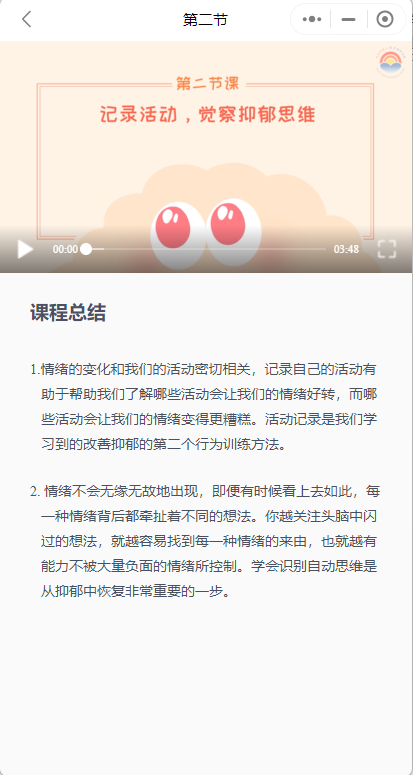

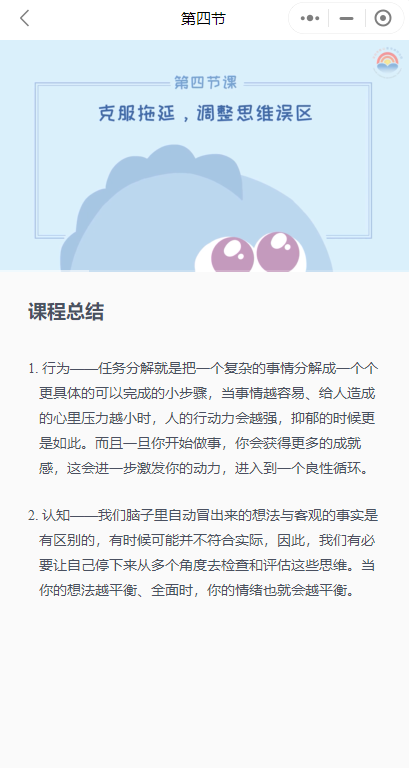

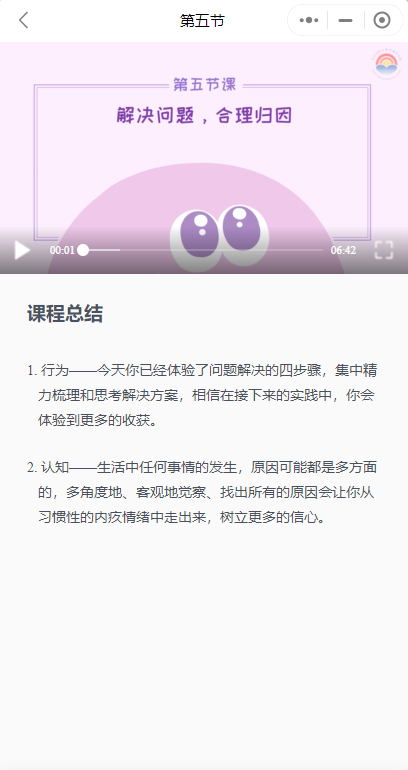

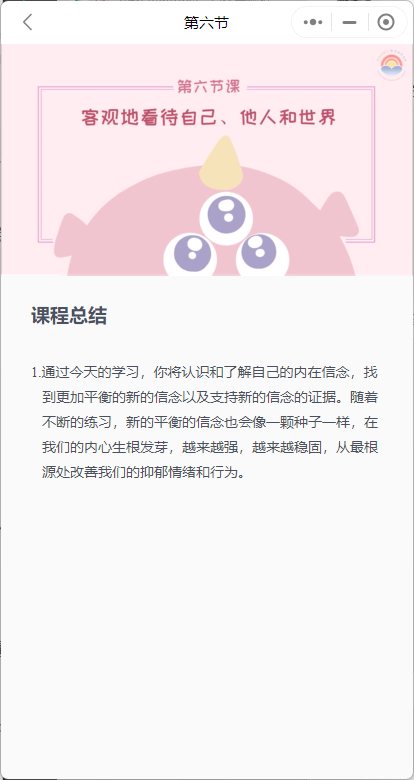

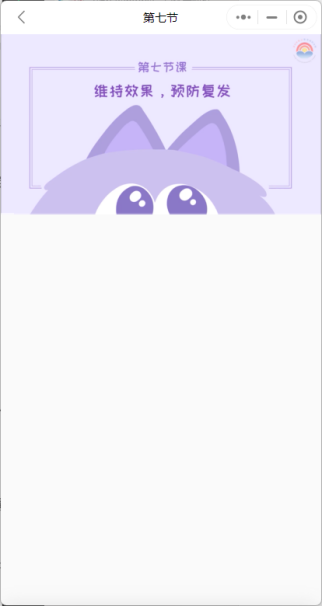

Supplement: Multimedia Appendix 1 [file mhealth-v14-e68394-s001.docx]
