# Supplementary material for: Long-Term Effectiveness of Unguided Internet-Based Cognitive Behavioral Therapy on Major Depressive Disorder in Chinese Adults: Randomized Controlled Trial With a 12-Month Follow-Up
Source: JMIR Mhealth Uhealth. 2026 Jun 24;14:e68394. doi: 10.2196/68394 (PMC13293601; doi:10.2196/68394)
Supplement: Multimedia Appendix 2 [file mhealth-v14-e68394-s002.docx]

**Criteria for defining treatment completion and participant attrition**

Before the start of the study, we specified criteria for defining treatment completion and participant attrition. The treatment completion of ICBT was defined as completing all therapy content (7 lessons in ICBT). Dropout criteria were operationally defined as follows:

For participants in the ICBT groups (Group I and II), dropout was determined if either:

1. The participant formally expressed intention to withdraw from the study at intervention or follow-up, or
2. Failed to participate or respond to contact attempts for six consecutive weeks or longer, despite at least weekly attempts at contacting.

For the WLC group, the dropout criterion was failure to complete the post-treatment assessment within the designated data collection window (one month).

The treatment completion rate was identified as the proportion of participants who met predefined criteria for treatment completion among those who started the course. The attrition/dropout rate in the ICBT group (group I and II) was defined as the proportion of participants who were lost to follow-up at the 12-month assessment among those who started the course.
