# Supplementary material for: Long-Term Effectiveness of Unguided Internet-Based Cognitive Behavioral Therapy on Major Depressive Disorder in Chinese Adults: Randomized Controlled Trial With a 12-Month Follow-Up
Source: JMIR Mhealth Uhealth. 2026 Jun 24;14:e68394. doi: 10.2196/68394 (PMC13293601; doi:10.2196/68394)

| **Table S1. Baseline characteristics of the participants in the intention-to-treat sample** | | | | |
| --- | --- | --- | --- | --- |
| **Variable ^a^** | **Overall  (N=317)** | **ICBT  (N=159)** | **WLC  (N=158)** | ***P*-value ^b^** |
| Age, mean (SD), years | 28.54 (7.04) | 29.21 (6.66) | 27.85 (7.36) | 0.085 |
| Gender |  |  |  | 0.319 |
| Male | 81 (25.6) | 45 (28.3) | 36 (22.8) |  |
| Female | 236 (74.4) | 114 (71.7) | 122 (77.2) |  |
| Nationality |  |  |  | 0.754 |
| Han | 296 (93.4) | 149 (93.7) | 147 (93.0) |  |
| Others | 18 (5.7) | 8 (5.0) | 10 (6.3) |  |
| Not available | 3 (0.9) | 2 (1.3) | 1 (0.6) |  |
| Educational levels |  |  |  | 0.352 |
| High school or below | 57 (18.0) | 31 (19.5) | 26 (16.5) |  |
| Undergraduate | 217 (68.5) | 105 (66.0) | 112 (70.9) |  |
| Master’s degree or above | 41 (12.9) | 23 (14.5) | 18 (11.4) |  |
| Not available | 2 (0.6) | 0 (0.0) | 2 (1.3) |  |
| Employment status |  |  |  | 0.099 |
| Employed | 202 (63.7) | 110 (69.2) | 92 (58.2) |  |
| Unemployed | 111 (35.0) | 48 (30.2) | 63 (39.9) |  |
| Not available | 4 (1.3) | 1 (0.6) | 3 (1.9) |  |
| Marital status |  |  |  | 0.469 |
| Married | 88 (27.8) | 49 (30.8) | 39 (24.7) |  |
| Unmarried/divorced/widowed | 213 (67.2) | 102 (64.2) | 111 (70.3) |  |
| Not available | 16 (5.0) | 8 (5.0) | 8 (5.1) |  |
| Monthly household income |  |  |  | 0.096 |
| No fixed income | 38 (12.0) | 13 (8.2) | 25 (15.8) |  |
| Below 10,000 CNY | 100 (31.5) | 54 (34.0) | 46 (29.1) |  |
| 10,000–20,000 CNY | 79 (24.9) | 46 (28.9) | 33 (20.9) |  |
| Above 20,000 CNY | 79 (24.9) | 38 (23.9) | 41 (25.9) |  |
| Not available | 21 (6.6) | 8 (5.0) | 13 (8.2) |  |
| Exercise |  |  |  | 0.607 |
| Yes | 90 (28.4) | 45 (28.3) | 45 (28.5) |  |
| No | 226 (71.3) | 113 (71.1) | 113 (71.5) |  |
| Not available | 1 (0.3) | 1 (0.6) | 0 (0.0) |  |
| Current drinking status |  |  |  | 0.381 |
| Yes | 264 (83.3) | 136 (85.5) | 128 (81.0) |  |
| No | 52 (16.4) | 23 (14.5) | 29 (18.4) |  |
| Not available | 1 (0.3) | 0 (0.0) | 1 (0.6) |  |
| Current smoking status |  |  |  | 0.158 |
| Yes | 126 (39.7) | 58 (36.5) | 68 (43.0) |  |
| No | 189 (59.6) | 101 (63.5) | 88 (55.7) |  |
| Not available | 2 (0.6) | 0 (0.0) | 2 (1.3) |  |
| Living alone |  |  |  | 0.495 |
| Yes | 79 (24.9) | 44 (27.7) | 35 (22.2) |  |
| No | 220 (69.4) | 107 (67.3) | 113 (71.5) |  |
| Not available | 18 (5.7) | 8 (5.0) | 10 (6.3) |  |
| Antidepressant use |  |  |  | 0.340 |
| Yes | 143 (45.1) | 67 (42.1) | 76 (48.1) |  |
| No | 174 (54.9) | 92 (57.9) | 82 (51.9) |  |
| First episode |  |  |  | 0.777 |
| Yes | 186 (58.7) | 93 (58.5) | 93 (58.9) |  |
| No | 123 (38.8) | 61 (38.4) | 62 (39.2) |  |
| Not available | 8 (2.5) | 5 (3.1) | 3 (1.9) |  |
| Age of onset, mean (SD), years | 24.61 (7.37) | 25.28 (7.29) | 23.93 (7.41) | 0.109 |
| Comorbidity |  |  |  | 0.324 |
| Yes | 117 (36.9) | 57 (35.8) | 60 (38.0) |  |
| No | 198 (62.5) | 102 (64.2) | 96 (60.8) |  |
| Not available | 2 (0.6) | 0 (0.0) | 2 (1.3) |  |
| Number of SLE, mean (SD) | 1.86 (1.87) | 1.92 (1.87) | 1.81 (1.87) | 0.618 |
| CTQ | 49.18 (13.69) | 50.16 (13.72) | 48.21 (13.64) | 0.235 |
| Emotional abuse scores, mean (SD) | 10.65 (4.97) | 10.59 (5.07) | 10.70 (4.89) | 0.850 |
| Physical abuse scores, mean (SD) | 7.00 (3.19) | 6.97 (3.21) | 7.04 (3.18) | 0.840 |
| Sexual abuse scores, mean (SD) | 5.80 (1.94) | 5.88 (2.09) | 5.72 (1.78) | 0.478 |
| Emotional neglect scores, mean (SD) | 15.86 (5.22) | 16.27 (5.09) | 15.46 (5.32) | 0.190 |
| Physical neglect scores, mean (SD) | 10.01 (3.87) | 10.45 (4.33) | 9.59 (3.33) | 0.059 |
| SSI scores, mean (SD) | 63.46 (22.01) | 63.52 (20.32) | 63.41 (23.65) | 0.967 |
| CD-RISC scores, mean (SD) | 36.62 (15.17) | 37.00 (15.11) | 36.25 (15.27) | 0.676 |
| RRS scores, mean (SD) | 56.75 (10.72) | 56.83 (11.22) | 56.68 (10.25) | 0.906 |
| ISI scores, mean (SD) | 15.41 (6.58) | 15.16 (6.31) | 15.67 (6.87) | 0.500 |
| PHQ-9 scores, mean (SD) | 13.20 (4.77) | 13.61 (5.02) | 12.78 (4.47) | 0.120 |
| GAD-7 scores, mean (SD) | 10.36 (4.61) | 10.86 (4.81) | 9.85 (4.36) | 0.053 |
| K-10 scores, mean (SD) | 29.78 (7.41) | 30.35 (7.27) | 29.20 (7.52) | 0.168 |
| SDS scores, mean (SD) | 13.92 (6.85) | 15.57 (7.05) | 12.26 (6.23) | <0.001 |
| GSES scores, mean (SD) | 19.62 (5.76) | 19.62 (5.51) | 19.62 (6.01) | 0.995 |
| SF-6D scores, mean (SD) | 0.56 (0.20) | 0.54 (0.21) | 0.58 (0.20) | 0.133 |
| DSS scores, mean (SD) | 52.26 (9.07) | 52.95 (8.90) | 51.57 (9.22) | 0.180 |
| Personal DSS scores, mean (SD) | 22.87 (5.25) | 23.18 (5.24) | 22.57 (5.26) | 0.306 |
| Perceived DSS scores, mean (SD) | 29.38 (6.66) | 29.77 (6.39) | 29.00 (6.91) | 0.310 |
| ^a^ Unless otherwise indicated, data are expressed as No. (%) of participants.  ^b^ Baseline characteristics were compared between the two groups using two independent-sample *t*-tests for continuous variables and Chi-Square tests or Fisher exact probabilities for categorical variables.  Abbreviations: ICBT, Internet-Based Cognitive Behavioral Therapy; WLC, waiting-list control; N, number of participants; SD, standard deviation; CNY, Chinese Yuan; SLE, The Stressful Life Event; CTQ, Childhood Trauma Questionnaire; SSI, Somatic Symptom Inventory; CD-RISC, Connor-Davidson Resilience Scale; RRS, Ruminative Responses Scale; ISI, Insomnia Severity Index; PHQ-9, Patient Health Questionnaire-9; GAD-7, General Anxiety Disorder-7; K-10, Kessler Psychological Distress Scale-10; SDS, Sheehan Disability Scale; GSES, General Self-Efficacy Scale; SF-6D, Short Form 6-Dimension; DSS, Depression Stigma Scale. | | | | |

| **Table S2. Outcomes for the intention-to-treat sample at pre- and post-treatment time points by treatment groups** | | | | | | | | | | | | | | |
| --- | --- | --- | --- | --- | --- | --- | --- | --- | --- | --- | --- | --- | --- | --- |
| **Outcome** | **Pre-treatment** | | | | | | **Post-treatment** | | | | | | **Mean difference**  **(SE) ^b^** | ***P*-value** |
|  | **ICBT** | | | **WLC** | | | **ICBT** | | | **WLC** | | |  |  |
|  | **N** | **Observed**  **mean**  **(SD)** | **Estimated**  **mean**  **(SE) ^a^** | **N** | **Observed**  **mean**  **(SD)** | **Estimated**  **mean**  **(SE) ^a^** | **N** | **Observed**  **mean**  **(SD)** | **Estimated**  **mean**  **(SE) ^a^** | **N** | **Observed**  **mean**  **(SD)** | **Estimated**  **mean**  **(SE) ^a^** |  |  |
| PHQ-9 scores | 159 | 13.61(5.02) | 13.61(0.41) | 158 | 12.78(4.47) | 12.78(0.41) | 159 | 8.87(5.70) | 8.87(0.45) | 158 | 11.05(5.37) | 11.06(0.44) | 3.01(0.65) | <0.001 |
| GAD-7 scores | 159 | 10.86(4.81) | 10.86(0.38) | 158 | 9.85(4.36) | 9.85(0.38) | 159 | 6.78(5.03) | 6.78(0.43) | 158 | 8.29(4.75) | 8.29(0.39) | 2.51(0.58) | <0.001 |
| K-10 scores | 159 | 30.35(7.27) | 30.35(0.67) | 158 | 29.20(7.52) | 29.20(0.67) | 159 | 24.00(9.60) | 24.00(0.73) | 158 | 26.63(8.97) | 26.63(0.7) | 3.78(0.97) | <0.001 |
| SDS scores | 159 | 15.57(7.05) | 15.99(0.57) | 158 | 12.26(6.23) | 12.69(0.58) | 159 | 11.46(7.18) | 11.88(0.64) | 158 | 10.78(6.84) | 11.22(0.61) | 2.64(0.74) | <0.001 |
| GSES scores | 159 | 19.62(5.51) | 19.70(0.49) | 158 | 19.62(6.01) | 19.97(0.50) | 159 | 21.57(5.96) | 21.66(0.62) | 158 | 20.53(6.18) | 20.88(0.52) | -1.05(1.07) | 0.330 |
| SF-6D scores | 159 | 0.54(0.21) | 0.54(0.02) | 158 | 0.58(0.20) | 0.58(0.02) | 159 | 0.61(0.22) | 0.61(0.02) | 158 | 0.62(0.19) | 0.62(0.02) | -0.02(0.02) | 0.418 |
| DSS scores | 159 | 52.95(8.90) | 52.95(0.74) | 158 | 51.57(9.22) | 51.57(0.74) | 159 | 53.22(10.19) | 53.22(0.92) | 158 | 54.17(8.77) | 54.17(0.76) | 2.32(1.42) | 0.108 |
| Personal DSS scores | 159 | 23.18(5.24) | 23.38(0.45) | 158 | 22.57(5.26) | 23.02(0.46) | 159 | 22.48(5.86) | 22.68(0.61) | 158 | 23.16(5.53) | 23.61(0.47) | 1.29(0.82) | 0.124 |
| Perceived DSS scores | 159 | 29.77(6.39) | 29.77(0.56) | 158 | 29.00(6.91) | 29.00(0.55) | 159 | 30.74(7.26) | 30.74(0.69) | 158 | 31.01(7.07) | 31.01(0.56) | 1.04(1.06) | 0.332 |
| ^a^ Estimated means are based on linear mixed models. The linear mixed models took the WLC group as the reference group.  ^b^ The differences posttreatment to baseline between the two treatment groups are based on the difference of least square means of linear mixed models.  Abbreviations: ICBT, Internet-Based Cognitive Behavioral Therapy; WLC, waiting-list control; N, number of participants; SD, standard deviation; SE, standard error; PHQ-9, Patient Health Questionnaire-9; GAD-7, General Anxiety Disorder-7; K-10, Kessler Psychological Distress Scale-10; SDS, Sheehan Disability Scale; GSES, General Self-Efficacy Scale; SF-6D, Short Form 6-Dimension; DSS, Depression Stigma Scale. | | | | | | | | | | | | | | |

| **Table S3. Outcome measures linear mixed model fixed effect estimates at 8 weeks in the intention-to-treat sample** | | | | | |
| --- | --- | --- | --- | --- | --- |
| **Outcome** | **Effect** | ***b*** | ***SE*** | **95% *CI*** | ***P*-value ^a^** |
| PHQ-9 | Treatment Group | -3.84 | 1.05 | (-5.92, -1.77) | <0.001 |
|  | Time | -4.74 | 0.45 | (-5.63, -3.84) | <0.001 |
|  | Time*Treatment Group | 3.01 | 0.65 | (1.71, 4.31) | <0.001 |
| GAD-7 | Treatment Group | -3.51 | 0.95 | (-5.38, -1.65) | <0.001 |
|  | Time | -4.07 | 0.43 | (-4.94, -3.21) | <0.001 |
|  | Time*Treatment Group | 2.51 | 0.58 | (1.37, 3.65) | <0.001 |
| K-10 | Treatment Group | -4.93 | 1.63 | (-8.13, -1.72) | <0.001 |
|  | Time | -6.35 | 0.70 | (-7.76, -4.95) | <0.001 |
|  | Time*Treatment Group | 3.78 | 0.97 | (1.85, 5.71) | <0.001 |
| SDS | Age | 0.06 | 0.05 | (-0.04, 0.16) | 0.240 |
|  | Gender | -1.28 | 0.83 | (-2.91, 0.36) | 0.120 |
|  | Antidepressants | -2.29 | 0.69 | (-3.65, -0.92) | <0.001 |
|  | Treatment Group | -5.93 | 1.26 | (-8.41, -3.45) | <0.001 |
|  | Time | -4.11 | 0.55 | (-5.22, -3.01) | <0.001 |
|  | Time*Treatment Group | 2.64 | 0.74 | (1.18, 4.10) | <0.001 |
| GSES | Age | 0.15 | 0.04 | (0.07, 0.24) | <0.001 |
|  | Gender | -0.92 | 0.70 | (-2.29, 0.46) | 0.190 |
|  | Antidepressants | 0.10 | 0.62 | (-1.11, 1.32) | 0.870 |
|  | Treatment Group | 1.31 | 0.97 | (-0.61, 3.24) | 0.180 |
|  | Time | 1.96 | 0.52 | (0.84, 3.08) | <0.001 |
|  | Time*Treatment Group | -1.05 | 1.07 | (-3.15, 1.06) | 0.330 |
| SF-6D | Treatment Group | 0.05 | 0.04 | (-0.02, 0.12) | 0.160 |
|  | Time | 0.06 | 0.01 | (0.03, 0.09) | <0.001 |
|  | Time*Treatment Group | -0.02 | 0.02 | (-0.06, 0.02) | 0.418 |
| DSS | Treatment Group | -3.70 | 2.19 | (-8.02, 0.62) | 0.090 |
|  | Time | 0.28 | 1.09 | (-1.96, 2.51) | 0.800 |
|  | Time*Treatment Group | 2.32 | 1.42 | (-0.53, 5.17) | 0.108 |
| Personal DSS | Age | 0.11 | 0.04 | (0.03, 0.18) | 0.010 |
|  | Gender | -1.39 | 0.61 | (-2.59, -0.19) | 0.020 |
|  | Antidepressants | 0.33 | 1.00 | (-1.64, 2.29) | 0.740 |
|  | Treatment Group | -1.66 | 1.24 | (-4.13, 0.81) | 0.190 |
|  | Time | -0.70 | 0.66 | (-2.09, 0.69) | 0.300 |
|  | Time*Treatment Group | 1.29 | 0.82 | (-0.37, 2.96) | 0.124 |
| Perceived DSS | Treatment Group | -1.81 | 1.65 | (-5.07, 1.46) | 0.280 |
|  | Time | 0.97 | 0.83 | (-0.75, 2.70) | 0.250 |
|  | Time*Treatment Group | 1.04 | 1.06 | (-1.09, 3.16) | 0.332 |
| The linear mixed models took the WLC group as the reference group. Abbreviations: SE, standard error; CI, confidence intervals; PHQ-9, Patient Health Questionnaire-9; GAD-7, General Anxiety Disorder-7; K-10, Kessler Psychological Distress Scale-10; SDS, Sheehan Disability Scale; GSES, General Self-Efficacy Scale; SF-6D, Short Form 6-Dimension; DSS, Depression Stigma Scale. | | | | | |

| **Table S4. Effect size estimates for changes in outcome measures at 8 weeks in the intention-to-treat sample** | | | | | | |
| --- | --- | --- | --- | --- | --- | --- |
| **Outcome** | **Within-Group (ICBT)** | | **Within-Group (WLC)** | | **Between-Group (ICBT *vs* WLC)** | |
|  | **Effect size** | **95% *CI*** | **Effect size** | **95% *CI*** | **Effect size** | **95% *CI*** |
| PHQ-9 | 0.88 | (0.78, 0.99) | 0.35 | (0.25, 0.45) | 0.39 | (0.29, 0.49) |
| GAD-7 | 0.83 | (0.73, 0.93) | 0.34 | (0.24, 0.44) | 0.31 | (0.21, 0.41) |
| K-10 | 0.75 | (0.65, 0.85) | 0.31 | (0.21, 0.41) | 0.28 | (0.18, 0.38) |
| SDS | 0.58 | (0.48, 0.68) | 0.23 | (0.13, 0.32) | -0.10 | (-0.19, 0.00) |
| GSES | -0.34 | (-0.44, -0.24) | -0.15 | (-0.25, -0.05) | -0.17 | (-0.27, -0.07) |
| SF-6D | -0.30 | (-0.39, -0.20) | -0.24 | (-0.34, -0.14) | 0.09 | (-0.01, 0.18) |
| DSS | -0.03 | (-0.13, 0.07) | -0.29 | (-0.39, -0.19) | 0.10 | (0.00, 0.20) |
| Personal DSS | 0.13 | (0.03, 0.22) | -0.11 | (-0.21, -0.01) | 0.12 | (0.02, 0.22) |
| Perceived DSS | -0.14 | (-0.24, -0.04) | -0.29 | (-0.39, -0.19) | 0.04 | (-0.06, 0.14) |
| Abbreviations: ICBT, Internet-Based Cognitive Behavioral Therapy; WLC, waiting-list control; Vs, versus; CI, confidence intervals; PHQ-9, Patient Health Questionnaire-9; GAD-7, General Anxiety Disorder-7; K-10, Kessler Psychological Distress Scale-10; SDS, Sheehan Disability Scale; GSES, General Self-Efficacy Scale; SF-6D, Short Form 6-Dimension; DSS, Depression Stigma Scale. | | | | | | |

**Figure S1. Estimated means and 95% confidence intervals (CIs) for time by treatment group interaction effects at 8 weeks in the intention-to-treat sample.** Linear mixed models with random intercept, including treatment groups (i.e., ICBT and WLC), time points of measurement (i.e., pre-treatment and post-treatment), and the treatment by time point interaction as fixed effects, are applied to calculate estimated means, mean differences in pre - and post-treatment changes between the two groups and their *P*-values. Patient-specific effects enter the model as a random effect with normal distribution and an expected value of 0.

Abbreviations: PHQ-9, Patient Health Questionnaire-9; GAD-7, General Anxiety Disorder-7; K-10, Kessler Psychological Distress Scale-10; SDS, Sheehan Disability Scale; GSES, General Self-Efficacy Scale; SF-6D, Short Form 6-Dimension; DSS, Depression Stigma Scale.


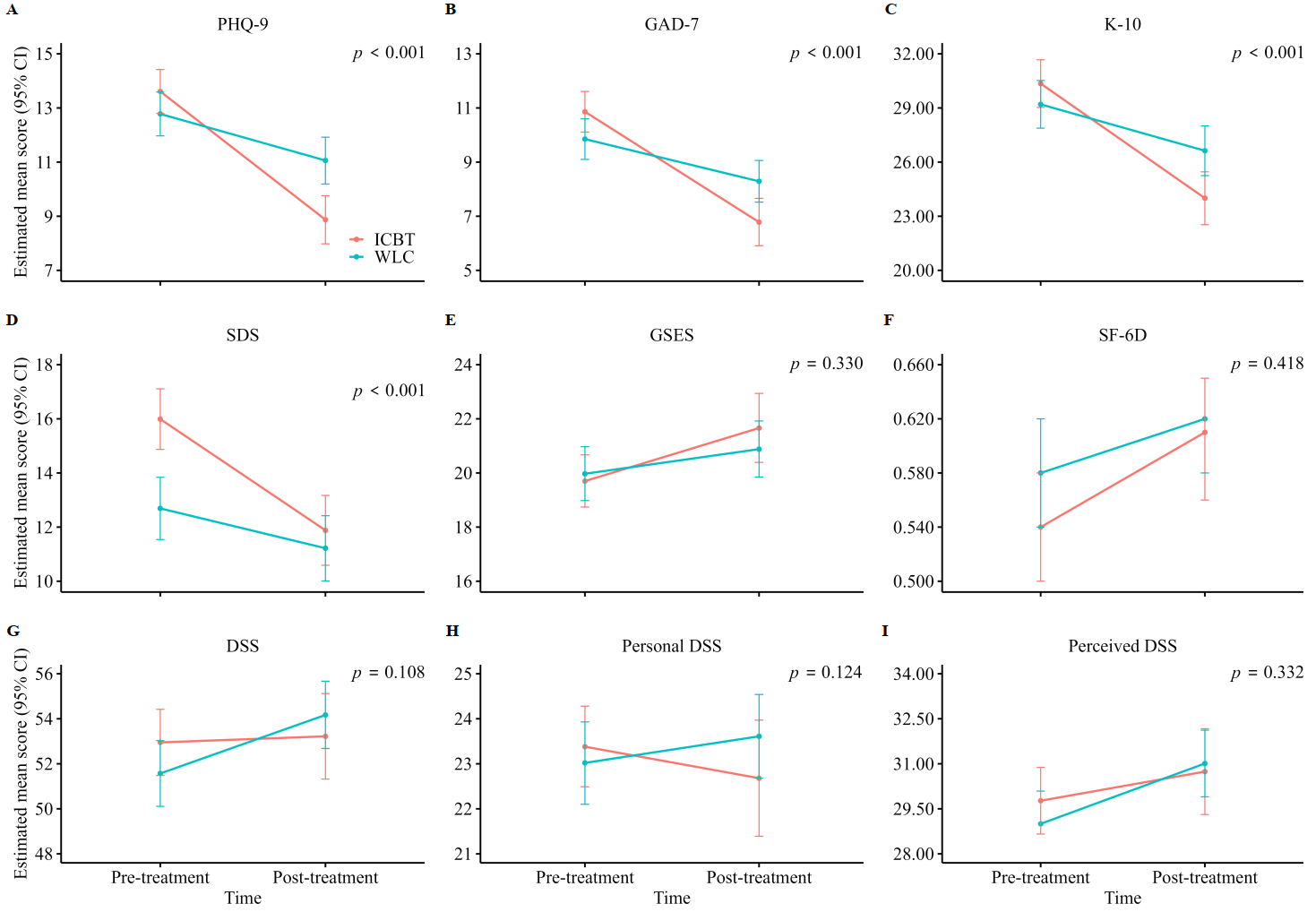

Supplement: Multimedia Appendix 3 [file mhealth-v14-e68394-s003.docx]
