# Supplementary material for: Long-Term Effectiveness of Unguided Internet-Based Cognitive Behavioral Therapy on Major Depressive Disorder in Chinese Adults: Randomized Controlled Trial With a 12-Month Follow-Up
Source: JMIR Mhealth Uhealth. 2026 Jun 24;14:e68394. doi: 10.2196/68394 (PMC13293601; doi:10.2196/68394)

| **Table S1. Baseline characteristics of the participants in the per-protocol set** | | | | |
| --- | --- | --- | --- | --- |
| **Variable ^a^** | **Overall  (N=266)** | **ICBT  (N=118)** | **WLC  (N=148)** | ***P*-value ^b^** |
| Age, mean (SD), years | 28.48 (7.10) | 29.19 (6.66) | 27.92 (7.41) | 0.146 |
| Gender |  |  |  | 0.727 |
| Male | 66 (24.8) | 31 (26.3) | 35 (23.6) |  |
| Female | 200 (75.2) | 87 (73.7) | 113 (76.4) |  |
| Nationality |  |  |  | 0.396 |
| Han | 253 (95.1) | 113 (95.8) | 140 (94.6) |  |
| Others | 12 (4.5) | 4 (3.4) | 8 (5.4) |  |
| Not available | 1 (0.4) | 1 (0.8) | 0 (0.0) |  |
| Educational levels |  |  |  | 0.492 |
| High school or below | 42 (15.8) | 18 (15.3) | 24 (16.2) |  |
| Undergraduate | 184 (69.2) | 79 (66.9) | 105 (70.9) |  |
| Master’s degree or above | 39 (14.7) | 21 (17.8) | 18 (12.2) |  |
| Not available | 1 (0.4) | 0 (0.0) | 1 (0.7) |  |
| Employment status |  |  |  | 0.087 |
| Employed | 170 (63.9) | 84 (71.2) | 86 (58.1) |  |
| Unemployed | 93 (35.0) | 33 (28.0) | 60 (40.5) |  |
| Not available | 3 (1.1) | 1 (0.8) | 2 (1.4) |  |
| Marital status |  |  |  | 0.426 |
| Married | 75 (28.2) | 37 (31.4) | 38 (25.7) |  |
| Unmarried/divorced/widowed | 181 (68.0) | 78 (66.1) | 103 (69.6) |  |
| Not available | 10 (3.8) | 3 (2.5) | 7 (4.7) |  |
| Monthly household income |  |  |  | 0.034 |
| No fixed income | 31 (11.7) | 8 (6.8) | 23 (15.5) |  |
| Below 10,000 CNY | 85 (32.0) | 41 (34.7) | 44 (29.7) |  |
| 10,000–20,000 CNY | 72 (27.1) | 40 (33.9) | 32 (21.6) |  |
| Above 20,000 CNY | 62 (23.3) | 24 (20.3) | 38 (25.7) |  |
| Not available | 16 (6.0) | 5 (4.2) | 11 (7.4) |  |
| Exercise |  |  |  | 0.757 |
| Yes | 73 (27.4) | 34 (28.8) | 39 (26.4) |  |
| No | 193 (72.6) | 84 (71.2) | 109 (73.6) |  |
| Current drinking status |  |  |  | 0.608 |
| Yes | 217 (81.6) | 98 (83.1) | 119 (80.4) |  |
| No | 48 (18.0) | 20 (16.9) | 28 (18.9) |  |
| Not available | 1 (0.4) | 0 (0.0) | 1 (0.7) |  |
| Current smoking status |  |  |  | 0.189 |
| Yes | 97 (36.5) | 37 (31.4) | 60 (40.5) |  |
| No | 168 (63.2) | 81 (68.6) | 87 (58.8) |  |
| Not available | 1 (0.4) | 0 (0.0) | 1 (0.7) |  |
| Living alone |  |  |  | 0.437 |
| Yes | 63 (23.7) | 32 (27.1) | 31 (20.9) |  |
| No | 189 (71.1) | 81 (68.6) | 108 (73.0) |  |
| Not available | 14 (5.3) | 5 (4.2) | 9 (6.1) |  |
| Antidepressant use |  |  |  | 0.370 |
| Yes | 122 (45.9) | 50 (42.4) | 72 (48.6) |  |
| No | 144 (54.1) | 68 (57.6) | 76 (51.4) |  |
| First episode |  |  |  | 0.554 |
| Yes | 155 (58.3) | 69 (58.5) | 86 (58.1) |  |
| No | 103 (38.7) | 44 (37.3) | 59 (39.9) |  |
| Not available | 8 (3.0) | 5 (4.2) | 3 (2.0) |  |
| Age of onset, mean (SD), years | 24.48 (7.43) | 25.16 (7.29) | 23.94 (7.53) | 0.196 |
| Comorbidity |  |  |  | 0.448 |
| Yes | 101 (38.0) | 45 (38.1) | 56 (37.8) |  |
| No | 163 (61.3) | 73 (61.9) | 90 (60.8) |  |
| Not available | 2 (0.8) | 0 (0.0) | 2 (1.4) |  |
| Number of SLE, mean (SD) | 1.90 (1.92) | 2.05 (2.00) | 1.79 (1.85) | 0.295 |
| CTQ | 49.14 (13.66) | 50.65 (13.72) | 47.98 (13.55) | 0.143 |
| Emotional abuse scores, mean (SD) | 10.76 (5.00) | 10.92 (5.24) | 10.64 (4.82) | 0.660 |
| Physical abuse scores, mean (SD) | 6.98 (3.15) | 7.06 (3.27) | 6.93 (3.06) | 0.750 |
| Sexual abuse scores, mean (SD) | 5.80 (1.91) | 5.91 (2.11) | 5.72 (1.75) | 0.428 |
| Emotional neglect scores, mean (SD) | 15.93 (5.25) | 16.57 (5.03) | 15.45 (5.38) | 0.104 |
| Physical neglect scores, mean (SD) | 9.94 (3.87) | 10.43 (4.44) | 9.58 (3.38) | 0.097 |
| SSI scores, mean (SD) | 62.92 (21.72) | 62.51 (19.06) | 63.24 (23.62) | 0.801 |
| CD-RISC scores, mean (SD) | 36.51 (14.80) | 36.38 (14.34) | 36.62 (15.19) | 0.902 |
| RRS scores, mean (SD) | 56.78 (10.73) | 56.75 (11.15) | 53.83 (10.03) | 0.975 |
| ISI scores, mean (SD) | 15.12 (6.68) | 14.58 (6.34) | 15.57 (6.93) | 0.237 |
| PHQ-9 scores, mean (SD) | 13.17 (4.78) | 13.53 (5.14) | 12.87 (4.47) | 0.263 |
| GAD-7 scores, mean (SD) | 10.26 (4.54) | 10.75 (4.65) | 9.86 (4.42) | 0.116 |
| K-10 scores, mean (SD) | 29.59 (7.49) | 30.11 (7.35) | 29.18 (7.59) | 0.313 |
| SDS scores, mean (SD) | 13.50 (6.83) | 14.88 (7.30) | 12.40 (6.23) | 0.003 |
| GSES scores, mean (SD) | 19.49 (5.85) | 19.22 (5.56) | 19.70 (6.08) | 0.505 |
| SF-6D scores, mean (SD) | 0.56 (0.21) | 0.54 (0.22) | 0.57 (0.20) | 0.214 |
| DSS scores, mean (SD) | 52.33 (9.23) | 53.44 (9.08) | 51.46 (9.28) | 0.086 |
| Personal DSS scores, mean (SD) | 22.72 (5.20) | 23.24 (5.07) | 22.33 (5.28) | 0.161 |
| Perceived DSS scores, mean (SD) | 29.60 (6.83) | 30.20 (6.61) | 29.14 (6.97) | 0.212 |
| ^a^ Unless otherwise indicated, data are expressed as No. (%) of participants.  ^b^ Baseline characteristics were compared between the two groups using two independent-sample *t*-tests for continuous variables and Chi-Square tests or Fisher exact probabilities for categorical variables.  Abbreviations: ICBT, Internet-Based Cognitive Behavioral Therapy; WLC, waiting-list control; N, number of participants; SD, standard deviation; CNY, Chinese Yuan; SLE, The Stressful Life Event; CTQ, Childhood Trauma Questionnaire; SSI, Somatic Symptom Inventory; CD-RISC, Connor-Davidson Resilience Scale; RRS, Ruminative Responses Scale; ISI, Insomnia Severity Index; PHQ-9, Patient Health Questionnaire-9; GAD-7, General Anxiety Disorder-7; K-10, Kessler Psychological Distress Scale-10; SDS, Sheehan Disability Scale; GSES, General Self-Efficacy Scale; SF-6D, Short Form 6-Dimension; DSS, Depression Stigma Scale. | | | | |

| **Table S2. Outcomes for the per-protocol set at pre- and post-treatment time points by treatment groups** | | | | | | | | | | | | | | |
| --- | --- | --- | --- | --- | --- | --- | --- | --- | --- | --- | --- | --- | --- | --- |
| **Outcome** | **Pre-treatment** | | | | | | **Post-treatment** | | | | | | **Mean difference**  **(SE) ^b^** | ***P*-value** |
|  | **ICBT** | | | **WLC** | | | **ICBT** | | | **WLC** | | |  |  |
|  | **N** | **Observed**  **mean**  **(SD)** | **Estimated**  **mean**  **(SE) ^a^** | **N** | **Observed**  **mean**  **(SD)** | **Estimated**  **mean**  **(SE) ^a^** | **N** | **Observed**  **mean**  **(SD)** | **Estimated**  **mean**  **(SE) ^a^** | **N** | **Observed**  **mean**  **(SD)** | **Estimated**  **mean**  **(SE) ^a^** |  |  |
| PHQ-9 scores | 118 | 13.53 (5.14) | 13.53(0.48) | 148 | 12.87 (4.47) | 12.87(0.43) | 118 | 8.48 (5.75) | 8.48(0.48) | 148 | 11.18 (5.37) | 11.18(0.43) | 3.36(0.65) | <0.001 |
| GAD-7 scores | 118 | 10.75 (4.65) | 10.75(0.43) | 148 | 9.86 (4.42) | 9.86(0.39) | 118 | 6.36 (4.96) | 6.36(0.43) | 148 | 8.42 (4.78) | 8.42(0.39) | 2.94(0.57) | <0.001 |
| K-10 scores | 118 | 30.11 (7.35) | 30.11(0.78) | 148 | 29.18 (7.59) | 29.18(0.7) | 118 | 23.00 (9.75) | 23.00(0.78) | 148 | 26.80 (8.99) | 26.80(0.7) | 4.73(0.98) | <0.001 |
| SDS scores | 118 | 14.88 (7.30) | 15.34(0.65) | 148 | 12.40 (6.23) | 12.80(0.6) | 116 | 21.52 (5.92) | 11.64(0.66) | 148 | 20.52 (6.22) | 14.34(0.6) | 5.23(0.73) | <0.001 |
| GSES scores | 118 | 19.22 (5.56) | 19.33(0.58) | 148 | 19.70 (6.08) | 20.02(0.53) | 113 | 0.60 (0.23) | 21.59(0.58) | 148 | 0.62 (0.20) | 20.84(0.53) | -1.44(0.57) | 0.012 |
| SF-6D scores | 118 | 0.54 (0.22) | 0.54(0.02) | 148 | 0.57 (0.20) | 0.57(0.02) | 113 | 11.26 (7.22) | 0.60(0.02) | 148 | 13.94 (6.87) | 0.62(0.02) | -0.01(0.02) | 0.567 |
| DSS scores | 118 | 53.44 (9.08) | 53.45(0.87) | 148 | 51.46 (9.28) | 51.46(0.77) | 117 | 52.76 (10.25) | 52.74(0.86) | 148 | 54.07 (8.89) | 54.07(0.77) | 3.32(1.36) | 0.015 |
| Personal DSS scores | 118 | 23.24 (5.07) | 23.57(0.52) | 148 | 22.33 (5.28) | 22.78(0.47) | 117 | 22.26 (5.64) | 22.50(0.52) | 148 | 23.15 (5.61) | 23.62(0.47) | 1.90(0.69) | 0.006 |
| Perceived DSS scores | 118 | 30.20 (6.61) | 30.17(0.65) | 148 | 29.14 (6.97) | 29.15(0.58) | 117 | 30.50 (7.18) | 30.49(0.65) | 148 | 30.93 (7.18) | 30.93(0.58) | 1.46(0.98) | 0.139 |
| ^a^ Estimated means are based on linear mixed models. The linear mixed models took the WLC group as the reference group.  ^b^ The differences posttreatment to baseline between the two treatment groups are based on the difference of least square means of linear mixed models.  Abbreviations: ICBT, Internet-Based Cognitive Behavioral Therapy; WLC, waiting-list control; N, number of participants; SD, standard deviation; SE, standard error; PHQ-9, Patient Health Questionnaire-9; GAD-7, General Anxiety Disorder-7; K-10, Kessler Psychological Distress Scale-10; SDS, Sheehan Disability Scale; GSES, General Self-Efficacy Scale; SF-6D, Short Form 6-Dimension; DSS, Depression Stigma Scale. | | | | | | | | | | | | | | |

| **Table S3. Outcome measures linear mixed model fixed effect estimates at 8 weeks in the per-protocol set** | | | | | |
| --- | --- | --- | --- | --- | --- |
| **Outcome** | **Effect** | ***b*** | ***SE*** | **95% *CI*** | ***P*-value ^a^** |
| PHQ-9 | Treatment Group | -4.02 | 1.12 | (-6.21, -1.84) | <0.001 |
|  | Time | -5.05 | 0.48 | (-6.00, -4.10) | <0.001 |
|  | Time*Treatment Group | 3.36 | 0.65 | (2.09, 4.63) | <0.001 |
| GAD-7 | Treatment Group | -3.82 | 1.00 | (-5.77, -1.86) | <0.001 |
|  | Time | -4.38 | 0.43 | (-5.22, -3.54) | <0.001 |
|  | Time*Treatment Group | 2.94 | 0.57 | (1.81, 4.06) | <0.001 |
| K-10 | Treatment Group | -5.67 | 1.73 | (-9.06, -2.27) | 0.001 |
|  | Time | -7.11 | 0.73 | (-8.54, -5.68) | <0.001 |
|  | Time*Treatment Group | 4.73 | 0.98 | (2.82, 6.65) | <0.001 |
| SDS | Age | 0.06 | 0.05 | (-0.04, 0.17) | 0.218 |
|  | Gender | -1.22 | 0.86 | (-2.91, 0.47) | 0.158 |
|  | Antidepressants | -2.75 | 0.75 | (-4.22, -1.28) | <0.001 |
|  | Treatment Group | -7.77 | 1.32 | (-10.37, -5.18) | <0.001 |
|  | Time | -3.69 | 0.54 | (-4.76, -2.63) | <0.001 |
|  | Time*Treatment Group | 5.23 | 0.73 | (3.81, 6.66) | <0.001 |
| GSES | Age | 0.12 | 0.05 | (0.03, 0.22) | 0.010 |
|  | Gender | -0.99 | 0.78 | (-2.52, 0.53) | 0.201 |
|  | Antidepressants | 0.48 | 0.67 | (-0.85, 1.80) | 0.481 |
|  | Treatment Group | 2.14 | 1.09 | (0.01, 4.27) | 0.050 |
|  | Time | 2.26 | 0.43 | (1.42, 3.10) | <0.001 |
|  | Time*Treatment Group | -1.44 | 0.57 | (-2.56, -0.32) | 0.012 |
| SF-6D | Treatment Group | 0.04 | 0.04 | (-0.03, 0.12) | 0.269 |
|  | Time | 0.06 | 0.02 | (0.03, 0.09) | <0.001 |
|  | Time*Treatment Group | -0.01 | 0.02 | (-0.06, 0.03) | 0.567 |
| DSS | Treatment Group | -5.32 | 2.25 | (-9.72, -0.91) | 0.019 |
|  | Time | -0.71 | 1.02 | (-2.71, 1.28) | 0.484 |
|  | Time*Treatment Group | 3.32 | 1.36 | (0.67, 5.98) | 0.015 |
| Personal DSS | Age | 0.11 | 0.04 | (0.03, 0.19) | 0.005 |
|  | Gender | -1.60 | 0.65 | (-2.88, -0.33) | 0.015 |
|  | Antidepressants | 0.41 | 0.57 | (-0.70, 1.52) | 0.468 |
|  | Treatment Group | -2.69 | 1.18 | (-5.00, -0.38) | 0.023 |
|  | Time | -1.07 | 0.51 | (-2.07, -0.06) | 0.039 |
|  | Time*Treatment Group | 1.90 | 0.69 | (0.56, 3.25) | 0.006 |
| Perceived DSS | Treatment Group | -2.48 | 1.65 | (-5.71, 0.74) | 0.132 |
|  | Time | 0.32 | 0.74 | (-1.13, 1.76) | 0.667 |
|  | Time*Treatment Group | 1.46 | 0.98 | (-0.47, 3.39) | 0.139 |
| The linear mixed models took the WLC group as the reference group. Abbreviations: SE, standard error; CI, confidence intervals; PHQ-9, Patient Health Questionnaire-9; GAD-7, General Anxiety Disorder-7; K-10, Kessler Psychological Distress Scale-10; SDS, Sheehan Disability Scale; GSES, General Self-Efficacy Scale; SF-6D, Short Form 6-Dimension; DSS, Depression Stigma Scale. | | | | | |

| **Table S4. Effect size estimates for changes in outcome measures at 8 weeks in the per-protocol set** | | | | | | |
| --- | --- | --- | --- | --- | --- | --- |
| **Outcome** | **Within-Group (ICBT)** | | **Within-Group (WLC)** | | **Between-Group (ICBT *vs* WLC)** | |
|  | **Effect size** | **95% *CI*** | **Effect size** | **95% *CI*** | **Effect size** | **95% *CI*** |
| PHQ-9 | 0.93 | (0.66, 1.20) | 0.34 | (0.11, 0.57) | 0.49 | (0.24, 0.73) |
| GAD-7 | 0.91 | (0.64, 1.18) | 0.31 | (0.08, 0.54) | 0.42 | (0.18, 0.67) |
| K-10 | 0.82 | (0.56, 1.09) | 0.29 | (0.06, 0.52) | 0.41 | (0.16, 0.65) |
| SDS | 0.50 | (0.24, 0.76) | -0.23 | (-0.46, -0.01) | 0.38 | (0.14, 0.63) |
| GSES | -0.40 | (-0.66, -0.14) | -0.13 | (-0.36, 0.10) | -0.16 | (-0.41, 0.08) |
| SF-6D | -0.26 | (-0.52, 0.00) | -0.24 | (-0.47, -0.01) | 0.10 | (-0.15, 0.34) |
| DSS | 0.07 | (-0.19, 0.33) | -0.29 | (-0.52, -0.06) | 0.14 | (-0.11, 0.38) |
| Personal DSS | 0.18 | (-0.08, 0.44) | -0.15 | (-0.38, 0.08) | 0.16 | (-0.09, 0.40) |
| Perceived DSS | -0.04 | (-0.30, 0.22) | -0.25 | (-0.48, -0.02) | 0.06 | (-0.18, 0.30) |
| Abbreviations: ICBT, Internet-Based Cognitive Behavioral Therapy; WLC, waiting-list control; Vs, versus; CI, confidence intervals; PHQ-9, Patient Health Questionnaire-9; GAD-7, General Anxiety Disorder-7; K-10, Kessler Psychological Distress Scale-10; SDS, Sheehan Disability Scale; GSES, General Self-Efficacy Scale; SF-6D, Short Form 6-Dimension; DSS, Depression Stigma Scale. | | | | | | |

**Figure S1. Estimated means and 95% confidence intervals (CIs) for time by treatment group interaction effects at 8 weeks in the per-protocol set.** Linear mixed models with random intercept, including treatment groups (i.e., ICBT and WLC), time points of measurement (i.e., pre-treatment and post-treatment), and the treatment by time point interaction as fixed effects, are applied to calculate estimated means, mean differences in pre - and post-treatment changes between the two groups and their *P*-values. Patient-specific effects enter the model as a random effect with normal distribution and an expected value of 0.

Abbreviations: PHQ-9, Patient Health Questionnaire-9; GAD-7, General Anxiety Disorder-7; K-10, Kessler Psychological Distress Scale-10; SDS, Sheehan Disability Scale; GSES, General Self-Efficacy Scale; SF-6D, Short Form 6-Dimension; DSS, Depression Stigma Scale.


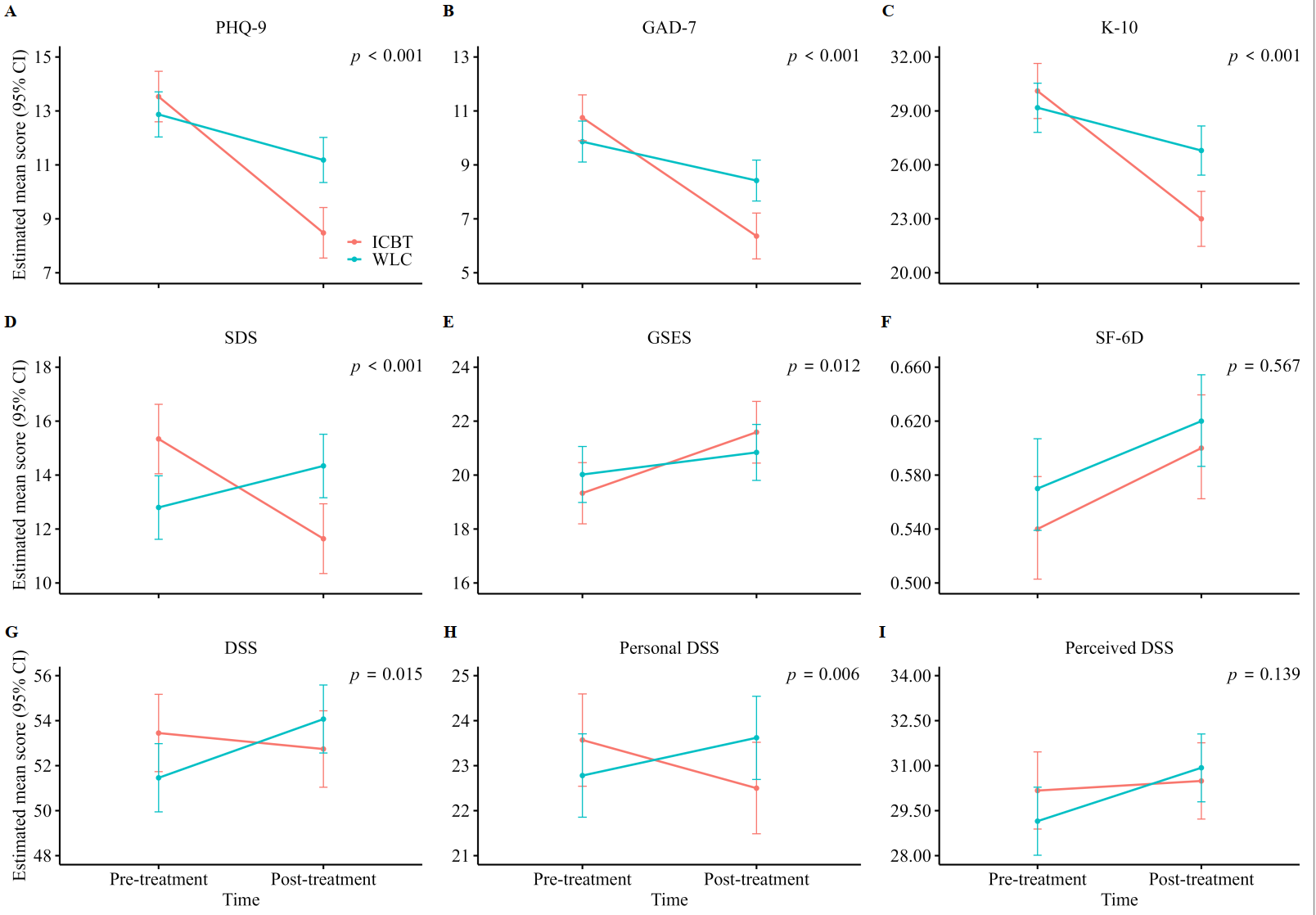

Supplement: Multimedia Appendix 4 [file mhealth-v14-e68394-s004.docx]
