# Supplementary material for: Long-Term Effectiveness of Unguided Internet-Based Cognitive Behavioral Therapy on Major Depressive Disorder in Chinese Adults: Randomized Controlled Trial With a 12-Month Follow-Up
Source: JMIR Mhealth Uhealth. 2026 Jun 24;14:e68394. doi: 10.2196/68394 (PMC13293601; doi:10.2196/68394)

| **Table S1.** Outcome measures linear mixed model fixed effect estimates at 8 weeks | | | | | |
| --- | --- | --- | --- | --- | --- |
| **Outcome** | **Effect** | ***b*** | ***SE*** | **95% *CI*** | ***P*-value ^a^** |
| PHQ-9 | Treatment Group | 1.04 | 0.60 | (-0.14, 2.22) | 0.081 |
|  | Time | -1.69 | 0.43 | (-2.53, -0.85) | <0.001*** |
|  | Time*Treatment Group | -3.65 | 0.63 | (-4.88, -2.42) | <0.001*** |
| GAD-7 | Treatment Group | 0.77 | 0.54 | (-0.29, 1.83) | 0.160 |
|  | Time | -1.45 | 0.38 | (-2.19, -0.71) | <0.001*** |
|  | Time*Treatment Group | -2.85 | 0.56 | (-3.95, -1.75) | <0.001*** |
| K-10 | Treatment Group | 1.10 | 0.97 | (-0.80, 3.00) | 0.256 |
|  | Time | -2.38 | 0.65 | (-3.65, -1.11) | <0.001*** |
|  | Time*Treatment Group | -4.87 | 0.96 | (-6.75, -2.99) | <0.001*** |
| SDS | Age | 0.05 | 0.05 | (-0.05, 0.15) | 0.305 |
|  | Gender | -1.23 | 0.81 | (-2.82, 0.36) | 0.131 |
|  | Antidepressants | -2.51 | 0.71 | (-3.90, -1.12) | <0.001*** |
|  | Treatment Group | 2.85 | 0.78 | (1.32, 4.38) | <0.001*** |
|  | Time | 1.54 | 0.49 | (0.58, 2.50) | 0.002** |
|  | Time*Treatment Group | -5.57 | 0.72 | (-6.98, -4.16) | <0.001*** |
| GSES | Age | 0.15 | 0.04 | (0.07, 0.23) | 0.001*** |
|  | Gender | -0.96 | 0.72 | (-2.37, 0.45) | 0.183 |
|  | Antidepressants | 0.10 | 0.62 | (-1.12, 1.32) | 0.874 |
|  | Treatment Group | -0.37 | 0.67 | (-1.68, 0.94) | 0.579 |
|  | Time | 0.82 | 0.37 | (0.09, 1.55) | 0.029* |
|  | Time*Treatment Group | 1.42 | 0.56 | (0.32, 2.52) | 0.011* |
| SF-6D | Treatment Group | -0.03 | 0.02 | (-0.07, 0.01) | 0.260 |
|  | Time | 0.05 | 0.01 | (0.03, 0.07) | 0.001*** |
|  | Time*Treatment Group | 0.01 | 0.02 | (-0.03, 0.05) | 0.497 |
| DSS | Treatment Group | 1.25 | 1.09 | (-0.89, 3.39) | 0.250 |
|  | Time | 2.61 | 0.90 | (0.85, 4.37) | 0.004** |
|  | Time*Treatment Group | -2.93 | 1.33 | (-5.54, -0.32) | 0.028* |
| Personal DSS | Age | 0.13 | 0.04 | (0.05, 0.21) | 0.001*** |
|  | Gender | -1.71 | 0.62 | (-2.93, -0.49) | 0.006** |
|  | Antidepressants | 0.35 | 0.54 | (-0.71, 1.41) | 0.522 |
|  | Treatment Group | 0.51 | 0.62 | (-0.71, 1.73) | 0.413 |
|  | Time | 0.84 | 0.46 | (-0.06, 1.74) | 0.069 |
|  | Time*Treatment Group | -1.80 | 0.68 | (-3.13, -0.47) | 0.008** |
| Perceived DSS | Treatment Group | 0.56 | 0.81 | (-1.03, 2.15) | 0.489 |
|  | Time | 1.78 | 0.65 | (0.51, 3.05) | 0.007** |
|  | Time*Treatment Group | -1.21 | 0.95 | (-3.07, 0.65) | 0.206 |
| ^a^ **p*<.05, ** *p*<.01, *** *p*<.001. The Linear Mixed Models took the ICBT intervention as the reference group.  Abbreviations: SE, standard error; CI, confidence intervals; PHQ-9, Patient Health Questionnaire-9; GAD-7, General Anxiety Disorder-7; K-10, Kessler Psychological Distress Scale-10; SDS, Sheehan Disability Scale; GSES, General Self-Efficacy Scale; SF-6D, Short Form 6-Dimension; DSS, Depression Stigma Scale. | | | | | |

| **Table S2.** Effect size estimates for changes in outcome measures at 8 weeks | | | | | | |
| --- | --- | --- | --- | --- | --- | --- |
| **Outcome** | **Within-Group (ICBT)** | | **Within-Group (WLC)** | | **Between-Group (ICBT *vs* WLC)** | |
|  | **Effect size** | **95% *CI*** | **Effect size** | **95% *CI*** | **Effect size** | **95% *CI*** |
| PHQ-9 | 1.02 | (0.76, 1.27) | 0.34 | (0.11, 0.57) | 0.50 | (0.26, 0.75) |
| GAD-7 | 0.89 | (0.63, 1.14) | 0.31 | (0.08, 0.54) | 0.43 | (0.18, 0.67) |
| K-10 | 0.87 | (0.61, 1.12) | 0.29 | (0.06, 0.52) | 0.41 | (0.17, 0.66) |
| SDS | 0.58 | (0.33, 0.83) | -0.23 | (-0.46, -0.01) | 0.41 | (0.17, 0.66) |
| GSES | -0.39 | (-0.63, -0.14) | -0.13 | (-0.36, 0.10) | -0.19 | (-0.44, 0.05) |
| SF-6D | -0.27 | (-0.51, -0.02) | -0.24 | (-0.47, -0.01) | 0.07 | (-0.17, 0.32) |
| DSS | 0.01 | (-0.23, 0.26) | -0.29 | (-0.52, -0.06) | 0.16 | (-0.09, 0.40) |
| Personal DSS | 0.15 | (-0.10, 0.39) | -0.15 | (-0.38, 0.08) | 0.17 | (-0.07, 0.41) |
| Perceived DSS | -0.10 | (-0.34, 0.15) | -0.25 | (-0.48, -0.02) | 0.07 | (-0.17, 0.32) |
| Abbreviations: ICBT, Internet-Based Cognitive Behavioral Therapy; WLC, waitlist control; Vs, versus; CI, confidence intervals; PHQ-9, Patient Health Questionnaire-9; GAD-7, General Anxiety Disorder-7; K-10, Kessler Psychological Distress Scale-10; SDS, Sheehan Disability Scale; GSES, General Self-Efficacy Scale; SF-6D, Short Form 6-Dimension; DSS, Depression Stigma Scale. | | | | | | |

| **Table S3. Paired comparisons of marginal fixed effects at different time points** | | | | |
| --- | --- | --- | --- | --- |
| **Measure** | **Effect** | ***b*** | **SE *b*** | ***P* value** |
| PHQ-9 | T0 - T1 | 4.71 | 0.30 | <0.001 |
|  | T0 - T2 | 4.10 | 0.31 | <0.001 |
|  | T0 - T3 | 3.73 | 0.31 | <0.001 |
|  | T0 - T4 | 3.90 | 0.32 | <0.001 |
|  | T1 - T2 | -0.61 | 0.32 | 0.69 |
|  | T1 - T3 | -0.98 | 0.32 | 0.08 |
|  | T1 - T4 | -0.81 | 0.33 | 0.33 |
|  | T2 - T3 | -0.37 | 0.32 | 0.99 |
|  | T2 - T4 | -0.20 | 0.33 | >0.99 |
|  | T3 - T4 | 0.17 | 0.34 | >0.99 |
| GAD-7 | T0 - T1 | 3.51 | 0.29 | <0.001 |
|  | T0 - T2 | 2.74 | 0.29 | <0.001 |
|  | T0 - T3 | 2.64 | 0.29 | <0.001 |
|  | T0 - T4 | 2.88 | 0.31 | <0.001 |
|  | T1 - T2 | -0.77 | 0.30 | 0.26 |
|  | T1 - T3 | -0.88 | 0.30 | 0.12 |
|  | T1 - T4 | -0.63 | 0.31 | 0.63 |
|  | T2 - T3 | -0.12 | 0.31 | >0.99 |
|  | T2 - T4 | 0.14 | 0.32 | >0.99 |
|  | T3 - T4 | 0.25 | 0.32 | >0.99 |
| K-10 | T0 - T1 | 5.95 | 0.51 | <0.001 |
|  | T0 - T2 | 5.48 | 0.51 | <0.001 |
|  | T0 - T3 | 4.69 | 0.52 | <0.001 |
|  | T0 - T4 | 4.84 | 0.54 | <0.001 |
|  | T1 - T2 | -0.47 | 0.53 | >0.99 |
|  | T1 - T3 | -1.26 | 0.53 | 0.39 |
|  | T1 - T4 | -1.11 | 0.55 | 0.64 |
|  | T2 - T3 | -0.79 | 0.54 | 0.93 |
|  | T2 - T4 | -0.64 | 0.56 | 0.99 |
|  | T3 - T4 | 0.15 | 0.56 | >0.99 |
| SDS | T0 - T1 | 3.72 | 0.43 | <0.001 |
|  | T0 - T2 | 4.34 | 0.43 | <0.001 |
|  | T0 - T3 | 4.62 | 0.44 | <0.001 |
|  | T0 - T4 | 4.60 | 0.46 | <0.001 |
|  | T1 - T2 | 0.62 | 0.45 | 0.64 |
|  | T1 - T3 | 0.90 | 0.45 | 0.27 |
|  | T1 - T4 | 0.88 | 0.47 | 0.33 |
|  | T2 - T3 | 0.28 | 0.45 | 0.97 |
|  | T2 - T4 | 0.26 | 0.47 | 0.98 |
|  | T3 - T4 | -0.02 | 0.47 | >0.99 |
| SF-6D | T0 - T1 | -0.04 | 0.01 | 0.02 |
|  | T0 - T2 | -0.06 | 0.01 | <0.001 |
|  | T0 - T3 | -0.08 | 0.01 | <0.001 |
|  | T0 - T4 | -0.08 | 0.01 | <0.001 |
|  | T1 - T2 | -0.02 | 0.01 | 0.71 |
|  | T1 - T3 | -0.04 | 0.01 | 0.01 |
|  | T1 - T4 | -0.04 | 0.01 | 0.04 |
|  | T2 - T3 | -0.03 | 0.01 | 0.30 |
|  | T2 - T4 | -0.02 | 0.01 | 0.53 |
|  | T3 - T4 | 0.004 | 0.01 | >0.99 |
| GSES | T0 - T1 | -1.54 | 0.37 | <0.001 |
|  | T0 - T2 | -1.45 | 0.37 | <0.001 |
|  | T0 - T3 | -1.53 | 0.37 | <0.001 |
|  | T0 - T4 | -1.42 | 0.39 | 0.002 |
|  | T1 - T2 | 0.09 | 0.38 | >0.99 |
|  | T1 - T3 | 0.004 | 0.40 | >0.99 |
|  | T1 - T4 | 0.12 | 0.38 | >0.99 |
|  | T2 - T3 | -0.08 | 0.40 | >0.99 |
|  | T2 - T4 | 0.03 | 0.40 | >0.99 |
|  | T3 - T4 | 0.11 | 0.40 | >0.99 |
| DSS | T0 - T1 | 1.09 | 0.63 | 0.42 |
|  | T0 - T2 | 0.11 | 0.64 | >0.99 |
|  | T0 - T3 | -0.13 | 0.65 | >0.99 |
|  | T0 - T4 | 0.60 | 0.68 | 0.90 |
|  | T1 - T2 | -0.98 | 0.66 | 0.56 |
|  | T1 - T3 | -1.22 | 0.66 | 0.35 |
|  | T1 - T4 | -0.49 | 0.69 | 0.95 |
|  | T2 - T3 | -0.23 | 0.67 | >0.99 |
|  | T2 - T4 | 0.49 | 0.69 | 0.95 |
|  | T3 - T4 | 0.73 | 0.70 | 0.84 |
| DSS Inner | T0 - T1 | 0.93 | 0.34 | 0.046 |
|  | T0 - T2 | 0.31 | 0.34 | 0.89 |
|  | T0 - T3 | 0.001 | 0.35 | >0.99 |
|  | T0 - T4 | -0.13 | 0.36 | >0.99 |
|  | T1 - T2 | -0.62 | 0.35 | 0.39 |
|  | T1 - T3 | -0.93 | 0.35 | 0.06 |
|  | T1 - T4 | -1.06 | 0.36 | 0.03 |
|  | T2 - T3 | -0.31 | 0.35 | 0.90 |
|  | T2 - T4 | -0.44 | 0.37 | 0.75 |
|  | T3 - T4 | -0.13 | 0.37 | >0.99 |
| DSS perceived | T0 - T1 | 0.18 | 0.44 | 0.99 |
|  | T0 - T2 | -0.17 | 0.44 | >0.99 |
|  | T0 - T3 | -0.10 | 0.45 | >0.99 |
|  | T0 - T4 | 0.76 | 0.47 | 0.48 |
|  | T1 - T2 | -0.35 | 0.45 | 0.94 |
|  | T1 - T3 | -0.27 | 0.46 | 0.98 |
|  | T1 - T4 | 0.58 | 0.47 | 0.74 |
|  | T2 - T3 | 0.08 | 0.46 | >0.99 |
|  | T2 - T4 | 0.93 | 0.48 | 0.29 |
|  | T3 - T4 | 0.85 | 0.48 | 0.39 |
| Abbreviations: SE, standard error; PHQ-9, Patient Health Questionnaire-9; GAD-7, General Anxiety Disorder-7; K-10, Kessler Psychological Distress Scale-10; SDS, Sheehan Disability Scale; GSES, General Self-Efficacy Scale; SF-6D, Short Form 6-Dimension; DSS, Depression Stigma Scale; T0, pre-treatment; T1, post-treatment; T2, 3-month follow up; T3, 6-month follow up; T4, 12-month follow up. | | | | |

| **Table S4.** Effect size estimates for changes in outcome measures at 8 weeks and 3-, 6-, and 12-month follow-up in participants receiving ICBT intervention in ICBT and waitlist control group | | | | | | |
| --- | --- | --- | --- | --- | --- | --- |
| **Outcomes** | **T2-T0** | **T3-T0** | **T4-T0** | **T2-T1** | **T3-T1** | **T4-T1** |
|  | **Effect size (95% *CI*)** | | | **Effect size (95% *CI*)** | | |
| PHQ-9 | 0.70 (0.55, 0.86) | 0.62 (0.46, 0.78) | 0.66 (0.49, 0.82) | -0.11 (-0.31, 0.08) | -0.18 (-0.37, 0.02) | -0.15 (-0.35, 0.05) |
| GAD-7 | 0.54 (0.38, 0.69) | 0.52 (0.37, 0.68) | 0.57 (0.41, 0.73) | -0.15 (-0.34, 0.04) | -0.16 (-0.36, 0.03) | -0.12 (-0.32, 0.08) |
| K-10 | 0.66 (0.51, 0.82) | 0.56 (0.41, 0.72) | 0.57 (0.41, 0.74) | -0.06 (-0.25, 0.14) | -0.14 (-0.34, 0.06) | -0.13 (-0.34, 0.07) |
| SDS | 0.67 (0.52, 0.82) | 0.71 (0.55, 0.86) | 0.73 (0.56, 0.89) | 0.09 (-0.10, 0.29) | 0.13 (-0.07, 0.33) | 0.15 (-0.06, 0.35) |
| GSES | -0.24 (-0.39, -0.09) | -0.27 (-0.42, -0.11) | -0.22 (-0.38, -0.06) | 0.04 (-0.16, 0.24) | 0.01 (-0.19, 0.21) | 0.05 (-0.15, 0.26) |
| SF-6D | -0.25 (-0.40, -0.10) | -0.39 (-0.54, -0.23) | -0.36 (-0.52, -0.19) | -0.06 (-0.25, 0.14) | -0.18 (-0.38, 0.02) | -0.15 (-0.35, 0.05) |
| DSS | 0.01 (-0.14, 0.16) | -0.02 (-0.17, 0.14) | 0.06 (-0.10, 0.23) | -0.12 (-0.31, 0.07) | -0.14 (-0.34, 0.05) | -0.07 (-0.27, 0.13) |
| Personal DSS | 0.04 (-0.11, 0.20) | -0.01 (-0.17, 0.14) | -0.02 (-0.18, 0.14) | -0.13 (-0.32, 0.07) | -0.17 (-0.37, 0.02) | -0.18 (-0.38, 0.02) |
| Perceived DSS | -0.02 (-0.17, 0.13) | -0.01 (-0.17, 0.14) | 0.11 (-0.05, 0.27) | -0.06 (-0.26, 0.13) | -0.05 (-0.25, 0.14) | 0.05 (-0.15, 0.26) |
| T0, pre-treatment; T1, post-treatment; T2, 3-month follow up; T3, 6-month follow up; T4, 12-month follow up.  Abbreviations: ICBT, Internet-Based Cognitive Behavioral Therapy; Vs, versus; CI, confidence intervals; PHQ-9, Patient Health Questionnaire-9; GAD-7, General Anxiety Disorder-7; K-10, Kessler Psychological Distress Scale-10; SDS, Sheehan Disability Scale; GSES, General Self-Efficacy Scale; SF-6D, Short Form 6-Dimension; DSS, Depression Stigma Scale. | | | | | | |

| **Table S5.** Sensitivity analysis 1: Observed and estimated means across time points in participants receiving ICBT treatment in the ICBT group | | | | | | | | |
| --- | --- | --- | --- | --- | --- | --- | --- | --- |
| **Outcomes** | **Pre-treatment, T0** | **Post-treatment, T1** | **3 months, T2** | **6 months, T3** | **12 months, T4** | **P-value** **^b^**  **(T0 vs.T1)** | **P-value ^b^**  **(T1 vs.T4)** |  |
| **Observed mean (SD)** |  |  |  |  |  |  |  |  |
| PHQ-9 scores | 13.91 (5.12) | 8.42 (5.70) | 9.29 (5.59) | 9.33 (6.00) | 9.47 (6.23) | / | / |  |
| GAD-7 scores | 10.63 (4.75) | 6.36 (4.92) | 7.11 (5.46) | 7.27 (5.20) | 7.08 (5.55) | / | / |  |
| K-10 scores | 30.28 (7.36) | 22.93 (9.72) | 23.95 (9.77) | 24.38 (10.10) | 24.21(10.68) | / | / |  |
| SDS scores | 15.19 (7.08) | 11.03 (7.28) | 10.14 (7.94) | 10.02 (7.94) | 9.96 (7.79) | / | / |  |
| GSES scores | 19.53 (5.34) | 21.69 (5.84) | 21.18 (6.37) | 21.68 (7.19) | 21.04 (6.86) | / | / |  |
| SF-6D scores | 0.55 (0.21) | 0.61 (0.23) | 0.62 (0.23) | 0.65 (0.21) | 0.63 (0.24) | / | / |  |
| DSS scores | 52.70 (8.98) | 52.57 (10.45) | 53.48 (9.92) | 53.87 (10.60) | 52.95 (9.93) | / | / |  |
| Personal DSS scores | 22.97 (5.21) | 22.18 (5.67) | 22.88 (5.69) | 23.44 (5.89) | 23.40 (5.90) | / | / |  |
| Perceived DSS scores | 29.73 (6.47) | 30.39 (7.32) | 30.59 (7.52) | 30.43 (8.01) | 29.55 (6.86) | / | / |  |
| **Estimated mean (SE) ^a^** |  |  |  |  |  |  |  |  |
| PHQ-9 scores | 13.91 (0.47) | 8.68 (0.50) | 9.59 (0.51) | 9.64 (0.51) | 9.72 (0.52) | <0.001*** | 0.448 |  |
| GAD-7 scores | 10.63 (0.43) | 6.35 (0.46) | 7.15 (0.46) | 7.37 (0.47) | 7.13 (0.48) | <0.001*** | 0.755 |  |
| K-10 scores | 30.28 (0.79) | 23.06 (0.83) | 24.10 (0.85) | 24.54 (0.85) | 24.19 (0.87) | <0.001*** | 0.912 |  |
| SDS scores | 15.16 (0.62) | 11.16 (0.66) | 10.40 (0.67) | 10.26 (0.68) | 10.20 (0.70) | <0.001*** | 0.596 |  |
| GSES scores | 19.53 (0.50) | 21.72 (0.54) | 21.46 (0.55) | 21.77 (0.56) | 21.12 (0.57) | <0.001*** | 0.789 |  |
| SF-6D scores | 0.55 (0.02) | 0.61 (0.02) | 0.62 (0.02) | 0.65 (0.02) | 0.63 (0.02) | 0.012* | 0.611 |  |
| DSS scores | 52.72 (0.81) | 52.34 (0.88) | 52.99 (0.91) | 53.37 (0.92) | 52.46 (0.95) | 0.994 | 1.000 |  |
| Personal DSS scores | 23.03 (0.46) | 22.06 (0.49) | 22.60 (0.51) | 23.15 (0.51) | 23.27 (0.53) | 0.220 | 0.110 |  |
| Perceived DSS scores | 29.70 (0.58) | 30.25 (0.63) | 30.33 (0.65) | 30.15 (0.66) | 29.14 (0.68) | 0.912 | 0.510 |  |
| ^a^ Estimated means are based on follow-up linear mixed models. P-values adjusted by the Bonferroni method are shown.  ^b^ **p*<.05, ** *p*<.01, *** *p*<.001.  Abbreviations: ICBT, Internet-Based Cognitive Behavioral Therapy; SD, standard deviation; SE, standard error; PHQ-9, Patient Health Questionnaire-9; GAD-7, General Anxiety Disorder-7; K-10, Kessler Psychological Distress Scale-10; SDS, Sheehan Disability Scale; GSES, General Self-Efficacy Scale; SF-6D, Short Form 6-Dimension; DSS, Depression Stigma Scale; T0, pre-treatment; T1, post-treatment; T2, 3-month follow up; T3, 6-month follow up; T4, 12-month follow up. | | | | | | | |  |

| **Table S6.** Sensitivity analysis 2: Effect size estimates for changes in outcome measures at 8 weeks and 3-, 6- and 12-month follow-up in the waitlist group | | | | | | |
| --- | --- | --- | --- | --- | --- | --- |
| **Outcomes** | **T2-T0** | **T3-T0** | **T4-T0** | **T2-T1** | **T3-T1** | **T4-T1** |
|  | **Effect size (95% *CI*)** | | | **Effect size (95% *CI*)** | | |
| PHQ-9 | 0.87 (0.61, 1.13) | 0.83 (0.57, 1.09) | 0.8 (0.53, 1.06) | -0.15 (-0.41, 0.11) | -0.15 (-0.42, 0.11) | -0.18 (-0.44, 0.09) |
| GAD-7 | 0.70(0.44, 0.95) | 0.68 (0.42, 0.94) | 0.7 (0.44, 0.96) | -0.15 (-0.4, 0.11) | -0.18 (-0.44, 0.08) | -0.14 (-0.41, 0.13) |
| K-10 | 0.75 (0.49, 1.00) | 0.69 (0.43, 0.94) | 0.69 (0.43, 0.95) | -0.10 (-0.36, 0.15) | -0.15 (-0.41, 0.12) | -0.13 (-0.39, 0.14) |
| SDS | 0.68 (0.42, 0.93) | 0.69 (0.44, 0.95) | 0.71 (0.45, 0.97) | 0.12 (-0.14, 0.38) | 0.13 (-0.13, 0.40) | 0.14 (-0.13, 0.41) |
| GSES | -0.29 (-0.53, -0.04) | -0.35 (-0.60, -0.10) | -0.25 (-0.51, 0.00) | 0.08 (-0.18, 0.35) | 0.01 (-0.26, 0.27) | 0.10 (-0.17, 0.37) |
| SF-6D | -0.32 (-0.56, -0.07) | -0.47 (-0.72, -0.21) | -0.36 (-0.62, -0.10) | -0.05 (-0.31, 0.21) | -0.18 (-0.45, 0.08) | -0.10 (-0.37, 0.18) |
| DSS | -0.08 (-0.33, 0.16) | -0.12 (-0.37, 0.13) | -0.03 (-0.28, 0.23) | -0.09 (-0.35, 0.17) | -0.12 (-0.39, 0.14) | -0.04 (-0.31, 0.23) |
| Personal DSS | 0.02 (-0.23, 0.26) | -0.08 (-0.33, 0.17) | -0.08 (-0.33, 0.18) | -0.12 (-0.38, 0.14) | -0.22 (-0.48, 0.04) | -0.21 (-0.48, 0.06) |
| Perceived DSS | -0.12 (-0.37, 0.12) | -0.10 (-0.35, 0.15) | 0.03 (-0.23, 0.28) | -0.03 (-0.29, 0.23) | 0.01 (-0.27, 0.26) | 0.12 (-0.15, 0.39) |
| T0, pre-treatment; T1, post-treatment; T2, 3-month follow up; T3, 6-month follow up; T4, 12-month follow up.  Abbreviations: ICBT, Internet-Based Cognitive Behavioral Therapy; Vs, versus; CI, confidence intervals; PHQ-9, Patient Health Questionnaire-9; GAD-7, General Anxiety Disorder-7; K-10, Kessler Psychological Distress Scale-10; SDS, Sheehan Disability Scale; GSES, General Self-Efficacy Scale; SF-6D, Short Form 6-Dimension; DSS, Depression Stigma Scale. | | | | | | |

| **Table S7.** Sensitivity analysis 3: Observed and estimated means across time points in participants receiving ICBT treatment in the waitlist control group | | | | | | | | |
| --- | --- | --- | --- | --- | --- | --- | --- | --- |
| **Outcomes** | **Pre-treatment, T0** | **Post-treatment, T1** | **3 months, T2** | **6 months, T3** | **12 months, T4** | **P-value** **^b^**  **(T0 vs.T1)** | **P-value ^b^**  **(T1 vs.T4)** |  |
| **Observed mean (SD)** |  |  |  |  |  |  |  |  |
| PHQ-9 scores | 13.06 (4.70) | 8.90 (5.37) | 9.48 (5.80) | 10.16 (5.89) | 9.52 (5.44) | / | / |  |
| GAD-7 scores | 9.17 (4.61) | 6.61 (4.87) | 7.62 (5.47) | 7.48 (5.66) | 7.15 (5.49) | / | / |  |
| K-10 scores | 27.96 (8.34) | 23.66 (8.65) | 23.98 (9.83) | 25.02 (9.99) | 25.04 (8.93) | / | / |  |
| SDS scores | 13.68 (6.98) | 10.12 (6.36) | 9.96 (7.63) | 9.53 (7.11) | 9.04 (7.17) | / | / |  |
| GSES scores | 20.18 (5.82) | 20.83 (5.61) | 20.83 (6.09) | 20.70 (5.86) | 20.95 (7.01) | / | / |  |
| SF-6D scores | 0.60 (0.18) | 0.62 (0.22) | 0.63 (0.23) | 0.65 (0.22) | 0.67 (0.20) | / | / |  |
| DSS scores | 54.66 (8.74) | 52.71 (9.82) | 54.31 (8.89) | 54.40 (9.00) | 53.83 (10.37) | / | / |  |
| Personal DSS scores | 23.22 (5.41) | 22.26 (5.60) | 23.12 (5.82) | 23.13 (6.47) | 23.21 (7.05) | / | / |  |
| Perceived DSS scores | 31.44 (6.49) | 30.46 (7.34) | 31.19 (6.38) | 31.27 (6.72) | 30.61 (7.13) | / | / |  |
| **Estimated mean (SE) ^a^** |  |  |  |  |  |  |  |  |
| PHQ-9 scores | 13.19 (0.54) | 9.20 (0.56) | 9.52 (0.56) | 10.15 (0.56) | 9.67 (0.58) | <0.001*** | 0.996 |  |
| GAD-7 scores | 9.28 (0.48) | 6.82 (0.50) | 7.61 (0.50) | 7.48 (0.50) | 7.25 (0.52) | <0.001*** | 0.998 |  |
| K-10 scores | 28.16 (0.89) | 23.94 (0.92) | 23.82 (0.92) | 24.80 (0.92) | 24.98 (0.96) | <0.001*** | 0.972 |  |
| SDS scores | 13.78 (0.68) | 10.48 (0.72) | 10.12 (0.71) | 9.63 (0.71) | 9.65 (0.74) | <0.001*** | 0.679 |  |
| GSES scores | 20.13 (0.58) | 20.78 (0.62) | 20.88 (0.62) | 20.74 (0.62) | 21.36 (0.65) | 0.767 | 0.868 |  |
| SF-6D scores | 0.60 (0.02) | 0.61 (0.02) | 0.63 (0.02) | 0.65 (0.02) | 0.66 (0.02) | 0.986 | 0.031* |  |
| DSS scores | 54.72 (0.88) | 52.71 (0.93) | 54.17 (0.93) | 54.24 (0.93) | 53.71 (0.98) | 0.110 | 0.796 |  |
| Personal DSS scores | 23.31 (0.56) | 22.45 (0.59) | 23.19 (0.59) | 23.22 (0.60) | 23.32 (0.62) | 0.412 | 0.476 |  |
| Perceived DSS scores | 31.41 (0.64) | 30.25 (0.67) | 30.96 (0.67) | 31.00 (0.68) | 30.38 (0.70) | 0.216 | 1.000 |  |
| ^a^ Estimated means are based on follow-up linear mixed models. P-values adjusted by the Bonferroni method are shown.  ^b^ **p*<.05, ** *p*<.01, *** *p*<.001.  Abbreviations: ICBT, Internet-Based Cognitive Behavioral Therapy; SD, standard deviation; SE, standard error; PHQ-9, Patient Health Questionnaire-9; GAD-7, General Anxiety Disorder-7; K-10, Kessler Psychological Distress Scale-10; SDS, Sheehan Disability Scale; GSES, General Self-Efficacy Scale; SF-6D, Short Form 6-Dimension; DSS, Depression Stigma Scale; T0, pre-treatment; T1, post-treatment; T2, 3-month follow up; T3, 6-month follow up; T4, 12-month follow up. | | | | | | | |  |

| **Table S8.** Sensitivity analysis 2: Effect size estimates for changes in outcome measures at 8 weeks and 3-, 6- and 12-month follow-up in the waitlist control group | | | | | | |
| --- | --- | --- | --- | --- | --- | --- |
| **Outcomes** | **T2-T0** | **T3-T0** | **T4-T0** | **T2-T1** | **T3-T1** | **T4-T1** |
|  | **Effect size (95% *CI*)** | | | **Effect size (95% *CI*)** | | |
| PHQ-9 | 0.49 (0.26, 0.72) | 0.36 (0.13, 0.59) | 0.49 (0.25, 0.74) | -0.10 (-0.40, 0.19) | -0.22 (-0.52, 0.07) | -0.11 (-0.42, 0.19) |
| GAD-7 | 0.25 (0.02, 0.48) | 0.28 (0.05, 0.51) | 0.35 (0.10, 0.59) | -0.20 (-0.49, 0.10) | -0.17 (-0.46, 0.13) | -0.10 (-0.41, 0.21) |
| K-10 | 0.47 (0.25, 0.70) | 0.35 (0.12, 0.58) | 0.36 (0.11, 0.60) | -0.03 (-0.33, 0.26) | -0.15 (-0.44, 0.15) | -0.16 (-0.47, 0.15) |
| SDS | 0.70 (0.47, 0.93) | 0.77 (0.54, 1.00) | 0.84 (0.59, 1.09) | 0.02 (-0.27, 0.32) | 0.09 (-0.21, 0.39) | 0.16 (-0.15, 0.47) |
| GSES | -0.12 (-0.35, 0.11) | -0.25 (-0.48, -0.02) | -0.34 (-0.58, -0.09) | -0.05 (-0.35, 0.25) | -0.16 (-0.45, 0.14) | -0.23 (-0.55, 0.08) |
| SF-6D | -0.12 (-0.35, 0.11) | -0.10 (-0.33, 0.13) | -0.14 (-0.39, 0.10) | 0.01 (-0.30, 0.30) | 0.02 (-0.27, 0.32) | -0.02 (-0.33, 0.29) |
| DSS | 0.05 (-0.17, 0.28) | 0.04 (-0.19, 0.27) | 0.10 (-0.14, 0.35) | -0.17 (-0.47, 0.12) | -0.18 (-0.48, 0.12) | -0.11 (-0.42, 0.20) |
| Personal DSS | 0.01 (-0.22, 0.23) | 0.01 (-0.23, 0.23) | -0.01 (-0.26, 0.23) | -0.15 (-0.45, 0.14) | -0.15 (-0.44, 0.15) | -0.15 (-0.46, 0.16) |
| Perceived DSS | 0.07 (-0.16, 0.29) | 0.06 (-0.17, 0.28) | 0.15 (-0.09, 0.40) | -0.11 (-0.40, 0.19) | -0.12 (-0.41, 0.18) | -0.02 (-0.33, 0.29) |
| T0, pre-treatment; T1, post-treatment; T2, 3-month follow up; T3, 6-month follow up; T4, 12-month follow up.  Abbreviations: ICBT, Internet-Based Cognitive Behavioral Therapy; Vs, versus; CI, confidence intervals; PHQ-9, Patient Health Questionnaire-9; GAD-7, General Anxiety Disorder-7; K-10, Kessler Psychological Distress Scale-10; SDS, Sheehan Disability Scale; GSES, General Self-Efficacy Scale; SF-6D, Short Form 6-Dimension; DSS, Depression Stigma Scale. | | | | | | |

| **Table S9.** Remission and response rate in the ICBT and waitlist control group | | | | |
| --- | --- | --- | --- | --- |
| **Time points** | **Overall (N=269)** | **ICBT (N=121)** | **WLC (N=148)** | ***P*-value ^c^** |
| **Remission ^a^** |  |  |  |  |
| T1-T0 | 138 (51.3) | 80 (66.1) | 58 (39.2) | <0.001*** |
| T2-T0 | 65 (58.6) | 65 (58.6) | / |  |
| T3-T0 | 67 (62.6) | 67 (62.6) | / |  |
| T4-T0 | 57 (58.2) | 57 (58.2) | / |  |
| **Response ^b^** |  |  |  |  |
| T1-T0 | 77 (28.6) | 50 (41.3) | 27 (18.2) | <0.001*** |
| T2-T0 | 33 (29.7) | 33 (29.7) | / |  |
| T3-T0 | 41 (38.3) | 41 (38.3) | / |  |
| T4-T0 | 32 (32.7) | 32 (32.7) | / |  |
| T0, pre-treatment; T1, post-treatment; T2, 3-month follow up; T3, 6-month follow up; T4, 12-month follow up.  ^a^ Remission is defined as a PHQ-9 score less than 10.  ^b^ Response is defined as a reduction of PHQ-9 score more than 50% in comparison with PHQ-9 at baseline.  ^c^ *** p<.001. | | | | |

**Figure S1.** Sensitivity analysis 1: Estimated marginal means and 95% confidence intervals (CIs) across all-time points for participants receiving ICBT intervention in the ICBT group


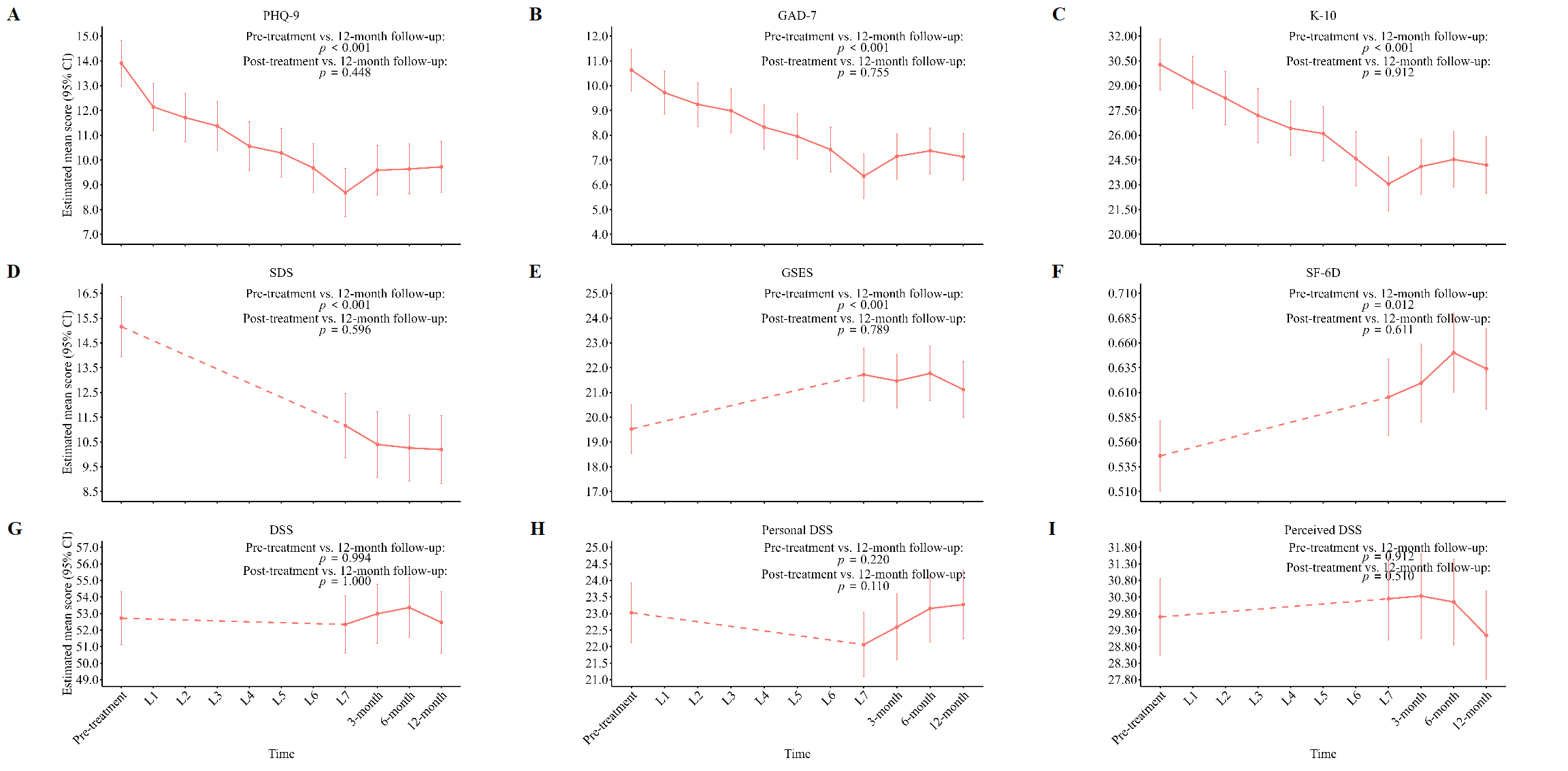


**Figure S2.** Sensitivity analysis 1: Estimated marginal means and 95% confidence intervals (CIs) across all-time points for participants receiving ICBT intervention in the waitlist control group


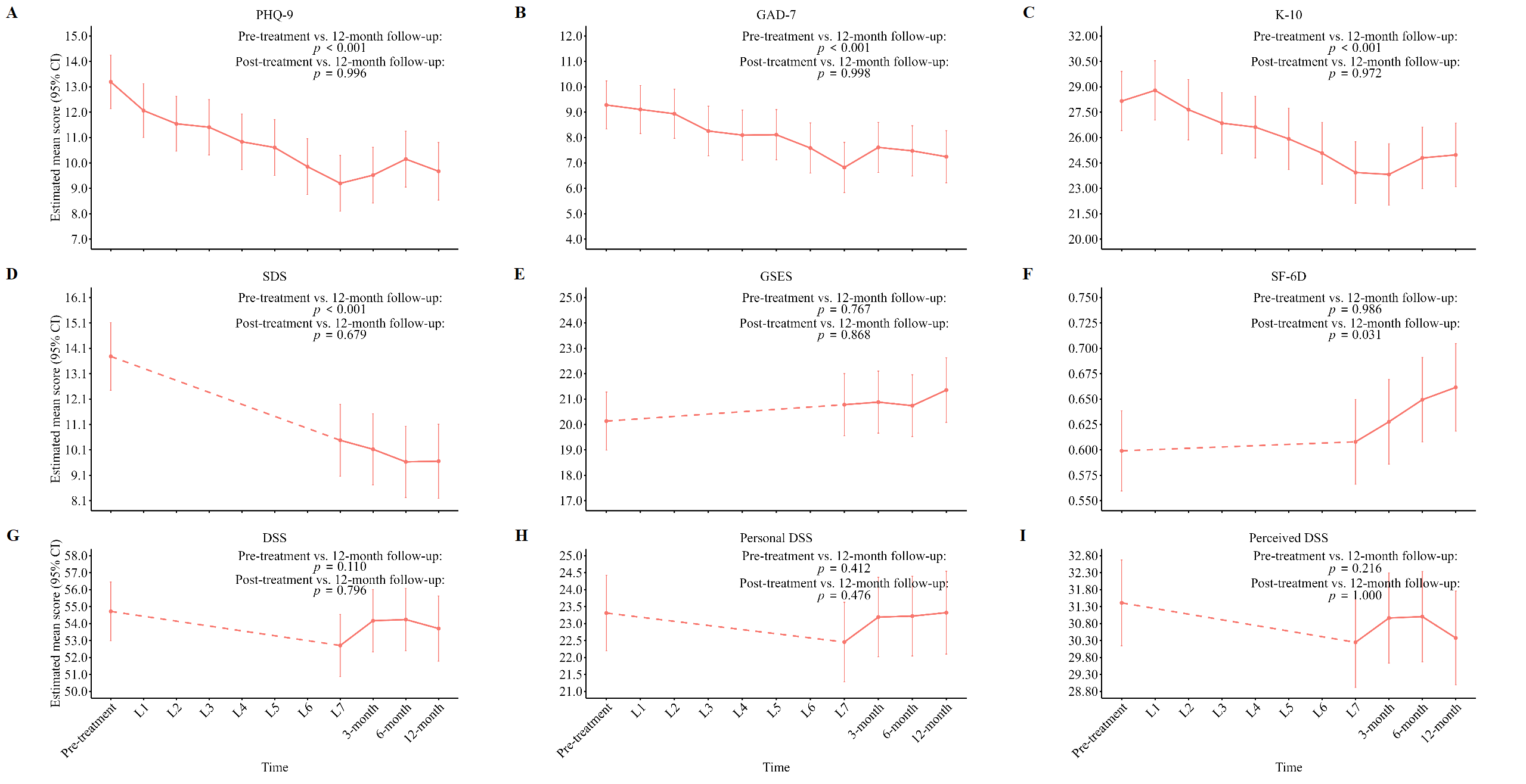

Supplement: Multimedia Appendix 5 [file mhealth-v14-e68394-s005.docx]
