# Supplementary material for: Long-Term Effectiveness of Unguided Internet-Based Cognitive Behavioral Therapy on Major Depressive Disorder in Chinese Adults: Randomized Controlled Trial With a 12-Month Follow-Up
Source: JMIR Mhealth Uhealth. 2026 Jun 24;14:e68394. doi: 10.2196/68394 (PMC13293601; doi:10.2196/68394)
Supplement: Checklist 1 [file mhealth-v14-e68394-s006.pdf]

CONSORT-EHEALTH (V 1.6.1) - Submission/Publication Form

The CONSORT-EHEALTH checklist is intended for authors of randomized trials evaluating web-based and Internet-based applications/interventions, including mobile interventions, electronic games (incl multiplayer games), social media, certain telehealth applications, and other interactive and/or networked electronic applications. Some of the items (e.g. all subitems under item 5 - description of the intervention) may also be applicable for other study designs.

The goal of the CONSORT EHEALTH checklist and guideline is to be
a) a guide for reporting for authors of RCTs,
b) to form a basis for appraisal of an ehealth trial (in terms of validity)

CONSORT-EHEALTH items/subitems are MANDATORY reporting items for studies published in the Journal of Medical Internet Research and other journals / scientific societies endorsing the checklist.

Items numbered 1., 2., 3., 4a., 4b etc are original CONSORT or CONSORT-NPT (non-pharmacologic treatment) items.
Items with Roman numerals (i., ii, iii, iv etc.) are CONSORT-EHEALTH extensions/clarifications.

As the CONSORT-EHEALTH checklist is still considered in a formative stage, we would ask that you also RATE ON A SCALE OF 1-5 how important/useful you feel each item is FOR THE PURPOSE OF THE CHECKLIST and reporting guideline (optional).

Mandatory reporting items are marked with a red *.
In the textboxes, either copy & paste the relevant sections from your manuscript into this form - please include any quotes from your manuscript in QUOTATION MARKS,
or answer directly by providing additional information not in the manuscript, or elaborating on why the item was not relevant for this study.

YOUR ANSWERS WILL BE PUBLISHED AS A SUPPLEMENTARY FILE TO YOUR PUBLICATION IN JMIR AND ARE CONSIDERED PART OF YOUR PUBLICATION (IF ACCEPTED).
Please fill in these questions diligently. Information will not be copyedited, so please use proper spelling and grammar, use correct capitalization, and avoid abbreviations.

DO NOT FORGET TO SAVE AS PDF _AND_ CLICK THE SUBMIT BUTTON SO YOUR ANSWERS ARE IN OUR DATABASE !!!

Citation Suggestion (if you append the pdf as Appendix we suggest to cite this paper in the caption):
Eysenbach G, CONSORT-EHEALTH Group
CONSORT-EHEALTH: Improving and Standardizing Evaluation Reports of Web-based and Mobile Health Interventions
J Med Internet Res 2011;13(4):e126

URL: http://www.jmir.org/2011/4/e126/
doi: 10.2196/jmir.1923 IF: 5.8 Q1 PMID: 22209829

登录 Google 即可保存进度。了解详情

* 表示必填

Your name *

First Last

Wenjing Zhou

Primary Affiliation (short), City, Country *

University of Toronto, Toronto, Canada

University of Sun Yat-sen University

Your e-mail address *

abc@gmail.com

zhouwj55@mail2.sysu.edu.cn

Title of your manuscript *

Provide the (draft) title of your manuscript.

Long-Term Effectiveness of Unguided Internet-Based Cognitive Behavioral Therapy on Major Depressive Disorder in Chinese Adults: A Randomized Controlled Trial with A 12-month Follow-Up

Name of your App/Software/Intervention *

If there is a short and a long/alternate name, write the short name first and add the long name in brackets.

Morning Mood

Evaluated Version (if any)

e.g. "V1", "Release 2017-03-01", "Version 2.0.27913"

您的回答

Language(s) *

What language is the intervention/app in? If multiple languages are available, separate by comma (e.g. "English, French")

Chinese

URL of your Intervention Website or App

e.g. a direct link to the mobile app on app in appstore (itunes, Google Play), or URL of the website. If the intervention is a DVD or hardware, you can also link to an Amazon page.

您的回答

URL of an image/screenshot (optional)

https://www.ncbi.nlm.nih.gov/pubmed/37738092

Accessibility *

Can an enduser access the intervention presently?

- ( ) access is free and open
- (•) access only for special usergroups, not open
- ( ) access is open to everyone, but requires payment/subscription/in-app purchases
- ( ) app/intervention no longer accessible
- ( ) 其他:

Primary Medical Indication/Disease/Condition *

e.g. "Stress", "Diabetes", or define the target group in brackets after the condition, e.g. "Autism (Parents of children with)", "Alzheimers (Informal Caregivers of)"

Depression

Primary Outcomes measured in trial *

comma-separated list of primary outcomes reported in the trial

depressive symptoms measured by PHQ-9

Secondary/other outcomes

Are there any other outcomes the intervention is expected to affect?

Other disease-related symptoms (anxiety, psychological distress), individual and social functioning, quality of life

Recommended "Dose" *

What do the instructions for users say on how often the app should be used?

- ○ Approximately Daily
- ◉ Approximately Weekly
- ○ Approximately Monthly
- ○ Approximately Yearly
- ○ "as needed"
- ○ 其他:

Approx. Percentage of Users (starters) still using the app as recommended after 3 months *

- ◉ unknown / not evaluated
- ○ 0-10%
- ○ 11-20%
- ○ 21-30%
- ○ 31-40%
- ○ 41-50%
- ○ 51-60%
- ○ 61-70%
- ○ 71%-80%
- ○ 81-90%
- ○ 91-100%
- ○ 其他:

Overall, was the app/intervention effective? *

- (selected) yes: all primary outcomes were significantly better in intervention group vs control
- partly: SOME primary outcomes were significantly better in intervention group vs control
- no statistically significant difference between control and intervention
- potentially harmful: control was significantly better than intervention in one or more outcomes
- inconclusive: more research is needed
- 其他:

Article Preparation Status/Stage *

At which stage in your article preparation are you currently (at the time you fill in this form)

- not submitted yet - in early draft status
- (selected) not submitted yet - in late draft status, just before submission
- submitted to a journal but not reviewed yet
- submitted to a journal and after receiving initial reviewer comments
- submitted to a journal and accepted, but not published yet
- published
- 其他:

Journal *

If you already know where you will submit this paper (or if it is already submitted), please provide the journal name (if it is not JMIR, provide the journal name under "other")

- ○ not submitted yet / unclear where I will submit this
- ○ Journal of Medical Internet Research (JMIR)
- ◉ JMIR mHealth and UHealth
- ○ JMIR Serious Games
- ○ JMIR Mental Health
- ○ JMIR Public Health
- ○ JMIR Formative Research
- ○ Other JMIR sister journal
- ○ 其他:

Is this a full powered effectiveness trial or a pilot/feasibility trial? *

- ○ Pilot/feasibility
- ◉ Fully powered

Manuscript tracking number *

If this is a JMIR submission, please provide the manuscript tracking number under "other" (The ms tracking number can be found in the submission acknowledgement email, or when you login as author in JMIR. If the paper is already published in JMIR, then the ms tracking number is the four-digit number at the end of the DOI, to be found at the bottom of each published article in JMIR)

- ◉ no ms number (yet) / not (yet) submitted to / published in JMIR
- ○ 其他:

TITLE AND ABSTRACT

1a) TITLE: Identification as a randomized trial in the title

1a) Does your paper address CONSORT item 1a? *

I.e does the title contain the phrase "Randomized Controlled Trial"? (if not, explain the reason under "other")

- (●) yes
- ( ) 其他:

1a-i) Identify the mode of delivery in the title

Identify the mode of delivery. Preferably use “web-based” and/or “mobile” and/or “electronic game” in the title. Avoid ambiguous terms like “online”, “virtual”, “interactive”. Use “Internet-based” only if Intervention includes non-web-based Internet components (e.g. email), use “computer-based” or “electronic” only if offline products are used. Use “virtual” only in the context of “virtual reality” (3-D worlds). Use “online” only in the context of “online support groups”. Complement or substitute product names with broader terms for the class of products (such as “mobile” or “smart phone” instead of “iphone”), especially if the application runs on different platforms.

	1	2	3	4	5	
subitem not at all important	( )	( )	( )	( )	( )	essential

Does your paper address subitem 1a-i? *

Copy and paste relevant sections from manuscript title (include quotes in quotation marks "like this" to indicate direct quotes from your manuscript), or elaborate on this item by providing additional information not in the ms, or briefly explain why the item is not applicable/relevant for your study

"Unguided Internet-Based Cognitive Behavioral Therapy"

1a-ii) Non-web-based components or important co-interventions in title

Mention non-web-based components or important co-interventions in title, if any (e.g., “with telephone support”).

	1	2	3	4	5	
subitem not at all important	○	○	○	○	○	essential

Does your paper address subitem 1a-ii?

Copy and paste relevant sections from manuscript title (include quotes in quotation marks "like this" to indicate direct quotes from your manuscript), or elaborate on this item by providing additional information not in the ms, or briefly explain why the item is not applicable/relevant for your study

No, the ICBT intervention in our analysis is an unguided Internet-Based Cognitive Behavioral Therapy with general support from non-specialists and no additional treatment support was provided.

1a-iii) Primary condition or target group in the title

Mention primary condition or target group in the title, if any (e.g., “for children with Type I Diabetes”) Example: A Web-based and Mobile Intervention with Telephone Support for Children with Type I Diabetes: Randomized Controlled Trial

	1	2	3	4	5	
subitem not at all important	○	○	○	○	○	essential

Does your paper address subitem 1a-iii? *

Copy and paste relevant sections from manuscript title (include quotes in quotation marks "like this" to indicate direct quotes from your manuscript), or elaborate on this item by providing additional information not in the ms, or briefly explain why the item is not applicable/relevant for your study

"Major Depressive Disorder in Chinese Adults"

1b) ABSTRACT: Structured summary of trial design, methods, results, and conclusions

NPT extension: Description of experimental treatment, comparator, care providers, centers, and blinding status.

1b-i) Key features/functionalities/components of the intervention and comparator in the METHODS section of the ABSTRACT

Mention key features/functionalities/components of the intervention and comparator in the abstract. If possible, also mention theories and principles used for designing the site. Keep in mind the needs of systematic reviewers and indexers by including important synonyms. (Note: Only report in the abstract what the main paper is reporting. If this information is missing from the main body of text, consider adding it)

	1	2	3	4	5	
subitem not at all important	○	○	○	○	○	essential

Does your paper address subitem 1b-i? *

Copy and paste relevant sections from the manuscript abstract (include quotes in quotation marks "like this" to indicate direct quotes from your manuscript), or elaborate on this item by providing additional information not in the ms, or briefly explain why the item is not applicable/relevant for your study

"This randomized controlled trial (ChiCTR2100046425) was conducted in participants with MDD between August 2021 and June 2023 in Shenzhen, China, with 159 participants in the ICBT group and 158 in the waiting-list control (WLC) group. The ICBT group was intervened with usual care and a 7-module ICBT course for 8 weeks and was followed up for 12 months after treatment completion. The WLC group was provided with the usual care during the intervention period and then started the ICBT course after 8 weeks."

1b-ii) Level of human involvement in the METHODS section of the ABSTRACT

Clarify the level of human involvement in the abstract, e.g., use phrases like “fully automated” vs. “therapist/nurse/care provider/physician-assisted” (mention number and expertise of providers involved, if any). (Note: Only report in the abstract what the main paper is reporting. If this information is missing from the main body of text, consider adding it)

	1	2	3	4	5	
subitem not at all important	○	○	○	○	○	essential

Does your paper address subitem 1b-ii?

Copy and paste relevant sections from the manuscript abstract (include quotes in quotation marks "like this" to indicate direct quotes from your manuscript), or elaborate on this item by providing additional information not in the ms, or briefly explain why the item is not applicable/relevant for your study

No, the ICBT intervention in our analysis is an unguided Internet-Based Cognitive Behavioral Therapy with general support from non-specialists and no additional treatment support was provided.

1b-iii) Open vs. closed, web-based (self-assessment) vs. face-to-face assessments in the METHODS section of the ABSTRACT

Mention how participants were recruited (online vs. offline), e.g., from an open access website or from a clinic or a closed online user group (closed usergroup trial), and clarify if this was a purely web-based trial, or there were face-to-face components (as part of the intervention or for assessment). Clearly say if outcomes were self-assessed through questionnaires (as common in web-based trials). Note: In traditional offline trials, an open trial (open-label trial) is a type of clinical trial in which both the researchers and participants know which treatment is being administered. To avoid confusion, use “blinded” or “unblinded” to indicated the level of blinding instead of “open”, as “open” in web-based trials usually refers to “open access” (i.e. participants can self-enrol). (Note: Only report in the abstract what the main paper is reporting. If this information is missing from the main body of text, consider adding it)

	1	2	3	4	5	
subitem not at all important	○	○	○	○	○	essential

Does your paper address subitem 1b-iii?

Copy and paste relevant sections from the manuscript abstract (include quotes in quotation marks "like this" to indicate direct quotes from your manuscript), or elaborate on this item by providing additional information not in the ms, or briefly explain why the item is not applicable/relevant for your study

"unguided Internet-Based Cognitive Behavioral Therapy"

1b-iv) RESULTS section in abstract must contain use data

Report number of participants enrolled/assessed in each group, the use/uptake of the intervention (e.g., attrition/adherence metrics, use over time, number of logins etc.), in addition to primary/secondary outcomes. (Note: Only report in the abstract what the main paper is reporting. If this information is missing from the main body of text, consider adding it)

	1	2	3	4	5	
subitem not at all important	○	○	○	○	○	essential

Does your paper address subitem 1b-iv?

Copy and paste relevant sections from the manuscript abstract (include quotes in quotation marks "like this" to indicate direct quotes from your manuscript), or elaborate on this item by providing additional information not in the ms, or briefly explain why the item is not applicable/relevant for your study

"According to the intention-to-treat principle, 152 participants in the ICBT group and 148 participants in the WLC group were involved in the analysis. The mean age was 28.49 (7.00) years and the female-to-male ratio was 3:1. Dropout rates were 20.4% for the ICBT group and 6.3% for the WLC group."

1b-v) CONCLUSIONS/DISCUSSION in abstract for negative trials

Conclusions/Discussions in abstract for negative trials: Discuss the primary outcome - if the trial is negative (primary outcome not changed), and the intervention was not used, discuss whether negative results are attributable to lack of uptake and discuss reasons. (Note: Only report in the abstract what the main paper is reporting. If this information is missing from the main body of text, consider adding it)

	1	2	3	4	5	
subitem not at all important	○	○	○	○	○	essential

Does your paper address subitem 1b-v?

Copy and paste relevant sections from the manuscript abstract (include quotes in quotation marks "like this" to indicate direct quotes from your manuscript), or elaborate on this item by providing additional information not in the ms, or briefly explain why the item is not applicable/relevant for your study

No negative results

Your answer must have a minimum of 25 characters.

INTRODUCTION

2a) In INTRODUCTION: Scientific background and explanation of rationale

2a-i) Problem and the type of system/solution

Describe the problem and the type of system/solution that is object of the study: intended as stand-alone intervention vs. incorporated in broader health care program? Intended for a particular patient population? Goals of the intervention, e.g., being more cost-effective to other interventions, replace or complement other solutions? (Note: Details about the intervention are provided in “Methods” under 5)

	1	2	3	4	5	
subitem not at all important	○	○	○	○	○	essential

Does your paper address subitem 2a-i? *

Copy and paste relevant sections from the manuscript (include quotes in quotation marks "like this" to indicate direct quotes from your manuscript), or elaborate on this item by providing additional information not in the ms, or briefly explain why the item is not applicable/relevant for your study

"Internet-based cognitive behavioral therapy (ICBT), a scalable, confidential, low-cost, and flexible modality of cognitive behavioral therapy (CBT) delivered through the Internet with guided and unguided forms, has been introduced to the treatment of MDD to overcome the treatment barriers mentioned above.[9]The unguided form which is defined as self-help ICBT with different levels of technical support, leads itself to greater cost-effectiveness and easier implementation in comparison to in-person CBT or guided CBT.[10]Consequently, the supply-demand imbalance for in-person mental health resources in China provided the impetus for our team to develop an unguided ICBT course for MDD called Morning Mood on WeChat Mini Program."

2a-ii) Scientific background, rationale: What is known about the (type of) system

Scientific background, rationale: What is known about the (type of) system that is the object of the study (be sure to discuss the use of similar systems for other conditions/diagnoses, if appropiate), motivation for the study, i.e. what are the reasons for and what is the context for this specific study, from which stakeholder viewpoint is the study performed, potential impact of findings [2]. Briefly justify the choice of the comparator.

	1	2	3	4	5	
subitem not at all important	○	○	○	○	○	essential

Does your paper address subitem 2a-ii? *

Copy and paste relevant sections from the manuscript (include quotes in quotation marks "like this" to indicate direct quotes from your manuscript), or elaborate on this item by providing additional information not in the ms, or briefly explain why the item is not applicable/relevant for your study

"Although previous randomized controlled trials (RCTs)[10–27] have investigated the short- (i.e., post-treatment) and long-term effectiveness of unguided ICBT on MDD, a large variance is observed in reported short-term effect sizes, ranging from no effect[19] to large effect sizes.[18,24,25]Moreover, the conclusions on long-term effectiveness are inconsistent. For example, Newby et al.,[22]found that the depressive outcomes at the post-treatment stage were favored over the 3-month follow-up stage, with a within-group effect size of -0.21, while Gilbody et al.,[19]reported the within-group size between the post-treatment and 12-month follow-up stage was 0.35. Extant studies evaluating ICBT are largely represented by Western studies, thereby limiting the ability to generalize the results to patients from different races, ethnicities, and cultural backgrounds.[28]Furthermore, the aforementioned studies have primarily focused on depressive symptomatology outcomes, limiting possible insights into other relevant depression domains (e.g., anxiety symptoms, self-efficacy, quality of life, and functional outcomes).[29]In China, only one RCT has verified the short-term effectiveness of clinician-supported unguided ICBT (the Chinese-translated version of MoodGYM) in patients with MDD. [27] Methodological and generalizability limitations of extant studies (e.g., sample size, short-term observation, cultural adaptability), there is the need to evaluate the short- and long-term effectiveness of unguided ICBT for MDD in China, especially the unguided ICBT delivered by non-specialists."

2b) In INTRODUCTION: Specific objectives or hypotheses

Does your paper address CONSORT subitem 2b? *

Copy and paste relevant sections from the manuscript (include quotes in quotation marks "like this" to indicate direct quotes from your manuscript), or elaborate on this item by providing additional information not in the ms, or briefly explain why the item is not applicable/relevant for your study

"Herein, we aimed to (1) evaluate the short- and long-term effectiveness of unguided ICBT in treating adults with MDD; (2) further evaluate the short- and long-term effects on specific depressive symptoms as well as individual and social functioning as well as quality of life; (3) assess the acceptability and satisfaction among participants in the ICBT group."

METHODS

3a) Description of trial design (such as parallel, factorial) including allocation ratio

Does your paper address CONSORT subitem 3a? *

Copy and paste relevant sections from the manuscript (include quotes in quotation marks "like this" to indicate direct quotes from your manuscript), or elaborate on this item by providing additional information not in the ms, or briefly explain why the item is not applicable/relevant for your study

"This study was an 8-week non-blinded pragmatic RCT with 12 months follow-up investigating whether an unguided ICBT course as an adjunct to usual care could reduce depression symptoms in adult patients with MDD and its long-term effectiveness."

3b) Important changes to methods after trial commencement (such as eligibility criteria), with reasons

Does your paper address CONSORT subitem 3b? *

Copy and paste relevant sections from the manuscript (include quotes in quotation marks "like this" to indicate direct quotes from your manuscript), or elaborate on this item by providing additional information not in the ms, or briefly explain why the item is not applicable/relevant for your study

No change was made after trial commencement

3b-i) Bug fixes, Downtimes, Content Changes

Bug fixes, Downtimes, Content Changes: ehealth systems are often dynamic systems. A description of changes to methods therefore also includes important changes made on the intervention or comparator during the trial (e.g., major bug fixes or changes in the functionality or content) (5-iii) and other “unexpected events” that may have influenced study design such as staff changes, system failures/downtimes, etc. [2].

	1	2	3	4	5	
subitem not at all important	○	○	○	○	○	essential

Does your paper address subitem 3b-i?

Copy and paste relevant sections from the manuscript (include quotes in quotation marks "like this" to indicate direct quotes from your manuscript), or elaborate on this item by providing additional information not in the ms, or briefly explain why the item is not applicable/relevant for your study

No bug fixes, downtimes and content changes

4a) Eligibility criteria for participants

Does your paper address CONSORT subitem 4a? *

Copy and paste relevant sections from the manuscript (include quotes in quotation marks "like this" to indicate direct quotes from your manuscript), or elaborate on this item by providing additional information not in the ms, or briefly explain why the item is not applicable/relevant for your study

"The key inclusion criteria were: (1) Chinese residents; (2) aged between 18 and 60 years; (3) met criteria for MDD in Diagnostic and Statistical Manual of Mental Disorders-Fourth Edition (DSM-IV); (4) had access to Internet; (5) a score of Patient Health Questionnaire-9 (PHQ-9) more than 5; (6)no severe suicidal risk according to section C of the M.I.N.I; (7) in a stable condition (if taking antidepressants, dosage had to be stable for at least 30 days before enrollment). The key exclusion criteria were: (1) met DSM-IV criteria for bipolar disorder or other psychotic disorders; (2) had an ongoing alcohol or substance abuse disorder; (3) was pregnant or lactating; (4) underwent physiotherapy or psychotherapy treatments at the time of enrollment. "

4a-i) Computer / Internet literacy

Computer / Internet literacy is often an implicit “de facto” eligibility criterion - this should be explicitly clarified.

	1	2	3	4	5	
subitem not at all important	○	○	○	○	○	essential

Does your paper address subitem 4a-i?

Copy and paste relevant sections from the manuscript (include quotes in quotation marks "like this" to indicate direct quotes from your manuscript), or elaborate on this item by providing additional information not in the ms, or briefly explain why the item is not applicable/relevant for your study

" (4) had access to Internet;"

4a-ii) Open vs. closed, web-based vs. face-to-face assessments:

Open vs. closed, web-based vs. face-to-face assessments: Mention how participants were recruited (online vs. offline), e.g., from an open access website or from a clinic, and clarify if this was a purely web-based trial, or there were face-to-face components (as part of the intervention or for assessment), i.e., to what degree got the study team to know the participant. In online-only trials, clarify if participants were quasi-anonymous and whether having multiple identities was possible or whether technical or logistical measures (e.g., cookies, email confirmation, phone calls) were used to detect/prevent these.

	1	2	3	4	5	
subitem not at all important	○	○	○	○	○	essential

Does your paper address subitem 4a-ii? *

Copy and paste relevant sections from the manuscript (include quotes in quotation marks "like this" to indicate direct quotes from your manuscript), or elaborate on this item by providing additional information not in the ms, or briefly explain why the item is not applicable/relevant for your study

"The ICBT intervention in this trial was tailored to the Chinese population aiming at teaching practical CBT skills for depression. The self-developed ICBT course was designed as a Mini Program called Morning Mood on WeChat, one of the most popular messaging apps in China. In total, there were 7 lessons, covering psychoeducation, behavioral activation, cognitive restructuring, affect regulation, and relapse prevention. The detailed content of each lesson was demonstrated in another publication.[32]Participants in the ICBT group were required to complete one lesson per week; one week after completing the prior lesson, the subsequent lesson would be open automatically. This setting was used to prevent participants from completing multiple lessons in a short period, which might potentially impact the effectiveness. General support involving technical support and daily reminders provided by non-specialists (e.g., nurses, lay health workers, and social workers) via telephone or WeChat were applied to prompt engagement."

4a-iii) Information giving during recruitment

Information given during recruitment. Specify how participants were briefed for recruitment and in the informed consent procedures (e.g., publish the informed consent documentation as appendix, see also item X26), as this information may have an effect on user self-selection, user expectation and may also bias results.

	1	2	3	4	5	
subitem not at all important	○	○	○	○	○	essential

Does your paper address subitem 4a-iii?

Copy and paste relevant sections from the manuscript (include quotes in quotation marks "like this" to indicate direct quotes from your manuscript), or elaborate on this item by providing additional information not in the ms, or briefly explain why the item is not applicable/relevant for your study

"Eligible participants were then instructed to complete baseline assessments. Sociodemographic information (e.g., age, gender, and ethnicity), lifestyle features (e.g., exercise, drinking, and smoking), clinical characteristics (e.g., antidepressants use, first of onset, and age of MDD onset), and baseline symptoms (e.g., somatic pain, resilience, and insomnia severity) were collected. The randomization was applied by using an online random number generator, with a ratio of 1:1 in the ICBT and WLC groups. Due to the nature of the interventions, the blind method was hard to carry out."

4b) Settings and locations where the data were collected

Does your paper address CONSORT subitem 4b? *

Copy and paste relevant sections from the manuscript (include quotes in quotation marks "like this" to indicate direct quotes from your manuscript), or elaborate on this item by providing additional information not in the ms, or briefly explain why the item is not applicable/relevant for your study

"A total of 374 individuals who were diagnosed with MDD through the Mini-International Neuropsychiatric Interview (M.I.N.I)[31] were enrolled in this trial between August 2021 and June 2023 from the Department of Depressive Disorder of Shenzhen Kangning Hospital and the Department of Psychiatry of Shenzhen Nanshan Center for Chronic Disease Control. Of the recruited participants, 317 were eligible for this trial."

4b-i) Report if outcomes were (self-)assessed through online questionnaires

Clearly report if outcomes were (self-)assessed through online questionnaires (as common in web-based trials) or otherwise.

	1	2	3	4	5	
subitem not at all important	○	○	○	○	○	essential

Does your paper address subitem 4b-i? *

Copy and paste relevant sections from the manuscript (include quotes in quotation marks "like this" to indicate direct quotes from your manuscript), or elaborate on this item by providing additional information not in the ms, or briefly explain why the item is not applicable/relevant for your study

"Eligible participants were then instructed to complete baseline assessments through online questionnaires. "

4b-ii) Report how institutional affiliations are displayed

Report how institutional affiliations are displayed to potential participants [on ehealth media], as affiliations with prestigious hospitals or universities may affect volunteer rates, use, and reactions with regards to an intervention.(Not a required item – describe only if this may bias results)

	1	2	3	4	5	
subitem not at all important	○	○	○	○	○	essential

Does your paper address subitem 4b-ii?

Copy and paste relevant sections from the manuscript (include quotes in quotation marks "like this" to indicate direct quotes from your manuscript), or elaborate on this item by providing additional information not in the ms, or briefly explain why the item is not applicable/relevant for your study

"A total of 374 individuals who were diagnosed with MDD through the Mini-International Neuropsychiatric Interview (M.I.N.I)[31] were enrolled in this trial between August 2021 and June 2023 from the Department of Depressive Disorder of Shenzhen Kangning Hospital and the Department of Psychiatry of Shenzhen Nanshan Center for Chronic Disease Control."

5) The interventions for each group with sufficient details to allow replication, including how and when they were actually administered

5-i) Mention names, credential, affiliations of the developers, sponsors, and owners

Mention names, credential, affiliations of the developers, sponsors, and owners [6] (if authors/evaluators are owners or developer of the software, this needs to be declared in a “Conflict of interest” section or mentioned elsewhere in the manuscript).

	1	2	3	4	5	
subitem not at all important	○	○	○	○	○	essential

Does your paper address subitem 5-i?

Copy and paste relevant sections from the manuscript (include quotes in quotation marks "like this" to indicate direct quotes from your manuscript), or elaborate on this item by providing additional information not in the ms, or briefly explain why the item is not applicable/relevant for your study

"The ICBT intervention in this trial was tailored to the Chinese population aiming at teaching practical CBT skills for depression. The self-developed ICBT course was designed as a Mini Program called Morning Mood on WeChat, one of the most popular messaging apps in China. In total, there were 7 lessons, covering psychoeducation, behavioral activation, cognitive restructuring, affect regulation, and relapse prevention. The detailed content of each lesson was demonstrated in another publication.[32]Participants in the ICBT group were required to complete one lesson per week; one week after completing the prior lesson, the subsequent lesson would be open automatically. This setting was used to prevent participants from completing multiple lessons in a short period, which might potentially impact the effectiveness. General support involving technical support and daily reminders provided by non-specialists (e.g., nurses, lay health workers, and social workers) via telephone or WeChat were applied to prompt engagement.
Participants in the WLC group were treated with usual care and no additional care was offered during the 8-week study period. After completing the post-treatment assessment, participants in the WLC group were asked whether they were willing to receive ICBT intervention at no cost. The login accounts and passwords for Morning Mood were then provided for the intended individuals."

5-ii) Describe the history/development process

Describe the history/development process of the application and previous formative evaluations (e.g., focus groups, usability testing), as these will have an impact on adoption/use rates and help with interpreting results.

	1	2	3	4	5	
subitem not at all important	O	O	O	O	O	essential

Does your paper address subitem 5-ii?

Copy and paste relevant sections from the manuscript (include quotes in quotation marks "like this" to indicate direct quotes from your manuscript), or elaborate on this item by providing additional information not in the ms, or briefly explain why the item is not applicable/relevant for your study

No, we did not describe the development process.

5-iii) Revisions and updating

Revisions and updating. Clearly mention the date and/or version number of the application/intervention (and comparator, if applicable) evaluated, or describe whether the intervention underwent major changes during the evaluation process, or whether the development and/or content was “frozen” during the trial. Describe dynamic components such as news feeds or changing content which may have an impact on the replicability of the intervention (for unexpected events see item 3b).

	1	2	3	4	5	
subitem not at all important	O	O	O	O	O	essential

Does your paper address subitem 5-iii?

Copy and paste relevant sections from the manuscript (include quotes in quotation marks "like this" to indicate direct quotes from your manuscript), or elaborate on this item by providing additional information not in the ms, or briefly explain why the item is not applicable/relevant for your study

No revisions or updatings

5-iv) Quality assurance methods

Provide information on quality assurance methods to ensure accuracy and quality of information provided [1], if applicable.

	1	2	3	4	5	
subitem not at all important	○	○	○	○	○	essential

Does your paper address subitem 5-iv?

Copy and paste relevant sections from the manuscript (include quotes in quotation marks "like this" to indicate direct quotes from your manuscript), or elaborate on this item by providing additional information not in the ms, or briefly explain why the item is not applicable/relevant for your study

"Participants in the ICBT group were required to complete one lesson per week; one week after completing the prior lesson, the subsequent lesson would be open automatically. This setting was used to prevent participants from completing multiple lessons in a short period, which might potentially impact the effectiveness. "

5-v) Ensure replicability by publishing the source code, and/or providing screenshots/screen-capture video, and/or providing flowcharts of the algorithms used

Ensure replicability by publishing the source code, and/or providing screenshots/screen-capture video, and/or providing flowcharts of the algorithms used. Replicability (i.e., other researchers should in principle be able to replicate the study) is a hallmark of scientific reporting.

	1	2	3	4	5	
subitem not at all important	○	○	○	○	○	essential

Does your paper address subitem 5-v?

Copy and paste relevant sections from the manuscript (include quotes in quotation marks "like this" to indicate direct quotes from your manuscript), or elaborate on this item by providing additional information not in the ms, or briefly explain why the item is not applicable/relevant for your study

We have mentioned the code availabitity.

5-vi) Digital preservation

Digital preservation: Provide the URL of the application, but as the intervention is likely to change or disappear over the course of the years; also make sure the intervention is archived (Internet Archive, webcitation.org, and/or publishing the source code or screenshots/videos alongside the article). As pages behind login screens cannot be archived, consider creating demo pages which are accessible without login.

	1	2	3	4	5	
subitem not at all important	○	○	○	○	○	essential

Does your paper address subitem 5-vi?

Copy and paste relevant sections from the manuscript (include quotes in quotation marks "like this" to indicate direct quotes from your manuscript), or elaborate on this item by providing additional information not in the ms, or briefly explain why the item is not applicable/relevant for your study

The course is not open, and the content and screenshots have been provided in a previous published paper.

5-vii) Access

Access: Describe how participants accessed the application, in what setting/context, if they had to pay (or were paid) or not, whether they had to be a member of specific group. If known, describe how participants obtained “access to the platform and Internet” [1]. To ensure access for editors/reviewers/readers, consider to provide a “backdoor” login account or demo mode for reviewers/readers to explore the application (also important for archiving purposes, see vi).

	1	2	3	4	5	
subitem not at all important	○	○	○	○	○	essential

Does your paper address subitem 5-vii? *

Copy and paste relevant sections from the manuscript (include quotes in quotation marks "like this" to indicate direct quotes from your manuscript), or elaborate on this item by providing additional information not in the ms, or briefly explain why the item is not applicable/relevant for your study

The course is not open, and the content and screenshots have been provided in a previous published paper.

5-viii) Mode of delivery, features/functionalities/components of the intervention and comparator, and the theoretical framework

Describe mode of delivery, features/functionalities/components of the intervention and comparator, and the theoretical framework [6] used to design them (instructional strategy [1], behaviour change techniques, persuasive features, etc., see e.g., [7, 8] for terminology). This includes an in-depth description of the content (including where it is coming from and who developed it) [1],” whether [and how] it is tailored to individual circumstances and allows users to track their progress and receive feedback” [6]. This also includes a description of communication delivery channels and – if computer-mediated communication is a component – whether communication was synchronous or asynchronous [6]. It also includes information on presentation strategies [1], including page design principles, average amount of text on pages, presence of hyperlinks to other resources, etc. [1].

	1	2	3	4	5	
subitem not at all important	○	○	○	○	○	essential

Does your paper address subitem 5-viii? *

Copy and paste relevant sections from the manuscript (include quotes in quotation marks "like this" to indicate direct quotes from your manuscript), or elaborate on this item by providing additional information not in the ms, or briefly explain why the item is not applicable/relevant for your study

"The ICBT intervention in this trial was tailored to the Chinese population aiming at teaching practical CBT skills for depression. The self-developed ICBT course was designed as a Mini Program called Morning Mood on WeChat, one of the most popular messaging apps in China. In total, there were 7 lessons, covering psychoeducation, behavioral activation, cognitive restructuring, affect regulation, and relapse prevention. The detailed content of each lesson was demonstrated in another publication.[32]Participants in the ICBT group were required to complete one lesson per week; one week after completing the prior lesson, the subsequent lesson would be open automatically. This setting was used to prevent participants from completing multiple lessons in a short period, which might potentially impact the effectiveness. General support involving technical support and daily reminders provided by non-specialists (e.g., nurses, lay health workers, and social workers) via telephone or WeChat were applied to prompt engagement."

5-ix) Describe use parameters

Describe use parameters (e.g., intended “doses” and optimal timing for use). Clarify what instructions or recommendations were given to the user, e.g., regarding timing, frequency, heaviness of use, if any, or was the intervention used ad libitum.

	1	2	3	4	5	
subitem not at all important	○	○	○	○	○	essential

Does your paper address subitem 5-ix?

Copy and paste relevant sections from the manuscript (include quotes in quotation marks "like this" to indicate direct quotes from your manuscript), or elaborate on this item by providing additional information not in the ms, or briefly explain why the item is not applicable/relevant for your study

"Participants in the ICBT group were required to complete one lesson per week; one week after completing the prior lesson, the subsequent lesson would be open automatically. "

5-x) Clarify the level of human involvement

Clarify the level of human involvement (care providers or health professionals, also technical assistance) in the e-intervention or as co-intervention (detail number and expertise of professionals involved, if any, as well as “type of assistance offered, the timing and frequency of the support, how it is initiated, and the medium by which the assistance is delivered”. It may be necessary to distinguish between the level of human involvement required for the trial, and the level of human involvement required for a routine application outside of a RCT setting (discuss under item 21 – generalizability).

	1	2	3	4	5	
subitem not at all important	○	○	○	○	○	essential

Does your paper address subitem 5-x?

Copy and paste relevant sections from the manuscript (include quotes in quotation marks "like this" to indicate direct quotes from your manuscript), or elaborate on this item by providing additional information not in the ms, or briefly explain why the item is not applicable/relevant for your study

" General support involving technical support and daily reminders provided by non-specialists (e.g., nurses, lay health workers, and social workers) via telephone or WeChat were applied to prompt engagement."

5-xi) Report any prompts/reminders used

Report any prompts/reminders used: Clarify if there were prompts (letters, emails, phone calls, SMS) to use the application, what triggered them, frequency etc. It may be necessary to distinguish between the level of prompts/reminders required for the trial, and the level of prompts/reminders for a routine application outside of a RCT setting (discuss under item 21 – generalizability).

	1	2	3	4	5	
subitem not at all important	○	○	○	○	○	essential

Does your paper address subitem 5-xi? *

Copy and paste relevant sections from the manuscript (include quotes in quotation marks "like this" to indicate direct quotes from your manuscript), or elaborate on this item by providing additional information not in the ms, or briefly explain why the item is not applicable/relevant for your study

" General support involving technical support and daily reminders provided by non-specialists (e.g., nurses, lay health workers, and social workers) via telephone or WeChat were applied to prompt engagement."

5-xii) Describe any co-interventions (incl. training/support)

Describe any co-interventions (incl. training/support): Clearly state any interventions that are provided in addition to the targeted eHealth intervention, as ehealth intervention may not be designed as stand-alone intervention. This includes training sessions and support [1]. It may be necessary to distinguish between the level of training required for the trial, and the level of training for a routine application outside of a RCT setting (discuss under item 21 – generalizability.

	1	2	3	4	5	
subitem not at all important	○	○	○	○	○	essential

Does your paper address subitem 5-xii? *

Copy and paste relevant sections from the manuscript (include quotes in quotation marks "like this" to indicate direct quotes from your manuscript), or elaborate on this item by providing additional information not in the ms, or briefly explain why the item is not applicable/relevant for your study

No co-intervention was adopted in this trial.

6a) Completely defined pre-specified primary and secondary outcome measures, including how and when they were assessed

Does your paper address CONSORT subitem 6a? *

Copy and paste relevant sections from the manuscript (include quotes in quotation marks "like this" to indicate direct quotes from your manuscript), or elaborate on this item by providing additional information not in the ms, or briefly explain why the item is not applicable/relevant for your study

"The primary outcome was depressive symptoms measured by the Chinese version of PHQ-9, which was with good reliability and validity.[33]Its Cronbach's α was 0.91 in this study. Remission was defined as a PHQ-9 score less than 10, where the threshold was set and adopted by the Improving Access to Psychological Therapies of the United Kingdom to distinguish clinical cases and non-cases.[34,35]Response was defined as symptom improvement (i.e., the reduction of PHQ-9 score) of 50% or more.[13]
The secondary outcome measures included anxiety symptoms, non-specific psychological distress, social function impairment, general self-efficacy, quality of life, and depression stigma, which were assessed by the Chinese version of the Generalized Anxiety Disorder 7-Item (GAD-7), the Kessler's Psychological Distress Scale 10-item (K-10), the Sheehan Disability Scale (SDS), the General Self-Efficacy Scale (GSES), the Short Form 6-Dimension (SF-6D) and Depression Stigma Scale (DSS), respectively. These measurement tools have good reliability and validity.[36–41]In this study, the corresponding Cronbach's α was 0.94, 0.96, 0.94, 0.71, 0.95, and 0.84, respectively. All outcomes were evaluated at pre- and post-treatment, with further follow-up at 3, 6, and 12 months after intervention in the intervention arm."

6a-i) Online questionnaires: describe if they were validated for online use and apply CHERRIES items to describe how the questionnaires were designed/deployed

If outcomes were obtained through online questionnaires, describe if they were validated for online use and apply CHERRIES items to describe how the questionnaires were designed/deployed [9].

	1	2	3	4	5	
subitem not at all important	○	○	○	○	○	essential

Does your paper address subitem 6a-i?

Copy and paste relevant sections from manuscript text

"Eligible participants were then instructed to complete baseline and follow-up assessments through online questionnaires."

6a-ii) Describe whether and how “use” (including intensity of use/dosage) was defined/measured/monitored

Describe whether and how “use” (including intensity of use/dosage) was defined/measured/monitored (logins, logfile analysis, etc.). Use/adoption metrics are important process outcomes that should be reported in any ehealth trial.

	1	2	3	4	5	
subitem not at all important	○	○	○	○	○	essential

Does your paper address subitem 6a-ii?

Copy and paste relevant sections from manuscript text

In this trial, the analysis followed the intention-to-treatment principle. The usage information was not considered.

6a-iii) Describe whether, how, and when qualitative feedback from participants was obtained

Describe whether, how, and when qualitative feedback from participants was obtained (e.g., through emails, feedback forms, interviews, focus groups).

	1	2	3	4	5	
subitem not at all important	○	○	○	○	○	essential

Does your paper address subitem 6a-iii?

Copy and paste relevant sections from manuscript text

"Participants’ satisfaction and acceptability for ICBT treatment were investigated post-treatment by the following three questions: (1) “Do you think the courses in this project have been helpful for you”; (2) “Are you satisfied with the courses in this project”; and (3) “Would you be willing to recommend this project to people in need”."

6b) Any changes to trial outcomes after the trial commenced, with reasons

Does your paper address CONSORT subitem 6b? *

Copy and paste relevant sections from the manuscript (include quotes in quotation marks "like this" to indicate direct quotes from your manuscript), or elaborate on this item by providing additional information not in the ms, or briefly explain why the item is not applicable/relevant for your study

No change to trial outcomes after the trial commenced.

7a) How sample size was determined

NPT: When applicable, details of whether and how the clustering by care provides or centers was addressed

7a-i) Describe whether and how expected attrition was taken into account when calculating the sample size

Describe whether and how expected attrition was taken into account when calculating the sample size.

	1	2	3	4	5	
subitem not at all important	○	○	○	○	○	essential

Does your paper address subitem 7a-i?

Copy and paste relevant sections from manuscript title (include quotes in quotation marks "like this" to indicate direct quotes from your manuscript), or elaborate on this item by providing additional information not in the ms, or briefly explain why the item is not applicable/relevant for your study

"The sample size was determined by a priori power analysis. A total of 86 participants per group (N=172) was thought to be sufficient to detect a moderate between-group effect size (Cohen's d=0.50, defined as the standardized difference between groups obtained by calculating the mean difference and dividing by the pooled standard deviation) on repeated measures for PHQ-9, with a power of 90% and two-tailed α of 0.05. The expected sample size was calculated based on a previous meta-analysis of individual patient data that investigated the effectiveness of unguided ICBT versus inactive controls for depressive symptoms[13]. Taking the attrition into consideration, the target sample size should be 228 patients when the anticipated dropout rate was set at 25%.[42] "

7b) When applicable, explanation of any interim analyses and stopping guidelines

Does your paper address CONSORT subitem 7b? *

Copy and paste relevant sections from the manuscript (include quotes in quotation marks "like this" to indicate direct quotes from your manuscript), or elaborate on this item by providing additional information not in the ms, or briefly explain why the item is not applicable/relevant for your study

No interim analyses and stopping guidelines.

8a) Method used to generate the random allocation sequence
NPT: When applicable, how care providers were allocated to each trial group

Does your paper address CONSORT subitem 8a? *

Copy and paste relevant sections from the manuscript (include quotes in quotation marks "like this" to indicate direct quotes from your manuscript), or elaborate on this item by providing additional information not in the ms, or briefly explain why the item is not applicable/relevant for your study

"The randomization was applied by using an online random number generator, with a ratio of 1:1 in the ICBT and WLC groups. Due to the nature of the interventions, the blind method was hard to carry out."

8b) Type of randomisation; details of any restriction (such as blocking and block size)

Does your paper address CONSORT subitem 8b? *

Copy and paste relevant sections from the manuscript (include quotes in quotation marks "like this" to indicate direct quotes from your manuscript), or elaborate on this item by providing additional information not in the ms, or briefly explain why the item is not applicable/relevant for your study

simple randomisation was adopted in this trial.

9) Mechanism used to implement the random allocation sequence (such as sequentially numbered containers), describing any steps taken to conceal the sequence until interventions were assigned

Does your paper address CONSORT subitem 9? *

Copy and paste relevant sections from the manuscript (include quotes in quotation marks "like this" to indicate direct quotes from your manuscript), or elaborate on this item by providing additional information not in the ms, or briefly explain why the item is not applicable/relevant for your study

"The randomization was applied by using an online random number generator, with a ratio of 1:1 in the ICBT and WLC groups. Due to the nature of the interventions, the blind method was hard to carry out."

10) Who generated the random allocation sequence, who enrolled participants, and who assigned participants to interventions

Does your paper address CONSORT subitem 10? *

Copy and paste relevant sections from the manuscript (include quotes in quotation marks "like this" to indicate direct quotes from your manuscript), or elaborate on this item by providing additional information not in the ms, or briefly explain why the item is not applicable/relevant for your study

您的回答

11a) If done, who was blinded after assignment to interventions (for example, participants, care providers, those assessing outcomes) and how

NPT: Whether or not administering co-interventions were blinded to group assignment

11a-i) Specify who was blinded, and who wasn't

Specify who was blinded, and who wasn't. Usually, in web-based trials it is not possible to blind the participants [1, 3] (this should be clearly acknowledged), but it may be possible to blind outcome assessors, those doing data analysis or those administering co-interventions (if any).

	1	2	3	4	5	
subitem not at all important	O	O	O	O	O	essential

Does your paper address subitem 11a-i? *

Copy and paste relevant sections from the manuscript (include quotes in quotation marks "like this" to indicate direct quotes from your manuscript), or elaborate on this item by providing additional information not in the ms, or briefly explain why the item is not applicable/relevant for your study

"The randomization was applied by using an online random number generator, with a ratio of 1:1 in the ICBT and WLC groups. Due to the nature of the interventions, the blind method was hard to carry out."

11a-ii) Discuss e.g., whether participants knew which intervention was the "intervention of interest" and which one was the "comparator"

Informed consent procedures (4a-ii) can create biases and certain expectations - discuss e.g., whether participants knew which intervention was the "intervention of interest" and which one was the "comparator".

	1	2	3	4	5	
subitem not at all important	O	O	O	O	O	essential

Does your paper address subitem 11a-ii?

Copy and paste relevant sections from the manuscript (include quotes in quotation marks "like this" to indicate direct quotes from your manuscript), or elaborate on this item by providing additional information not in the ms, or briefly explain why the item is not applicable/relevant for your study

"Discuss e.g., whether participants knew which intervention was the “intervention of interest” and which one was the “comparator”"

11b) If relevant, description of the similarity of interventions

(this item is usually not relevant for ehealth trials as it refers to similarity of a placebo or sham intervention to a active medication/intervention)

Does your paper address CONSORT subitem 11b? *

Copy and paste relevant sections from the manuscript (include quotes in quotation marks "like this" to indicate direct quotes from your manuscript), or elaborate on this item by providing additional information not in the ms, or briefly explain why the item is not applicable/relevant for your study

您的回答

12a) Statistical methods used to compare groups for primary and secondary outcomes

NPT: When applicable, details of whether and how the clustering by care providers or centers was addressed

Does your paper address CONSORT subitem 12a? *

Copy and paste relevant sections from the manuscript (include quotes in quotation marks "like this" to indicate direct quotes from your manuscript), or elaborate on this item by providing additional information not in the ms, or briefly explain why the item is not applicable/relevant for your study

"Linear mixed models (LMM) and marginal models for repeated measures (MMRM) were applied to evaluate the effectiveness of intervention by estimating mean between-group differences and mean changes for each group at each time point. Both statistical and graphical checks were conducted to assess model assumptions. In the LMM model, treatment groups, the time point of measurement, and the treatment by time point interaction were treated as fixed effects and the individual patient effect was set as a random effect with normal distribution and expected value 0. In the MMRM model, an unstructured correlation structure was used for model fit. There were 18 models, one up to 8-week across arms ("8-week model") and one including combined intervention group data ("follow-up model") per outcome variable (depressive symptoms, anxiety symptoms, psychological distress, social function impairment, health-related quality of life, general self-efficacy, depression stigma, personal depression stigma, and perceived depression stigma). Model fit was evaluated using Schwarz's Bayesian criterion. Within-group and between-group effect sizes (Cohen's d) were calculated based on the method suggested for LMM analysis.[43,44]It was reported that the effect be categorized as small (>0.20), moderate (>0.50), and large effect (>0.80) according to Cohen's d.[45]Sensitivity analyses for long-term effectiveness evaluation were conducted respectively among participants treated with ICBT in the ICBT group and WLC group.
The remission and response rates in the ICBT and WLC groups were also estimated by treatment arms (ICBT vs WLC) and time points (post-treatment, 3-,6-, and 12-month follow-ups). Chi-squared tests were performed to assess the frequency differences in remission and response between the 2 groups. All analyses were performed using R language 4.2.2, and a P-value less than 0.05 was considered statistically significant.
"

12a-i) Imputation techniques to deal with attrition / missing values

Imputation techniques to deal with attrition / missing values: Not all participants will use the intervention/comparator as intended and attrition is typically high in ehealth trials. Specify how participants who did not use the application or dropped out from the trial were treated in the statistical analysis (a complete case analysis is strongly discouraged, and simple imputation techniques such as LOCF may also be problematic [4]).

	1	2	3	4	5	
subitem not at all important	○	○	○	○	○	essential

Does your paper address subitem 12a-i? *

Copy and paste relevant sections from the manuscript (include quotes in quotation marks "like this" to indicate direct quotes from your manuscript), or elaborate on this item by providing additional information not in the ms, or briefly explain why the item is not applicable/relevant for your study

No imputation technique was adopted.

12b) Methods for additional analyses, such as subgroup analyses and adjusted analyses

Does your paper address CONSORT subitem 12b? *

Copy and paste relevant sections from the manuscript (include quotes in quotation marks "like this" to indicate direct quotes from your manuscript), or elaborate on this item by providing additional information not in the ms, or briefly explain why the item is not applicable/relevant for your study

"Sensitivity analyses for long-term effectiveness evaluation were conducted respectively among participants treated with ICBT in the ICBT group and WLC group."

X26) REB/IRB Approval and Ethical Considerations [recommended as subheading under "Methods"] (not a CONSORT item)

X26-i) Comment on ethics committee approval

	1	2	3	4	5	
subitem not at all important	○	○	○	○	○	essential

Does your paper address subitem X26-i?

Copy and paste relevant sections from the manuscript (include quotes in quotation marks "like this" to indicate direct quotes from your manuscript), or elaborate on this item by providing additional information not in the ms, or briefly explain why the item is not applicable/relevant for your study

"This trial was registered with the Chinese Clinical Trial Registry (registration number ChiCTR2100046425). The study protocol, patient information sheets, and informed consent forms were approved by the Medical Ethics Committee of Shenzhen Nanshan Center for Chronic Disease Control, China (ethical approval number ll20210012). The Consolidated Standards of Reporting Trials (CONSORT) with the eHealth extension was adopted to guide the trial.[30]All participants provided informed consent."

x26-ii) Outline informed consent procedures

Outline informed consent procedures e.g., if consent was obtained offline or online (how? Checkbox, etc.?), and what information was provided (see 4a-ii). See [6] for some items to be included in informed consent documents.

	1	2	3	4	5	
subitem not at all important	○	○	○	○	○	essential

Does your paper address subitem X26-ii?

Copy and paste relevant sections from the manuscript (include quotes in quotation marks "like this" to indicate direct quotes from your manuscript), or elaborate on this item by providing additional information not in the ms, or briefly explain why the item is not applicable/relevant for your study

”All participants provided informed consent.“

X26-iii) Safety and security procedures

Safety and security procedures, incl. privacy considerations, and any steps taken to reduce the likelihood or detection of harm (e.g., education and training, availability of a hotline)

	1	2	3	4	5	
subitem not at all important	○	○	○	○	○	essential

Does your paper address subitem X26-iii?

Copy and paste relevant sections from the manuscript (include quotes in quotation marks "like this" to indicate direct quotes from your manuscript), or elaborate on this item by providing additional information not in the ms, or briefly explain why the item is not applicable/relevant for your study

您的回答

RESULTS

13a) For each group, the numbers of participants who were randomly assigned, received intended treatment, and were analysed for the primary outcome

NPT: The number of care providers or centers performing the intervention in each group and the number of patients treated by each care provider in each center

Does your paper address CONSORT subitem 13a? *

Copy and paste relevant sections from the manuscript (include quotes in quotation marks "like this" to indicate direct quotes from your manuscript), or elaborate on this item by providing additional information not in the ms, or briefly explain why the item is not applicable/relevant for your study

”A total of 374 participants with MDD were recruited for this trial between August 2021 and June 2023, of whom 317 cases entered the trial and were randomized (ICBT: N=159; WLC: N=158). “

13b) For each group, losses and exclusions after randomisation, together with reasons

Does your paper address CONSORT subitem 13b? (NOTE: Preferably, this is shown in a CONSORT flow diagram) *

Copy and paste relevant sections from the manuscript (include quotes in quotation marks "like this" to indicate direct quotes from your manuscript), or elaborate on this item by providing additional information not in the ms, or briefly explain why the item is not applicable/relevant for your study

" In the ICBT group, 7 participants did not start Lesson 1 and thus were excluded. The treatment completion rate was 79.6% (121/152) and the attrition rate at 12-month follow-up was 35.5% (54/121). The average number of ICBT lessons completed was 5.89 (SD=2.13). In the WLC group, there were 10 patients of which contact was lost or withdrew from the trial after randomization. The dropout rate was 6.3% (10/158). "

13b-i) Attrition diagram

Strongly recommended: An attrition diagram (e.g., proportion of participants still logging in or using the intervention/comparator in each group plotted over time, similar to a survival curve) or other figures or tables demonstrating usage/dose/engagement.

	1	2	3	4	5	
subitem not at all important	○	○	○	○	○	essential

Does your paper address subitem 13b-i?

Copy and paste relevant sections from the manuscript or cite the figure number if applicable (include quotes in quotation marks "like this" to indicate direct quotes from your manuscript), or elaborate on this item by providing additional information not in the ms, or briefly explain why the item is not applicable/relevant for your study

"The detailed completion and dropout information are presented in Figure 1."

14a) Dates defining the periods of recruitment and follow-up

Does your paper address CONSORT subitem 14a? *

Copy and paste relevant sections from the manuscript (include quotes in quotation marks "like this" to indicate direct quotes from your manuscript), or elaborate on this item by providing additional information not in the ms, or briefly explain why the item is not applicable/relevant for your study

“A total of 374 participants with MDD were recruited for this trial between August 2021 and June 2023, of whom 317 cases entered the trial and were randomized (ICBT: N=159; WLC: N=158).”

14a-i) Indicate if critical “secular events” fell into the study period

Indicate if critical “secular events” fell into the study period, e.g., significant changes in Internet resources available or “changes in computer hardware or Internet delivery resources”

	1	2	3	4	5	
subitem not at all important	○	○	○	○	○	essential

Does your paper address subitem 14a-i?

Copy and paste relevant sections from the manuscript (include quotes in quotation marks "like this" to indicate direct quotes from your manuscript), or elaborate on this item by providing additional information not in the ms, or briefly explain why the item is not applicable/relevant for your study

您的回答

14b) Why the trial ended or was stopped (early)

Does your paper address CONSORT subitem 14b? *

Copy and paste relevant sections from the manuscript (include quotes in quotation marks "like this" to indicate direct quotes from your manuscript), or elaborate on this item by providing additional information not in the ms, or briefly explain why the item is not applicable/relevant for your study

The study ended with as scheduled

15) A table showing baseline demographic and clinical characteristics for each group

NPT: When applicable, a description of care providers (case volume, qualification, expertise, etc.) and centers (volume) in each group

Does your paper address CONSORT subitem 15? *

Copy and paste relevant sections from the manuscript (include quotes in quotation marks "like this" to indicate direct quotes from your manuscript), or elaborate on this item by providing additional information not in the ms, or briefly explain why the item is not applicable/relevant for your study

"Table 1 outlines the baseline characteristics of the participants."

15-i) Report demographics associated with digital divide issues

In ehealth trials it is particularly important to report demographics associated with digital divide issues, such as age, education, gender, social-economic status, computer/Internet/ehealth literacy of the participants, if known.

	1	2	3	4	5	
subitem not at all important	○	○	○	○	○	essential

Does your paper address subitem 15-i? *

Copy and paste relevant sections from the manuscript (include quotes in quotation marks "like this" to indicate direct quotes from your manuscript), or elaborate on this item by providing additional information not in the ms, or briefly explain why the item is not applicable/relevant for your study

"The mean age was 28.49 (SD=7.00) years and the female-to-male ratio was 3:1. Most participants were Han ethnic (95.0%), highly educated (82.6%), employed (64.6%) and non-married (70.5%). Participants whose monthly household income was below 10,000 Chinese Yuan (CNY) accounted for the largest part (34.2%). In modifiable lifestyle behaviors, the majority of the participants did not smoke cigarettes (62.2%), lived with other people (74.2%), drank alcohol (83.3%), and had no exercise habits (72.9%). In terms of clinical features, more than half of the participants were at their first episodes of MDD (59.9%), did not take antidepressants (54.3%), or did not have comorbidities at baseline (63.8%). The mean (SD) of baseline outcome measures including PHQ-9, GAD-7, K-10, SDS, SF-6D, GSES, and DSS were 13.40(4.83), 10.25(4.60), 29.73 (7.48), 13.81(6.81), 0.56(0.21), 19.61(5.71) and 52.08(9.14), respectively. Baseline characteristics were comparable between the ICBT and WLC groups, suggesting that the randomization was successful."

16) For each group, number of participants (denominator) included in each analysis and whether the analysis was by original assigned groups

16-i) Report multiple “denominators” and provide definitions

Report multiple “denominators” and provide definitions: Report N’s (and effect sizes) “across a range of study participation [and use] thresholds” [1], e.g., N exposed, N consented, N used more than x times, N used more than y weeks, N participants “used” the intervention/comparator at specific pre-defined time points of interest (in absolute and relative numbers per group). Always clearly define “use” of the intervention.

	1	2	3	4	5	
subitem not at all important	○	○	○	○	○	essential

Does your paper address subitem 16-i? *

Copy and paste relevant sections from the manuscript (include quotes in quotation marks "like this" to indicate direct quotes from your manuscript), or elaborate on this item by providing additional information not in the ms, or briefly explain why the item is not applicable/relevant for your study

"The ITT sample used for the short-term effectiveness analyses consisted of 152 patients in the ICBT group and 148 patients in the WLC group. For the long-term effectiveness evaluation, a total of 260 participants receiving ICBT intervention in the two groups were included."

16-ii) Primary analysis should be intent-to-treat

Primary analysis should be intent-to-treat, secondary analyses could include comparing only "users", with the appropriate caveats that this is no longer a randomized sample (see 18-i).

	1	2	3	4	5	
subitem not at all important	○	○	○	○	○	essential

Does your paper address subitem 16-ii?

Copy and paste relevant sections from the manuscript (include quotes in quotation marks "like this" to indicate direct quotes from your manuscript), or elaborate on this item by providing additional information not in the ms, or briefly explain why the item is not applicable/relevant for your study

"8-week models suggested significant interaction effects of time-by-intervention-arm for PHQ-9 (β=-3.65, P<0.001), GAD-7 (β=-2.85, P<0.001), K-10 (β=-4.87, P<0.001), SDS (β=-5.57, P<0.001), GSES (β=1.42, P=0.011), DSS (β =-2.93, P=0.028), and personal DSS (β=-1.80, P=0.008), except for SF-6D (β=0.01, P=0.497) and perceived DSS (β=-1.21, P=0.206)(See Table S1). "

17a) For each primary and secondary outcome, results for each group, and the estimated effect size and its precision (such as 95% confidence interval)

Does your paper address CONSORT subitem 17a? *

Copy and paste relevant sections from the manuscript (include quotes in quotation marks "like this" to indicate direct quotes from your manuscript), or elaborate on this item by providing additional information not in the ms, or briefly explain why the item is not applicable/relevant for your study

"Figure 2 illustrates the change in estimated outcome measures across time by treatment arms. In both treatments, the scores of PHQ-9, GAD-7, and K-10 decreased over time, while the scores of SF-6D, GSES, and perceived DSS increased after treatment. Compared with the WLC group, larger improvements were obtained in these outcomes for those who received ICBT treatment. The changes in SDS, DSS, and personal DSS of the two groups moved in opposite directions, with a reduction in social function impairment, depression stigma, and personal stigma showing in the ICBT group.
The within- and between-group effect sizes measured by Cohen's d were calculated based on the estimated means (see Table S2). For depressive symptoms, the between-group effect size was moderate (d = 0.50, 95% CI: 0.26-0.75). The within-group effect size was large for the ICBT group (d =1.02, 95% CI:0.76-1.27), while in the WLC group, the effect size was small (d =0.34, 95% CI: 0.11-0.57). For secondary outcomes, the between-group effect sizes were small (d range: 0.07–0.43). The within-group effect sizes in the ICBT group ranged from small to large, with GAD-7 (d = 0.89, 95% CI: 0.63, 1.14) and K-10 (d = 0.87, 95% CI: 0.61, 1.12) exhibiting large sizes, SDS (d = 0.58, 95% CI: 0.33, 0.83) showing moderate size and the left displaying small sizes (d range: 0.01–0.39). The within-group effect sizes in the WLC group were small, with a d range of 0.10 to 0.31.
"

17a-i) Presentation of process outcomes such as metrics of use and intensity of use

In addition to primary/secondary (clinical) outcomes, the presentation of process outcomes such as metrics of use and intensity of use (dose, exposure) and their operational definitions is critical. This does not only refer to metrics of attrition (13-b) (often a binary variable), but also to more continuous exposure metrics such as "average session length". These must be accompanied by a technical description how a metric like a "session" is defined (e.g., timeout after idle time) [1] (report under item 6a).

	1	2	3	4	5	
subitem not at all important	○	○	○	○	○	essential

Does your paper address subitem 17a-i?

Copy and paste relevant sections from the manuscript (include quotes in quotation marks "like this" to indicate direct quotes from your manuscript), or elaborate on this item by providing additional information not in the ms, or briefly explain why the item is not applicable/relevant for your study

No process outcomes such as metrics of use and intensity of use.

17b) For binary outcomes, presentation of both absolute and relative effect sizes is recommended

Does your paper address CONSORT subitem 17b? *

Copy and paste relevant sections from the manuscript (include quotes in quotation marks "like this" to indicate direct quotes from your manuscript), or elaborate on this item by providing additional information not in the ms, or briefly explain why the item is not applicable/relevant for your study

"At post-treatment, a total of 80 complete remissions and 50 responders were observed in 152 patients who received ICBT treatment. The remission and response rates respectively reached 66.1% and 41.3%. The clinical symptomatic remissions in the WLC group were significantly lower than that of the ICBT group, with complete remissions in 58 cases (39.2%, P<0.001) and response in 27 of the 148 patients (18.2%, P<0.001). During the follow-up period, the remission and response rates in the ICBT group remained high."

18) Results of any other analyses performed, including subgroup analyses and adjusted analyses, distinguishing pre-specified from exploratory

Does your paper address CONSORT subitem 18? *

Copy and paste relevant sections from the manuscript (include quotes in quotation marks "like this" to indicate direct quotes from your manuscript), or elaborate on this item by providing additional information not in the ms, or briefly explain why the item is not applicable/relevant for your study

"Paired comparisons confirmed no significant change from post-treatment to 12-month follow-up on PHQ-9 (Bonferroni adjusted estimated mean difference -0.81; P=0.334), GAD-7 (Bonferroni adjusted estimated mean difference -0.63; P=0.633), K-10 (Bonferroni adjusted estimated mean difference -1.11; P=0.644), SDS (Bonferroni adjusted estimated mean difference 0.88; P=0.330), GSES (Bonferroni adjusted estimated mean difference 0.12; P=0.998), DSS (Bonferroni adjusted estimated mean difference -0.49; P=0.954) and perceived DSS (Bonferroni adjusted estimated mean difference 0.58; P=0.736), while significant increases in SF-6D (Bonferroni adjusted estimated mean difference -0.039; P=0.044) and personal DSS (Bonferroni adjusted estimated mean difference -1.06; P=0.031) (see Table 3). "

18-i) Subgroup analysis of comparing only users

A subgroup analysis of comparing only users is not uncommon in ehealth trials, but if done, it must be stressed that this is a self-selected sample and no longer an unbiased sample from a randomized trial (see 16-iii).

	1	2	3	4	5	
subitem not at all important	○	○	○	○	○	essential

Does your paper address subitem 18-i?

Copy and paste relevant sections from the manuscript (include quotes in quotation marks "like this" to indicate direct quotes from your manuscript), or elaborate on this item by providing additional information not in the ms, or briefly explain why the item is not applicable/relevant for your study

"The results of the two sensitivity analyses were almost consistent with those of the main analysis (see Table S4–S7 and Figure S1-S2 in the Supplementary Materials). For personal DSS, both the main analysis and sensitivity analyses showed down first and then up, with only the main analysis reporting statistical significances."

19) All important harms or unintended effects in each group
(for specific guidance see CONSORT for harms)

Does your paper address CONSORT subitem 19? *

Copy and paste relevant sections from the manuscript (include quotes in quotation marks "like this" to indicate direct quotes from your manuscript), or elaborate on this item by providing additional information not in the ms, or briefly explain why the item is not applicable/relevant for your study

No harms or unintended effects in each group.

19-i) Include privacy breaches, technical problems

Include privacy breaches, technical problems. This does not only include physical “harm” to participants, but also incidents such as perceived or real privacy breaches [1], technical problems, and other unexpected/unintended incidents. “Unintended effects” also includes unintended positive effects [2].

	1	2	3	4	5	
subitem not at all important	○	○	○	○	○	essential

Does your paper address subitem 19-i?

Copy and paste relevant sections from the manuscript (include quotes in quotation marks "like this" to indicate direct quotes from your manuscript), or elaborate on this item by providing additional information not in the ms, or briefly explain why the item is not applicable/relevant for your study

No harms or unintended effects in each group.

19-ii) Include qualitative feedback from participants or observations from staff/researchers

Include qualitative feedback from participants or observations from staff/researchers, if available, on strengths and shortcomings of the application, especially if they point to unintended/unexpected effects or uses. This includes (if available) reasons for why people did or did not use the application as intended by the developers.

	1	2	3	4	5	
subitem not at all important	○	○	○	○	○	essential

Does your paper address subitem 19-ii?

Copy and paste relevant sections from the manuscript (include quotes in quotation marks "like this" to indicate direct quotes from your manuscript), or elaborate on this item by providing additional information not in the ms, or briefly explain why the item is not applicable/relevant for your study

"A total of 119 participants (85.6%) completed the course evaluation regarding perceived helpfulness, satisfaction, and recommended intention. 98 (82.4%) reported that the course was helpful, 117 (98.3%) reported feeling satisfied with the course, and 111(93.2%) participants were willing to recommend the course to others around them."

DISCUSSION

22) Interpretation consistent with results, balancing benefits and harms, and considering other relevant evidence

NPT: In addition, take into account the choice of the comparator, lack of or partial blinding, and unequal expertise of care providers or centers in each group

22-i) Restate study questions and summarize the answers suggested by the data, starting with primary outcomes and process outcomes (use)

Restate study questions and summarize the answers suggested by the data, starting with primary outcomes and process outcomes (use).

	1	2	3	4	5	
subitem not at all important	○	○	○	○	○	essential

Does your paper address subitem 22-i? *

Copy and paste relevant sections from the manuscript (include quotes in quotation marks "like this" to indicate direct quotes from your manuscript), or elaborate on this item by providing additional information not in the ms, or briefly explain why the item is not applicable/relevant for your study

"This study assessed the short- and long-term effectiveness of an unguided ICBT course in Chinese patients with MDD and evaluated participants' acceptability and satisfaction with ICBT. Compared to WLC, depressive symptoms, disease-related symptoms, individual and social functioning, and quality of life in the ICBT group were significantly improved at the post-treatment stage, with the between-group effect size of PHQ-9 reaching 0.50. Remission and response rates of the ICBT group were statistically higher than those of the WLC group (66.1% vs 39.2%, P<0.001; 41.3% vs 18.2%, P<0.001). Moreover, these positive results in the ICBT group were maintained after the intervention. At 12-month follow-up, the depressive symptoms were improved compared with that at pretreatment (mean difference -3.90; P<0.001; d=0.70) and no significant change was observed in comparison with the outcomes at posttreatment (mean difference -0.81; P=0.334; d=-0.15). Patients treated with ICBT reported high acceptability with the engagement rate of 79.6%. Above 90% of participants in the ICBT group reported high treatment satisfaction. To our knowledge, this was the first study that investigated both the short- and long-term effectiveness of unguided ICBT supported by non-specialists for MDD in China. "

22-ii) Highlight unanswered new questions, suggest future research

Highlight unanswered new questions, suggest future research.

	1	2	3	4	5	
subitem not at all important	○	○	○	○	○	essential

Does your paper address subitem 22-ii?

Copy and paste relevant sections from the manuscript (include quotes in quotation marks "like this" to indicate direct quotes from your manuscript), or elaborate on this item by providing additional information not in the ms, or briefly explain why the item is not applicable/relevant for your study

"More measures should be taken to enhance treatment outcome when putting Morning Mood into large-scale use. ""The long-term effect of ICBT on depressive symptoms and absolute treatment outcomes suggested that intensified measures might be needed to consolidate the desired therapeutic effects for the long term in our future application."

20) Trial limitations, addressing sources of potential bias, imprecision, and, if relevant, multiplicity of analyses

20-i) Typical limitations in ehealth trials

Typical limitations in ehealth trials: Participants in ehealth trials are rarely blinded. Ehealth trials often look at a multiplicity of outcomes, increasing risk for a Type I error. Discuss biases due to non-use of the intervention/usability issues, biases through informed consent procedures, unexpected events.

	1	2	3	4	5	
subitem not at all important	○	○	○	○	○	essential

Does your paper address subitem 20-i? *

Copy and paste relevant sections from the manuscript (include quotes in quotation marks "like this" to indicate direct quotes from your manuscript), or elaborate on this item by providing additional information not in the ms, or briefly explain why the item is not applicable/relevant for your study

"due to ethical considerations, participants in the WLC group are arranged to receive ICBT treatment after an 8-week intervention period. That is, no control exists during the follow-up phase. As a result, it is difficult to distinguish the effect of ICBT from usual care and the natural course. A further RCT with long-term follow-ups in all treatment arms should be conducted to solve this problem."

21) Generalisability (external validity, applicability) of the trial findings

NPT: External validity of the trial findings according to the intervention, comparators, patients, and care providers or centers involved in the trial

21-i) Generalizability to other populations

Generalizability to other populations: In particular, discuss generalizability to a general Internet population, outside of a RCT setting, and general patient population, including applicability of the study results for other organizations

	1	2	3	4	5	
subitem not at all important	○	○	○	○	○	essential

Does your paper address subitem 21-i?

Copy and paste relevant sections from the manuscript (include quotes in quotation marks "like this" to indicate direct quotes from your manuscript), or elaborate on this item by providing additional information not in the ms, or briefly explain why the item is not applicable/relevant for your study

"the participants in our study were recruited from mental health clinics in Shenzhen, China, and were diagnosed with MDD. The majority of them were female and received high levels of education. Therefore, the generalization of the present findings may be limited."

21-ii) Discuss if there were elements in the RCT that would be different in a routine application setting

Discuss if there were elements in the RCT that would be different in a routine application setting (e.g., prompts/reminders, more human involvement, training sessions or other co-interventions) and what impact the omission of these elements could have on use, adoption, or outcomes if the intervention is applied outside of a RCT setting.

	1	2	3	4	5	
subitem not at all important	○	○	○	○	○	essential

Does your paper address subitem 21-ii?

Copy and paste relevant sections from the manuscript (include quotes in quotation marks "like this" to indicate direct quotes from your manuscript), or elaborate on this item by providing additional information not in the ms, or briefly explain why the item is not applicable/relevant for your study

"in our study, the unguided ICBT intervention is supported by non-specialist health workers (i.e., any type of health worker, like a nurse, lay health worker, or medical social worker, who is not a specialist in mental health). This form of ICBT has no requirement for qualified mental health specialists and thus is easier to implement. The results of our study give a reference to solving the problem of the treatment gap in resource-limited countries like China"

OTHER INFORMATION

23) Registration number and name of trial registry

Does your paper address CONSORT subitem 23? *

Copy and paste relevant sections from the manuscript (include quotes in quotation marks "like this" to indicate direct quotes from your manuscript), or elaborate on this item by providing additional information not in the ms, or briefly explain why the item is not applicable/relevant for your study

"ChiCTR2100046425"

Your answer must have a minimum of 25 characters.

24) Where the full trial protocol can be accessed, if available

Does your paper address CONSORT subitem 24? *

Cite a Multimedia Appendix, other reference, or copy and paste relevant sections from the manuscript (include quotes in quotation marks "like this" to indicate direct quotes from your manuscript), or elaborate on this item by providing additional information not in the ms, or briefly explain why the item is not applicable/relevant for your study

Lin Z, Cheng L, Han X, Wang H, Liao Y, Guo L, Shi J, Fan B, Teopiz KM, Jawad MY, Zhang H, Chen Y, Lu C, McIntyre RS. The Effect of Internet-Based Cognitive Behavioral Therapy on Major Depressive Disorder: Randomized Controlled Trial. J Med Internet Res 2023 Sep 22;25:e42786. PMID:37738092

25) Sources of funding and other support (such as supply of drugs), role of funders

Does your paper address CONSORT subitem 25? *

Copy and paste relevant sections from the manuscript (include quotes in quotation marks "like this" to indicate direct quotes from your manuscript), or elaborate on this item by providing additional information not in the ms, or briefly explain why the item is not applicable/relevant for your study

"This work was supported by the National Natural Science Foundation of China (81761128030) and Sanming Project of Medicine in Shenzhen Nanshan (Grant No.11)."

X27) Conflicts of Interest (not a CONSORT item)

X27-i) State the relation of the study team towards the system being evaluated

In addition to the usual declaration of interests (financial or otherwise), also state the relation of the study team towards the system being evaluated, i.e., state if the authors/evaluators are distinct from or identical with the developers/sponsors of the intervention.

	1	2	3	4	5	
subitem not at all important	○	○	○	○	○	essential

Does your paper address subitem X27-i?

Copy and paste relevant sections from the manuscript (include quotes in quotation marks "like this" to indicate direct quotes from your manuscript), or elaborate on this item by providing additional information not in the ms, or briefly explain why the item is not applicable/relevant for your study

"Roger S. McIntyre has received research grant support from CIHR/GACD/National Natural Science Foundation of China (NSFC) and the Milken Institute; speaker/consultation fees from Lundbeck, Janssen, Alkermes, Neumora Therapeutics, Boehringer Ingelheim, Sage, Biogen, Mitsubishi Tanabe, Purdue, Pfizer, Otsuka, Takeda, Neurocrine, Sunovion, Bausch Health, Axsome, Novo Nordisk, Kris, Sanofi, Eisai, Intra-Cellular, NewBridge Pharmaceuticals, Viatris, Abbvie, Atai Life Sciences. Dr. Roger McIntyre is a CEO of Braxia Scientific Corp."

About the CONSORT EHEALTH checklist

As a result of using this checklist, did you make changes in your manuscript? *

- ○ yes, major changes
- ● yes, minor changes
- ○ no

What were the most important changes you made as a result of using this checklist?

Add some information that did not appeared in the manuscript

How much time did you spend on going through the checklist INCLUDING making changes in your manuscript *

A total of 4 hours was needed.

As a result of using this checklist, do you think your manuscript has improved? *

- (selected) yes
- no
- 其他:

Would you like to become involved in the CONSORT EHEALTH group?

This would involve for example becoming involved in participating in a workshop and writing an "Explanation and Elaboration" document

- yes
- (selected) no
- 其他:

清除所选内容

Any other comments or questions on CONSORT EHEALTH

您的回答

STOP - Save this form as PDF before you click submit

To generate a record that you filled in this form, we recommend to generate a PDF of this page (on a Mac, simply select "print" and then select "print as PDF") before you submit it.

When you submit your (revised) paper to JMIR, please upload the PDF as supplementary file.

Don't worry if some text in the textboxes is cut off, as we still have the complete information in our database. Thank you!

Final step: Click submit !

Click submit so we have your answers in our database!

提交 清除表单内容

切勿通过 Google 表单提交密码。

此内容不是由 Google 所创建，Google 不对其作任何担保。 举报滥用行为 - 服务条款 - 隐私权政策

Google 表单
